# Substituted salicylic acid analogs offer improved potency against multidrug-resistant *Neisseria gonorrhoeae* and good selectivity against commensal vaginal bacteria

**DOI:** 10.1038/s41598-023-41442-5

**Published:** 2023-09-02

**Authors:** Hanan Almolhim, Ahmed E. M. Elhassanny, Nader S. Abutaleb, Abdallah S. Abdelsattar, Mohamed N. Seleem, Paul R. Carlier

**Affiliations:** 1 Department of Chemistry and Virginia Tech Center for Drug Discovery, Virginia Tech, Blacksburg, VA 24061 USA; 2 Department of Biomedical Sciences and Pathobiology, Virginia-Maryland College of Veterinary Medicine, Virginia Tech, Blacksburg, VA 24061 USA; 3 Center for One Health Research, Virginia Tech, Blacksburg, VA 24061 USA; 4 Department of Pharmaceutical Sciences, University of Illinois Chicago, 833 S Wood St, Chicago, IL 60612 USA

**Keywords:** Drug discovery and development, Lead optimization, Antimicrobials, Drug discovery, Drug screening, Medicinal chemistry

## Abstract

Drug-resistant *Neisseria gonorrhoeae* represents a major threat to public health; without new effective antibiotics, untreatable gonococcal infections loom as a real possibility. In a previous drug-repurposing study, we reported that salicylic acid had good potency against azithromycin-resistant *N. gonorrhoeae*. We now report that the anti-gonococcal activity in this scaffold is easily lost by inopportune substitution, but that select substituted naphthyl analogs (**3b**, **3o** and **3p**) have superior activity to salicylic acid itself. Furthermore, these compounds retained potency against multiple ceftriaxone- and azithromycin-resistant strains, exhibited rapid bactericidal activity against *N. gonorrhoeae*, and showed high tolerability to mammalian cells (CC_50_ > 128 µg/mL). Promisingly, these compounds also show very weak growth inhibition of commensal vaginal bacteria.

## Introduction

*Neisseria gonorrhoeae*, the causative agent of the sexually transmitted disease, gonorrhea, presents a substantial, global antimicrobial resistance threat^1^. Due to the increased rates of infection as well as the prevalence of multidrug-resistant *N. gonorrhoeae* strains worldwide, in 2017 the World Health Organization (WHO) listed *N. gonorrhoeae* as a “Priority 2” or “high” tier risk to public health^1^. Dual therapy of azithromycin (AZM) and ceftriaxone has been the standard-of-care for treatment of gonococcal infections. However, due to both increasing resistance to azithromycin (> 33% in some regions), and the potent anti-commensal activity of dual therapy, the CDC removed AZM from the treatment regimen for gonorrhea in 2020^2,3^. Therefore, ceftriaxone remains the only recommended antibiotic for treatment of gonococcal infections^4^. However, there has been a concerning trend of increasing resistance to this treatment option, leading to the emergence of what is commonly referred to as ‘super gonorrhea’. This form of gonorrhea is characterized by extensive drug resistance, with high-level resistance to the recommended antibiotics, ceftriaxone and azithromycin, in addition to other classes of antibiotics^5–7^. Consequently, the world faces the real possibility of untreatable gonococcal infections^5,8^, and there is an urgent need to identify novel therapeutics against *N. gonorrhoeae*^9^.

**Figure 1:**
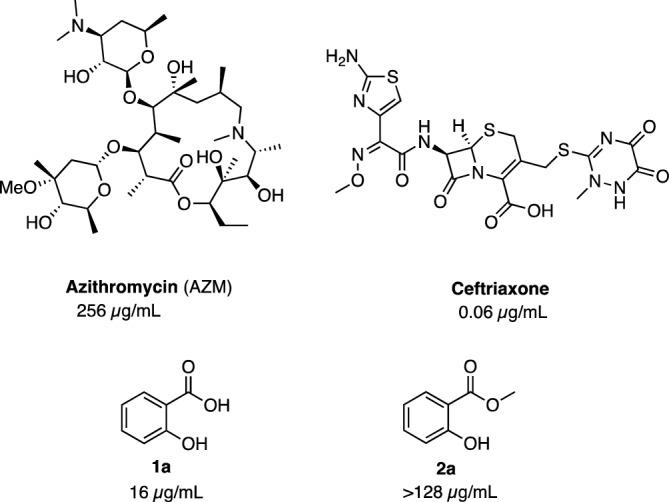
Minimum inhibitory concentrations (MICs in µg/mL) of azithromycin (AZM), ceftriaxone, salicylic acid **1a** and methyl ester **2a** against *N. gonorrhoeae* CDC-181 (AZM-resistant strain)^24^.

Drug repurposing is a popular strategy that explores new therapeutic opportunities for approved drugs with available information on their pharmacokinetic data, dosages, and toxicity^10–19^. Salicylic acid is a highly privileged chemical scaffold: its derivatives are reported to exhibit a wide range of analgesic, antioxidant, antiproliferative, and anti-cancer activities^20,21^. In addition, azo-salicylates such as sulfasalazine and olsalazine are used in the treatment of ulcerative colitis. Salicylic acid derivatives were also reported to exhibit antibacterial activity against Gram-positive bacteria and some Gram-negative bacteria such as *Escherichia coli* and *Enterobacter aerogenes*^22^*.* Of particular relevance to our work, the use of salicylic acid to treat sexually transmitted diseases (including gonorrhea) was reported as early as the nineteenth century^23^. Recently, one of us (MNS) reported that salicylic acid (**1a**) exhibited modest activity against *N. gonorrhoeae* strains including the AZM-resistant strain (CDC-181) (Fig. 1). Promisingly, this compound also retained activity (MIC = 16 µg/mL) against an *N. gonorrhoeae* strain with reduced susceptibility to ceftriaxone (CDC-194)^24^.

In the same report the methyl ester (**2a**) was found to be inactive. In this study, we sought to further explore the anti-gonococcal activity of salicylic acid analogs.

## Results and discussion

### Analog by catalog

For our initial exploration of SAR studies of salicylic acid **1a**, we pursued an “analog by catalog” strategy, and purchased 20 structurally related compounds and tested them against three multi-drug resistant *N. gonorrhoeae* strains (Table 1).

**Table 1:** MICs (μg/mL) of commercial salicylic acid derivatives and 2-naphthoic acid analogs against select strains of *N. gonorrhoeae*. See Table 7 for the resistance status of various strains.


	X	Y	*N. gonorrhoeae* isolates		
			CDC-181	CDC-166	CDC-165
AZM	na	na	> 128	1	2
1a	H	na	16	16	16
1b	5-Br	na	64	64	64
1c	5-Cl	na	64	64	64
1d	5-F	na	128	128	128
1e	5-I	na	32	32	32
1f.	4-F	na	64	128	128
1g	3-Cl	na	64	128	128
1h	6-F	na	> 128	> 128	> 128
3a	H	OH	32	32	32
3b	7-Br	OH	8	8	8
3c	4-Br	OH	16	32	32
3d	4,7-Br_2_	OH	16	32	32
3e	7-OCH_3_	OH	32	32	32
3f	1-OH	OH	64	128	128
3g	5-OH	OH	> 128	> 128	> 128
3h	7-OH	OH	> 64	> 64	> 64
3i	7-Br	OCH_3_	64	64	64
3j	1-OH	H	16	16	16
3k	6-OH	H	> 64	> 64	> 64
3l	H	NH_2_	16	16	16
3m	6-NH_2_	H	64	64	64

Most of these compounds showed lower potency than **1a** against the strains tested. Specifically, mono-halogenation at C3–C6 (cf. **1b**–**1h**) significantly decreased potency, a point we will return to at the end of this report. Moving onto naphthoic acid derivatives, 3-hydroxy-2-naphthoic acid **3a** was slightly less potent than **1a**, but addition of a bromine at C7 resulted in the more potent compound **3b** (MICs = 8 μg/mL vs 16 μg/mL for **1a**). Regioisomer **3c** was slightly less potent than **3b**, and other substituted 3-hydroxy-2-naphthoic acids **3d**–**h** were less potent than **3b**. The importance of the hydroxy group of **3b** is shown by methyl ether **3i,** which is significantly less potent. Finally, 1-hydroxy-2-naphthoic acid **3j,** 6-hydroxy-2-naphthoic acid **3k**, and amino-substituted 2-naphthoic acids **3l** and **3m** were less potent than **3b**.

### Synthesis and evaluation of structural analogs of 3b

**Figure 2:**
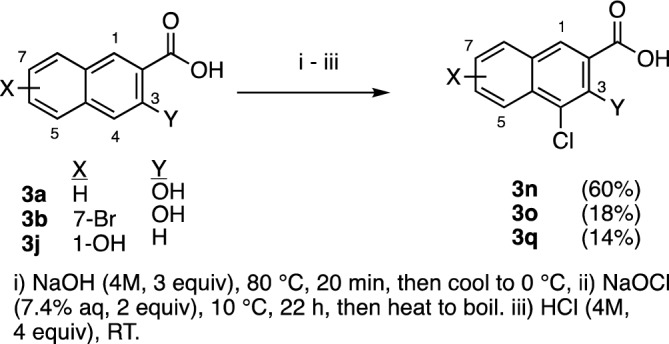
Synthesis of 4-chloro derivatives of **3a**, **3b**, **3j**.

**Figure 3:**
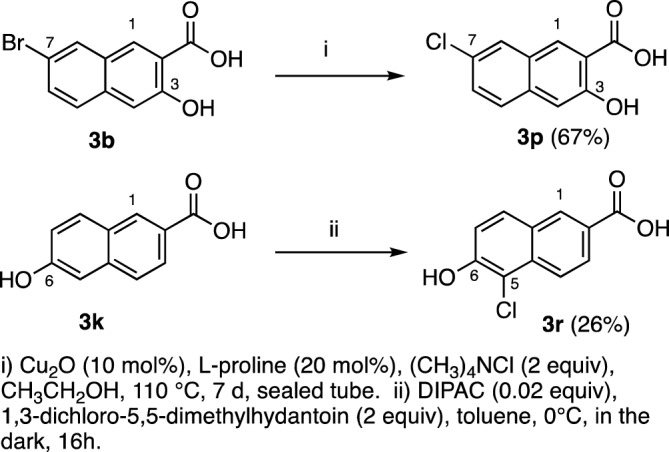
Synthesis of additional chlorinated 2-naphthoic acid analogs.

Focusing on the favorable activity of **3b**, we synthesized several structural additional compounds**.** Firstly, since 4-bromo-3-hydroxy-2-naphthoic acid **3c** was nearly as potent as **3b** against *N. gonorrhoeae* CDC-181 strain, we prepared its chloro analog **3n** by electrophilic chlorination (Fig. 2)^25^. Similarly, since 4,7-dibromo-3-hydroxy-naphthoic acid **3d** was nearly as potent as **3b** against this strain, **3o** was prepared from **3b**. Lastly **3q** was prepared by chlorination of **3j**. Note that the moderate to low yields reported for these compounds reflect the need for multiple recrystallizations needed to achieve ≥ 95% purity. We then prepared **3p**, the 7-chloro analog of **3b** by copper-catalyzed Finkelstein reaction (Fig. 3) ^26^. Lastly, the inactive compound **3m** was converted to the 5-chloro derivative **3r** by electrophilic chlorination, as described in Fig. 3^27^.

As seen in Table 2, like **3b**, all of the halogenated 2-naphthoic acids bearing a hydroxy at C3 or C1 (i.e. **3n**–**3q**) had improved potency relative to **1a** (Table 1). Halogenated 2-napthoic acid **3r**, which bears a hydroxy group at C6 rather than C3 or C1, is not potent.

**Table 2:** MICs (μg/mL) of synthesized halogenated 2-naphthoic acid analogs related to 3b.


	X	Y	*N. gonorrhoeae* isolates		
			CDC-181	CDC-166	CDC-165
AZM	na	na	> 128	1	2
3n	4-Cl	OH	16	8	8
3o	4-Cl, 7-Br	OH	8	8	8
3p	7-Cl	OH	8	8	8
3q	4-Cl, 1-OH	H	8	8	8
3r	5-Cl, 6-OH	H	> 64	32	32

**Figure 4:**
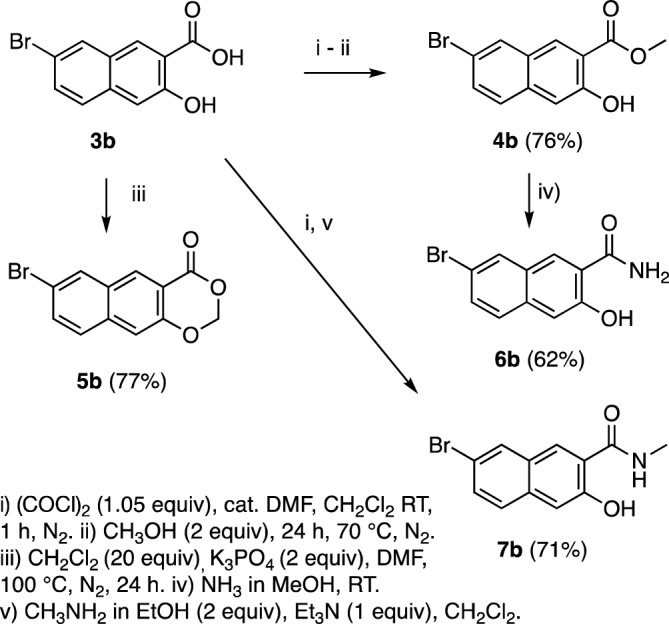
Synthesis of carboxyl derivatives of **3b**.

**Figure 5:**
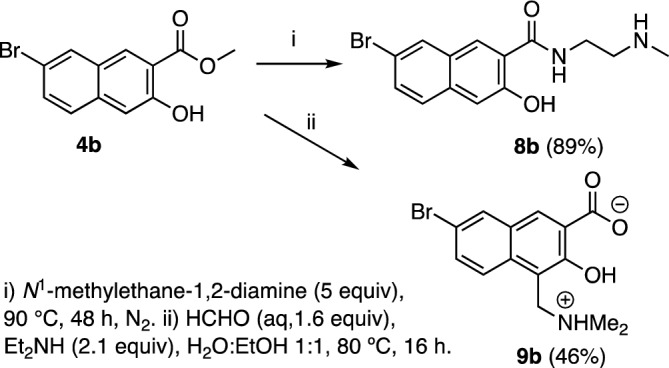
Synthesis of basic or zwitterionic derivatives of **3b**.

To assess the importance of the carboxyl group to the antibacterial activity of **3b**, the methyl (**4b**) and cyclic methylene ester (**5b**) were prepared, as were the 1° (**6b**) and methyl amides (**7b**) (Fig. 4). Further, since it has been shown that drug uptake by Gram-negative bacteria can sometimes be substantially improved by the addition of basic amine functionality^28, 29^, we synthesized basic amine-bearing amide **8b** and Mannich base **9b** from methyl ester **4b** (Fig. 5)**.**

As depicted in Table 3, the ester (**4b**, **5b**) and amide (**6b**-**8b**) derivatives of **3b** significantly lost potency, as did the zwitterionic Mannich base **9b**.

**Table 3:** MICs (μg/mL) of synthesized analogs of 3a.


	X	*N. gonorrhoeae* isolates		
		CDC-181	CDC-166	CDC-165
AZM	na	> 128	1	2
4b	OMe	> 64	> 64	> 64
5b	na	> 64	> 64	> 64
6b	NH_2_	> 64	> 64	> 64
7b	NMe_2_	> 64	> 64	> 64
8b	CH_2_CH_2_NHMe	64	64	> 64
9b	na	64	> 64	> 64

### Exploration of 4,5-disubstituted salicylic acids

Knowing that naphthyl rings and naphthols are susceptible to CYP450 oxidation, we turned our attention to 4,5-disubstituted salicylic acids as potential isosteres for **3b**. To jump-start this exploration, we purchased four 4,5-disubstituted salicylic acid derivatives **1i–l** and screened them against the three *N. gonorrhoeae* isolates (Table 4).

**Table 4:** MICs (μg/mL) of commercial and synthesized 4,5-disubstituted salicylic acids.


	X	Y	*N. gonorrhoeae* isolates		
			CDC-181	CDC-166	CDC-165
AZM	na	na	> 128	1	2
1i	Cl	Cl	16	8	8
1j	Cl	Br	16	8	8
1k	CH_3_	Cl	32	16	16
1l	CH_3_	Br	32	16	16
1m	F	Cl	32	16	16
1n	F	Br	32	16	16

Interestingly, compound **1i** and **1j** appeared to be nearly as potent as **3b** with MIC = 8 µg/mL against *N. gonorrhoeae* CDC-166 and CDC-165 stains and MIC = 16 µg/mL against *N. gonorrhoeae* CDC-181 strain. It is noted that these compounds are significantly more potent than the mono-halogenated salicylic acids described in Table 1, including 5-bromosalicylic acid **1b** and 5-chlorosalicylic acid **1c** (MIC = 64 µg/mL). Thus, the presence of a second halogen (chlorine) at C4 increased potency by 4-fold. However, installation of a methyl group at position 4 in place of Cl negatively affected potency (Table 3, cf. **1k** and **1i**, **1l** and **1j**).

**Figure 6:**
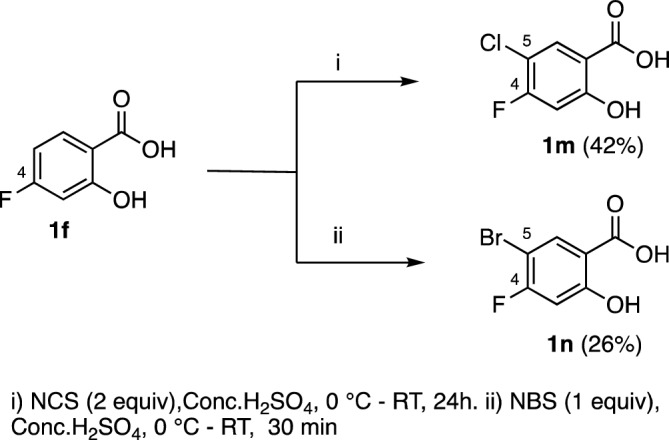
C5-halogenation of 4-fluorosalicylic acid **1f**.

To further explore this scaffold, we synthesized two additional 3,4-dihalogenated salicylic acid derivatives from 5-fluorosalicylic acid **1f**. Compounds **1m–n** were prepared from **1f** via electrophilic halogenation as described in Fig. 6^30^.

We concluded that similar to methyl substitution, fluorine substitution at position 4 seems to negatively affect potency of salicylic acid derivatives (Table 4, cf. **1m** and **1i**, **1n** and **1j**).

### Anti-gonococcal activity of salicylic acid analogs

The antibacterial activity of the most promising salicylic acid derivatives (**3b**, **3o**, **3p**, **3q**, **1i** and **1j**) was investigated against a panel of multidrug-resistant *N. gonorrhoeae* strains including ceftriaxone- and azithromycin-resistant strains (Table 5). The panel of strains tested contained 6 WHO reference strains with well-characterized genetic and phenotypic markers^31^. Salicylic acid analogs maintained consistent potency against the tested strains. Compounds **3b**, **1i** and **1j** displayed the most potent activity, effectively inhibiting the tested strains at concentrations ranging from 2 to 8 µg/mL. Particularly intriguingly was their activity against azithromycin-resistant *N. gonorrhoeae* strains (WHO-P, WHO-U, WHO-V and WHO-Z), with MIC values ranging from 2 to 8 µg/mL. Moreover, the analogs demonstrated the comparable activity against ceftriaxone-resistant strains, including WHO-X, WHO-Y and WHO-Z, with MICs ranging from 2 to 32 µg/mL. To validate the MIC results, *N. gonorrhoeae* CDC-10328, a reference strain for antimicrobial susceptibility testing, was included among the tested strains. The MICs of the control antibiotics, azithromycin and ceftriaxone, against this strain matched the modal MICs previously reported^32^. These findings underscore the promising potential of the salicylic acid analogs as effective anti-gonococcal agents, even against multidrug-resistant strains (Table 5).

**Table 5:** MICs (µg/mL) of select salicylic acid analogs against a panel of *N. gonorrhoeae* strains.

*N. gonorrhoeae* strains	Test agents							
	AZM	CEF	3b	3o	3p	3q	1i	1j
WHO-P	8	0.002	4	16	4	16	4	8
WHO-U	8	0.001	4	2	4	16	4	8
WHO-V	> 64	0.03	2	16	2	16	2	2
WHO-X	0.5	1	8	16	16	16	8	8
WHO-Y	1	1	2	32	8	16	8	4
WHO-Z	2	0.5	8	16	16	32	8	8
FA1090	0.125	0.002	8	8	8	8	8	8
CDC-10328	0.06	0.002	2	8	4	16	2	2

AZM, azithromycin; CEF, ceftriaxone.

### Killing kinetics of salicylic acid analogs

**Figure 7:**
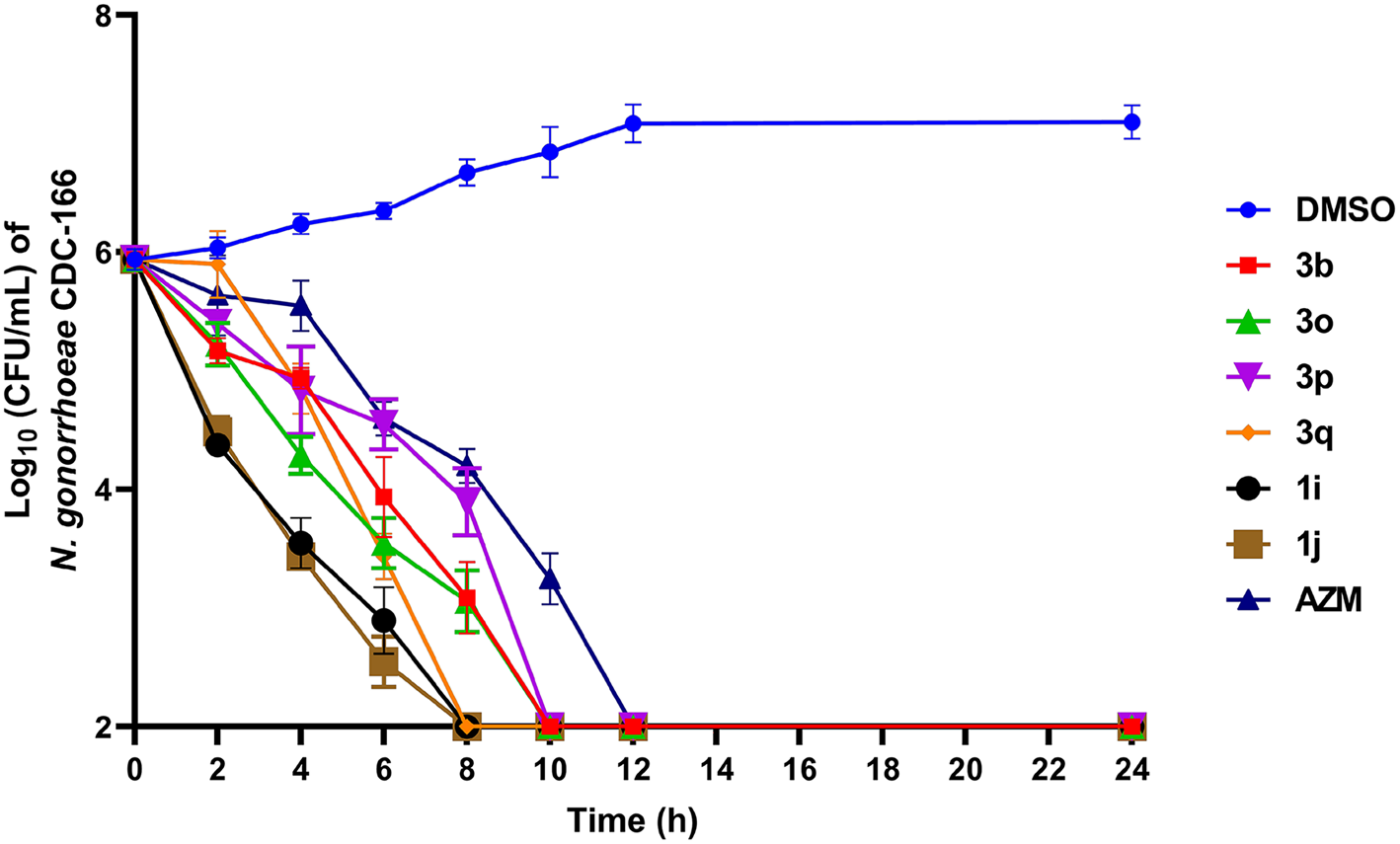
Time-kill kinetics of salicylic acid analogs against *N. gonorrhoeae* CDC-166. A log-phase bacterial culture was exposed to either compounds **3b**, **3o**, **3p**, **3q**, **1i**, **1j** or azithromycin (at 5 × MIC). DMSO (solvent) served as a negative control. The error bars represent standard deviation values for each test agent.

Following the confirmation of potent antibacterial activity of the analogs against multidrug-resistant *N. gonorrhoeae* strains, we assessed their killing kinetics against *N. gonorrhoeae* CDC-166 (Fig. 7). Compounds **3q**, **1i** and **1j** displayed a rapid bactericidal activity against *N. gonorrhoeae*, completely eradicating the high bacterial burden below the detection limit within 8 h. This was superior to the activity of azithromycin which exerted its bactericidal activity after 10 h and required 12 h to completely eradicate the bacterial burden. Compounds **3b**, **3o** and **3p** were as effective as azithromycin in their bactericidal activity where they reduced the bacterial count below the detection limit within 10 h (Fig. 7). The rapid bactericidal activity observed in these salicylic acid analogs is a highly desirable trait for anti-gonococcal agents. It not only limits the spread of infection but also plays a pivotal role in reducing the development of bacterial resistance and preventing disease progression^33,34^.

### Effects on commensal bacteria

One of the disadvantages of the anti-gonococcal standard of care therapeutics is that they inhibit growth of both gonococci and commensal bacteria^35^. The vaginal microbiota are the primary defense line against *N. gonorrhoeae* infection. They compete with *N. gonorrhoeae* for adhesion to the genitourinary tract in addition to creating an acidic environment that prevents gonococcal colonization. Hence, disruption of the healthy microbiota present in the genitourinary tract could enhance the gonococcal infection^36^. The microbiome of the genitourinary tract is dominated by *Lactobacillus.* It was reported that *Lactobacillus* could significantly reduce *N. gonorrhoeae* viability by creating acidic environment, producing bacteriocins, releasing biosurfactants, co-aggregating with gonococci, and reducing the gonococcal adhering to epithelial cells^37^. We evaluated the antibacterial activity of the prioritized salicylic acid derivatives (**3b**, **3o**, **3p**, **3q**, **1i** and **1j**) alongside azithromycin, against representative members of the vaginal microbiota of the *Lactobacillus* species. As reported previously, azithromycin inhibited growth of *Lactobacillus* strains with MIC values of ≤ 1 µg/ml^38,39^. To our delight, the salicylic acid analogs, **3b**, **3o**, **3p**, **3q**, **1i** and **1j** did not inhibit growth of *Lactobacillus* strains at concentrations as high as 128–256 µg/mL (Table 6), which indicates the high selectivity of the salicylic acid analogs against *N. gonorrhoeae* over the vaginal microbiota.

**Table 6:** MICs (µg/mL) of AZM and select salicylic acid analogs against *N. gonorrhoeae* and different *Lactobacillus* species.


Bacterial strains	Test agents						
	AZM	3b	3o	3p	3q	1i	1j
*N. gonorrhoeae* 181 strain	> 128	8	8	8	8	16	16
*L. gasseri* HM-400	≤ 1	128	128	256	128	> 256	256
*L. gasseri* HM-642	≤ 1	> 256	> 256	> 256	> 256	> 256	> 256
*L. gasseri* HM-644	≤ 1	128	> 256	256	> 256	128	128
*L. gasseri* HM-403	≤ 1	256	> 256	256	> 256	256	256
*L. crispatus* HM-638	≤ 1	128	> 256	128	128	128	128
*L. jensenii* HM-640	≤ 1	128	256	256	256	128	128
*L. jensenii* HM-105	≤ 1	> 256	> 256	> 256	> 256	> 256	> 256
*L. jensenii* HM-639	≤ 1	256	> 256	> 256	256	256	256

### Cytotoxicity of salicylic acid analogs.

**Figure 8:**
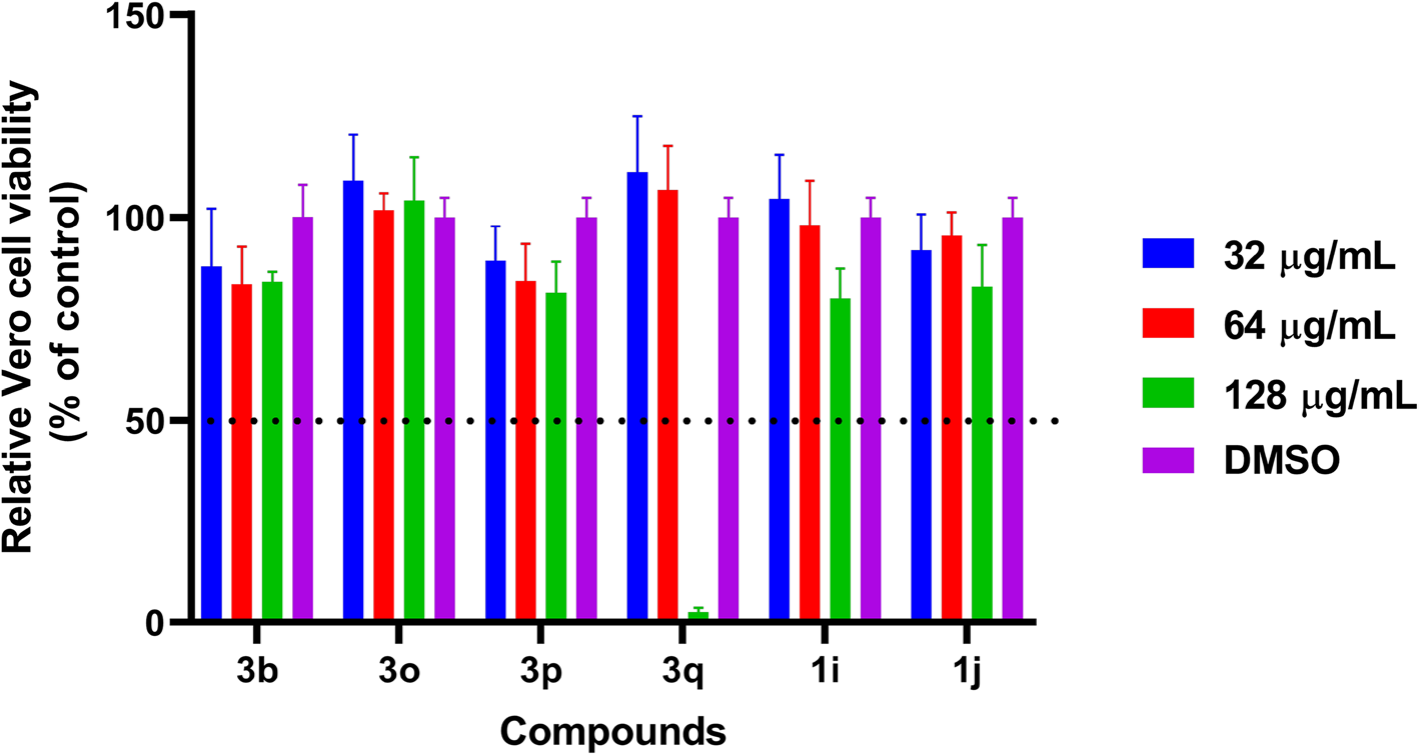
In vitro cytotoxicity assessment of salicylic acid analogs against kidney epithelial cells (Vero). Compounds were incubated with Vero cells for 24 h. Then, cells were incubated with MTS reagent before measuring absorbance values. Results are presented as percent viable cells relative to DMSO (negative control). The absorbance values represent an average of four samples analyzed for each compound. Error bars represent standard deviation values.

An essential attribute in the development of new drugs is their lack of toxicity. To assess this aspect, we evaluated the prioritized salicylic acid analogs (**3b**, **3o**, **3p**, **3q**, **1i** and **1j)** for their cytotoxicity against kidney epithelial (Vero) cells (Fig. 8). We aimed to identify any potential cytotoxicity effects on mammalian cells. Encouragingly, the compounds exhibited an excellent safety profile and demonstrated high tolerability to Vero cells. The CC_50_, representing the concentration required to reduce cell viability by 50%, were found to be higher than 128 μg/mL for all analogs, with the exception of **3q,** which exhibited reduced viability at 128 μg/mL. Notably, all the cells were viable at 64 μg/mL (Fig. 8). These results highlight the favorable cytotoxicity profile of the salicylic acid analogs, signifying their potential as safe and non-toxic candidates for further drug development.

## Conclusion

In this report, we demonstrated that the significant anti-gonococcal activity of salicylic acid **1a** against AZM-resistant *N. gonorrhoeae* (MIC = 16 µg/mL) is not a simple characteristic of its *o*-hydroxy benzoic acid moiety. Inopportune substitution of the benzo ring (cf. Table 1) greatly reduced anti-gonococcal potency. In contrast, we found that appropriately substituted 3-hydroxy-2-naphthoic acids (**3b**, **3o**, **3p**) and 1-hydroxy-2-naphthoic acid (**3q**) have twice the anti-gonococcal potency of **1a**. In addition, these compounds, and less-potent 4,5-dihalogenated salicylic acids (**1i**, **1j**) are significantly less toxic to commensal vaginal bacteria than **AZM**. Moreover, these compounds exhibited rapid bactericidal activity against *N. gonorrhoeae,* were tolerable to Vero cells, and retain activity against ceftriaxone- and azithromycin-resistant strains. Thus, although greater anti-gonococcal potency is required for a new therapeutic, the other favorable properties exhibited by the simple molecular scaffolds displayed in Table 6 suggest they warrant further development.

## Materials and methods

### Procurement and synthesis of tested compounds

Compounds **1a**–**h** and **3a**–**m** were purchased from a variety of suppliers. In each case, ^1^H NMR was used to confirm the identity and purity of the compound. Compounds **3n**–**r**, **4**–**9b**, and **1m**–**n** were prepared as described above. Full synthetic procedures and analytical characterization data (^1^H, ^13^C NMR, HRMS) are provided in the Supplementary Information.

### Bacterial strains, media, reagents and antibacterial assay procedures

*N. gonorrhoeae* strains (Table 7) used in the study were clinical isolates obtained from the CDC, the WHO and the American Type Culture Collection (ATCC). *Lactobacillus* isolates were obtained from the Biodefense and Emerging Infections Research Resources Repository (BEI Resources). Azithromycin (AZM) and ceftriaxone (CEF) were purchased from TCI America, (Portland, OR, USA). Media and reagents were purchased commercially: brucella broth, IsoVitaleX, chocolate II agar plates and MRS broth (Becton, Dickinson and Company, Cockeysville, MD, USA), yeast extract and dextrose (Fisher Bioreagents, Fairlawn, NJ, USA), protease peptone (Oxoid, Lenexa, KS, USA), hematin, pyridoxal, and nicotinamide adenine dinucleotide (NAD) (Chem-Impex International, Wood Dale, IL, USA), Eagle's Minimum Essential Medium and fetal bovine serum (Corning, Manassas, VA, USA) and phosphate-buffered saline (PBS) (Corning, Manassas, VA, USA). Compounds were prepared as stock solutions in DMSO, diluted in media, to give a final DMSO concentration of less than 2%.

**Table 7:** Bacterial isolates used in the study.

Isolate	Description
*N. gonorrhoeae* CDC-181	Resistant to tetracycline and azithromycin
*N. gonorrhoeae* CDC-166	Resistant to tetracycline, penicillin, and ciprofloxacin
*N. gonorrhoeae* CDC-165	Resistant to tetracycline, penicillin, and ciprofloxacin
*N. gonorrhoeae* WHO-P	Isolated in USA, resistant to azithromycin and tetracycline
*N. gonorrhoeae* WHO-U	Clinical isolate from a pharyngeal specimen in Sweden, 2011. Resistant to erythromycin and azithromycin
*N. gonorrhoeae* WHO-V	Clinical isolate from a urethral specimen in Sweden, 2012. Resistant to erythromycin, azithromycin, ciprofloxacin, tetracycline and sulfamethoxazole
*N. gonorrhoeae* WHO-X	Isolated from a female pharynx specimen in Kyoto, Japan, 2009. Resistant to cefixime, ceftriaxone, ciprofloxacin, penicillin, and tetracycline
*N. gonorrhoeae* WHO-Y	Isolated from a urethral specimen of a 50 years old male in Quimper, France, 2010. Resistant to cefixime, ceftriaxone, ciprofloxacin, and tetracycline
*N. gonorrhoeae* WHO-Z	Isolated from a female genital swab in Australia, 2013. Resistant to azithromycin, cefixime, ceftriaxone, ciprofloxacin, penicillin, and tetracycline
*N. gonorrhoeae* FA1090	Isolated from a female patient with disseminated gonococcal infection. Resistant to streptomycin
*N. gonorrhoeae* CDC-10328	Quality control strain for susceptibility testing. Resistant to penicillin and ciprofloxacin
*L. gasseri* HM-400	Isolated from human patient’s mid-vaginal wall in Richmond, VA, USA
*L. gasseri* HM-642	Vaginal isolate from a healthy U.S. woman, in 2007
*L. gasseri* HM-644	Vaginal mucosal isolate of a healthy U.S. woman of child-bearing age
*L. gasseri* HM-403	Isolated from human patient’s mid-vaginal wall, Richmond, VA, USA
*L. crispatus* HM-638	Vaginal isolate from a healthy Chinese woman, in 2007
*L. jensenii* HM-640	Isolated from the vaginal mucosa of a healthy Chinese woman in 2007
L. jensenii HM-105	A human vaginal isolate obtained from Texas
*L. jensenii* HM-639	Isolated from the vaginal mucosa of a healthy U.S. woman in 2007

### Antibacterial activity of salicylic acid analogs against *N. gonorrhoeae* strains

The determination of MICs for compounds was carried out as described previously^11,13,40–42^. Briefly, *N. gonorrhoeae* strains were grown overnight on chocolate agar plates. A bacterial solution equivalent to 1.0 McFarland standard was prepared and diluted in brucella broth supplemented with yeast extract, dextrose, protease-peptone, NAD, pyridoxal, hematin and IsoVitaleX to reach about 1 × 10^6^ CFU/mL. Serial dilutions of test agents were incubated with bacteria at 37 °C in presence of 5% CO_2_ for 24 h before recording the MICs as observed visually. MICs reported are the lowest concentrations of each test agent that could completely inhibit the visual bacterial growth.

### Killing kinetics analysis of salicylic acid analogs against *N. gonorrhoeae*

In order to determine if salicylic acid analogs exhibit bacteriostatic or bactericidal activity against *N. gonorrhoeae*, a standard time-kill assay was performed against *N. gonorrhoeae* CDC-166, as described previously^11,38,39^. Briefly, a log-phase culture of *N. gonorrhoeae* was diluted to ∼ 10^6^ CFU/mL in the supplemented brucella broth. Test agents were then added (at 5 × MIC in triplicates) and incubated with bacteria at 37 °C in presence of 5% CO_2_. An aliquot from each treatment was collected after the corresponding times of incubation and subsequently serially diluted and plated onto chocolate II agar plates. Plates were incubated for 24 h at 37 °C before viable CFU/mL was determined.

### In vitro cytotoxicity evaluation of the salicylic acid analogs

The in vitro cytotoxicity assessment for salicylic acid analogs was carried out against kidney fibroblast (Vero) cells as described elsewhere^43–48^. Briefly, compounds were incubated with Vero cells for 24 h and DMSO served as a negative control. Then, cells were incubated with MTS reagent for 3 h before measuring absorbance values (OD_490_).

### Antibacterial activity of salicylic acid analogs against genitourinary tract normal microbiota strains

The MICs of salicylic acid analogs against representative commensal members of the genitourinary tract were determined, as described elsewhere^16,45,49–52^. Lactobacilli were grown onto MRS agar for 48 h at 37 °C in presence of 5% CO_2_. A bacterial solution equivalent to 0.5 McFarland standard was diluted in MRS broth to achieve a bacterial concentration of ~ 5 × 10^5^ CFU/mL and incubated with serial dilutions of the test agents as described, before recording the MIC values by visual inspection of growth.

### Supplementary Information

Supplementary Information.

## Data Availability

The datasets used and/or analyzed during the current study are available from the corresponding author on reasonable request.
