# Supplementary material for: Substituted salicylic acid analogs offer improved potency against multidrug-resistant Neisseria gonorrhoeae and good selectivity against commensal vaginal bacteria
Source: Sci Rep. 2023 Sep 2;13:14468. doi: 10.1038/s41598-023-41442-5 (PMC10475031; doi:10.1038/s41598-023-41442-5)
Supplement: Supplementary file 1 — Supplementary Information. [file 41598_2023_41442_MOESM1_ESM.pdf]

Supplementary Material for the manuscript entitled

Substituted salicylic acid analogs offer improved potency against multidrug-resistant *Neisseria gonorrhoeae* and good selectivity against commensal vaginal bacteria

Hanan Almolhim[a], Ahmed E.M. Elhassanny[b,c], Nader S. Abutaleb[b,c], Mohamed N. Seleem[b,c], Paul R. Carlier[a,d]*

[a]Department of Chemistry and Virginia Tech Center for Drug Discovery, Virginia Tech, Blacksburg, VA 24061.

[b]Department of Biomedical Sciences and Pathobiology, Virginia-Maryland College of Veterinary Medicine, Virginia Tech, Blacksburg, VA 24061.

[c]Center for One Health Research, Virginia Tech, Blacksburg, VA 24061.

[d]Department of Pharmaceutical Sciences, University of Illinois at Chicago, 833 S Wood St, Chicago, IL 60612.

Table of Contents

Section	Description	Page
1	Confirmation of purity and identity of purchased compounds	S1
2	Synthetic procedures and analytical tabulations for synthesized analogs	S8
3	References	S16
4	^{1}H NMR spectra of purchased compounds, and ^{1}H and ^{13}C NMR spectra of synthesized compounds.	S18

1. Confirmation of purity and identity of purchased compounds

Compounds were purchased from a variety of suppliers. In each case, ^{1}H NMR was used to confirm the identity and purity of the compound. With the exception of **3h** (which contained 10 mol% **3a**), all compounds were >95% pure. For convenience we list our observed ^{1}H NMR tabulations below. In several cases we obtained additional supporting data. Note that in many cases, the *o*-hydroxycarboxylic acid protons give a merged, extremely broad signal that is only visible under high vertical expansion of the baseline; in other cases the signals cannot be reliably detected even then.

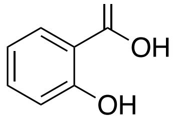

1a

*2-hydroxybenzoic acid (**1a**, salicylic acid)*

^{1}H NMR (400 MHz, d_6-DMSO) δ 13.7 (br s, 1H), 11.4 (br s, 1H), 7.8 (ddd, J = 7.9, 1.8, 0.5 Hz, 1H), 7.5 (ddd, J = 8.4, 7.2, 1.8 Hz, 1H), 7.0 – 6.9 (m, 2H).

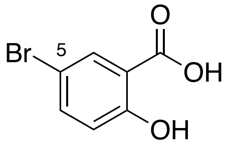

1b

*5-bromo-2-hydroxybenzoic acid (**1b**)*

^{1}H NMR (400 MHz, d_6-DMSO) δ 12.4 (br s, 2H), 7.8 (d, J = 2.6 Hz, 1H), 7.6 (dd, J = 8.9, 2.6 Hz, 1H), 6.9 (d, J = 8.8 Hz, 1H).

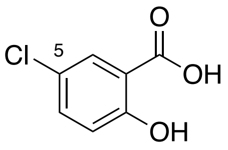

1c

*5-chloro-2-hydroxybenzoic acid (**1c**)*

^{1}H NMR (400 MHz, d_6-DMSO) δ 12.3 (br s, 2H), 7.7 (d, J = 2.8 Hz, 1H), 7.5 (dd, J = 8.9, 2.7 Hz, 1H), 7.0 (d, J = 8.9 Hz, 1H).

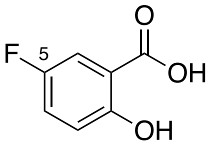

1d

*5-fluoro-2-hydroxybenzoic acid (**1d**)*

^{1}H NMR (400 MHz, d_6-DMSO) δ12.3 (br s, 2H), 7.5 (dd, J = 8.9, 3.2 Hz, 1H), 7.4 (ddd, J = 9.1, 8.2, 3.2 Hz, 1H), 7.0 (dd, J = 9.1, 4.6 Hz, 1H).

^{19}F NMR (376 MHz, d_6-DMSO) δ -124.3 (d, J = 4.4 Hz).

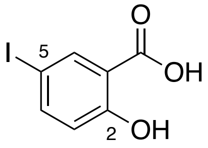

1e

*2-hydroxy-5-iodobenzoic acid (**1e**)*

^{1}H NMR (400 MHz, d_6-DMSO) δ 11.8 (br s, 2H), 7.8 (d, J = 2.6 Hz, 1H), 7.6 (dd, J = 8.9, 2.6 Hz, 1H), 6.9 (d, J = 8.8 Hz, 1H).

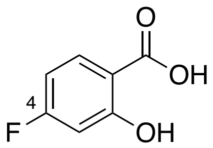

1f

*4-fluoro-2-hydroxybenzoic acid (**1f**)*
^{1}H NMR (400 MHz, d_6-DMSO) δ 11.7 (br s, 2 H), 7.9 (dd, J = 8.8, 6.8 Hz, 1H), 6.8 (dd, J = 10.8, 2.5 Hz, 1H), 6.7 (dd, J = 8.7, 2.6 Hz, 1H); the carboxylic acid proton signal was not detected.
^{19}F NMR (376 MHz, d_6-DMSO) δ -102.3 – -102.7 (m).

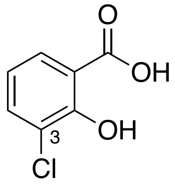

1g

*3-chloro-2-hydroxybenzoic acid (**1g**)*
^{1}H NMR (400 MHz, d_6-DMSO) δ 12.5 (br s, 2H), 7.8 (dd, J = 7.9, 1.6 Hz, 1H), 7.7 (dd, J = 7.9, 1.6 Hz, 1H), 6.9 (t, J = 7.9 Hz, 1H).

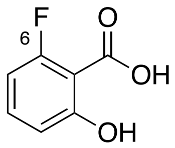

1h

*6-fluoro-2-hydroxybenzoic acid (**1h**)*
^{1}H NMR (400 MHz, d_6-DMSO) δ 12.3 (br s, 2H), 7.4 (td, J = 8.3, 6.5 Hz, 1H), 6.8 (dt, J = 8.4, 1.0 Hz, 1H), 6.7 (ddd, J = 10.4, 8.3, 1.0 Hz, 1H).
^{19}F NMR (376 MHz, d_6-DMSO) δ -110.5 (dd, J = 10.3, 6.3 Hz).

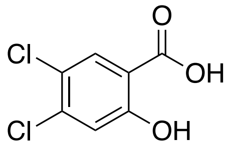

1i

*4,5-dichloro-2-hydroxybenzoic acid (**1i**)*
^{1}H NMR (500 MHz, d_6-DMSO) δ 12.2 (br s, 2H), 7.9 (s, 1H), 7.3 (s, 1H).
^{13}C NMR (126 MHz, d_6-DMSO) δ 169.9, 159.8, 137.3, 131.1, 121.0, 119.2, 114.2.
HRMS calculated for $C_7H_3Cl_2O_3^-$ 204.9465, found 204.9461.

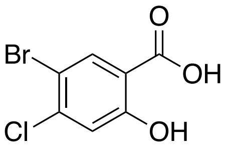

1j

*5-bromo-4-chloro-2-hydroxybenzoic acid (**1j**)*
^{1}H NMR (500 MHz, d_6-DMSO) δ 12.3 (s, 2H), 8.0 (s, 1H), 7.3 (s, 1H).
^{13}C NMR (126 MHz, d_6-DMSO) δ 169.8, 160.4, 139.2, 134.3, 119.1, 114.5, 110.2.
HRMS calculated for $C_7H_3BrClO_3^-$ 248.8960, found 248.8957.

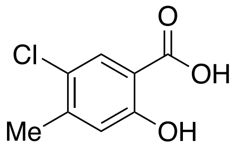

1k

*5-chloro-2-hydroxy-4-methylbenzoic acid (**1k**)*
^{1}H NMR (400 MHz, d_6-DMSO) δ 11.6 (br s, 2H), 7.7 (s, 1H), 7.0 (s, 1H), 2.3 (s, 3H); the carboxylic acid proton was not seen.

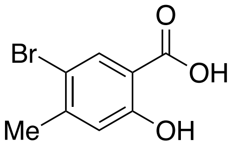

1l

*5-bromo-2-hydroxy-4-methylbenzoic acid (**1l**)*
^{1}H NMR (400 MHz, d_6-DMSO) δ 11.3 (br s, 2 H), 7.9 (s, 1H), 7.0 (s, 1H), 2.3 (s, 3H); the carboxylic acid proton was not seen.

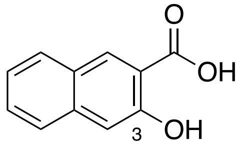

3a

*3-hydroxy-2-naphthoic acid (**3a**)*
^{1}H NMR (400 MHz, d_6-DMSO) δ 11.6 (br s, 2 H), 8.5 (s, 1H), 8.0 (d, J = 8.3 Hz, 1H), 7.8 (d, J = 8.4 Hz, 1H), 7.5 (ddd, J = 8.2, 6.8, 1.3 Hz, 1H), 7.4 – 7.3 (m, 1H), 7.3 (s, 1H); the carboxylic acid proton was not seen.

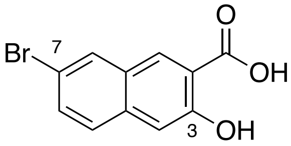

3b

*7-bromo-3-hydroxy-2-naphthoic acid (**3b**)*

^{1}H NMR (400 MHz, d_6-DMSO) δ 11.1 (br s, 2H), 8.5 (s, 1H), 8.3 (d, *J* = 2.7 Hz, 1H), 7.7 (d, *J* = 8.8 Hz, 1H), 7.6 (dd, *J* = 8.9, 2.0 Hz, 1H), 7.4 (s, 1H).

^{13}C NMR (126 MHz, d_6-DMSO) δ 171.2, 156.4, 135.6, 131.73, 131.72, 130.8, 128.3, 127.8, 116.5, 116.4, 111.1.

HRMS calculated for $C_{11}H_6BrO_3^-$ 264.9506, found 264.9503.

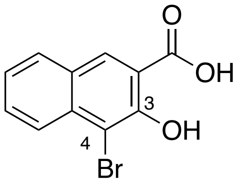

3c

*4-bromo-3-hydroxy-2-naphthoic acid (**3c**)*

^{1}H NMR (400 MHz, d_6-DMSO) δ 12.2 (br s, 2H), 8.6 (s, 1H), 8.1 (d, *J* = 8.2 Hz, 1H), 8.0 (d, *J* = 8.6 Hz, 1H), 7.8 – 7.7 (m, 1H), 7.5 (ddd, *J* = 8.1, 6.9, 1.2 Hz, 1H).

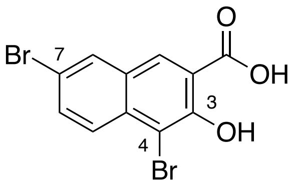

3d

*4,7-dibromo-3-hydroxy-2-naphthoic acid (**3d**)*

^{1}H NMR (400 MHz, d_6-DMSO) δ 8.6 (s, 1H), 8.4 (d, *J* = 2.1 Hz, 1H), 8.0 (d, *J* = 9.1 Hz, 1H), 7.8 (dd, *J* = 9.1, 2.1 Hz, 1H).

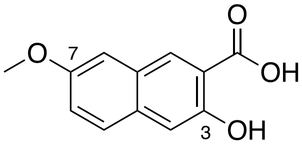

3e

*3-hydroxy-7-methoxy-2-naphthoic acid (**3e**)*

^{1}H NMR (400 MHz, d_6-DMSO) δ 13.7 (br s, 1H), 11.0 (br s, 1H), 8.4 (s, 1H), 7.7 (d, *J* = 9.0 Hz, 1H), 7.4 (d, *J* = 2.6 Hz, 1H), 7.3 (s, 1H), 7.2 (dd, *J* = 9.0, 2.6 Hz, 1H), 3.8 (s, 3H).

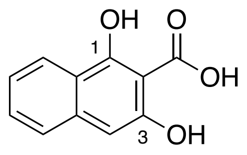

3f

*1,3-dihydroxy-2-naphthoic acid (**3f**)*

^{1}H NMR (400 MHz, d_6-DMSO) δ 12.7 (br s, 3H), 8.1 – 8.0 (m, 1H), 7.6 (d, *J* = 8.2 Hz, 1H), 7.5 (ddd, *J* = 8.2, 6.7, 1.3 Hz, 1H), 7.2 (ddd, *J* = 8.2, 6.7, 1.3 Hz, 1H), 6.6 (s, 1H).

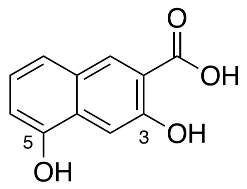

3g

*3,5-dihydroxy-2-naphthoic acid (**3g**)*

^{1}H NMR (400 MHz, d_6-DMSO) δ 11.2 (br s, 1H),10.1 (s, 1H), 8.4 (s, 1H), 7.4 (s, 1H), 7.4 (d, *J* = 8.7 Hz, 1H), 7.2 (dd, *J* = 8.3, 7.4 Hz, 1H), 6.9 (dd, *J* = 7.5, 1.0 Hz, 1H); the carboxylic acid proton signal was not detected.

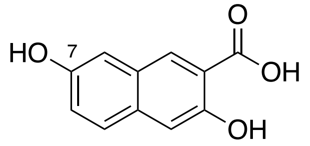

3h

*3,7-dihydroxy-2-naphthoic acid (**3h**)*

In the ^{1}H NMR spectrum we observe **3h** and **3a** in 9:1 ratio. Since **3a** was only weakly active in the antibacterial assay we assayed this sample of **3h**, in the hope that the major constituent might prove active.

^{1}H NMR (400 MHz, d_6-DMSO) δ 13.7 (br s, 1H), 10.8 (br s, 1H), 9.6 (s, 1H), 8.5 (s, 0.1H), 8.3 (s, 0.9H), 8.0 (d, *J* = 8.2 Hz, 0.1H), 7.8 (d, *J* = 7.7 Hz, 0.1H), 7.6 (d, *J* = 9.3 Hz, 0.9H), 7.5 (ddd, *J* = 8.3, 6.8, 1.4 Hz, 0.1H), 7.35 (ddd, *J* = 8.1, 6.7, 1.2 Hz, 0.1H), 7.32 (s, 0.1H), 7.2 (s, 0.9H), 7.17 (dd, *J* = 2.4, 0.7 Hz, 0.9H), 7.1 (dd, *J* = 8.7, 2.4 Hz, 0.9H).

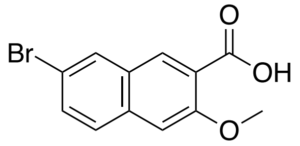

3i

*7-bromo-3-methoxy-2-naphthoic acid (**3i**)*

^{1}H NMR (400 MHz, d_6-DMSO) δ 13.1 (br s, 1H), 8.2 (d, *J* = 2.1 Hz, 1H), 8.2 (s, 1H), 7.8 (d, *J* = 9.3 Hz, 1H), 7.6 (dd, *J* = 8.8, 2.0 Hz, 1H), 7.5 (s, 1H), 3.9 (s, 3H).

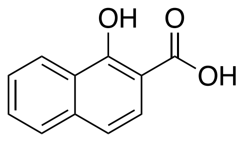

3j

*1-hydroxy-2-naphthoic acid (**3j**)*

^{1}H NMR (400 MHz, d_6-DMSO) δ 13.8 (br s, 1H), 12.7 (br s, 1H), 8.3 (d, J = 8.4 Hz, 1H), 7.9 (d, J = 8.1 Hz, 1H), 7.8 (d, J = 8.7 Hz, 1H), 7.7 (ddd, J = 8.2, 6.9, 1.3 Hz, 1H), 7.6 (ddd, J = 8.2, 6.9, 1.2 Hz, 1H), 7.4 (d, J = 8.4 Hz, 1H).

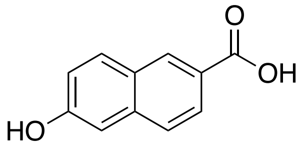

3k

*6-hydroxy-2-naphthoic acid (**3k**)*

^{1}H NMR (400 MHz, d_6-DMSO) δ 12.8 (s, 1H), 10.1 (s, 1H), 8.5 (s, 1H), 7.9 (d, J = 9.2 Hz, 1H), 7.9 (dd, J = 8.6, 1.7 Hz, 1H), 7.7 (d, J = 9.1 Hz, 1H), 7.2 (d, J = 2.5 Hz, 1H), 7.2 (dd, J = 8.7, 2.4 Hz, 1H).

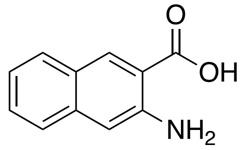

3l

*3-amino-2-naphthoic acid (**3l**)*

^{1}H NMR (400 MHz, d_6-DMSO) δ 8.7 (s, 2H), 8.4 (s, 1H), 7.8 (d, J = 8.2 Hz, 1H), 7.5 (d, J = 8.4 Hz, 1H), 7.4 (ddd, J = 8.2, 6.7, 1.3 Hz, 1H), 7.1 (ddd, J = 8.0, 6.7, 1.2 Hz, 1H), 7.0 (s, 1H); the carboxylic acid proton signal was not detected.

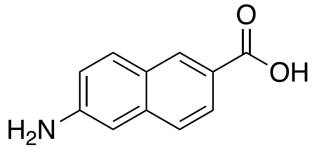

3m

*6-amino-2-naphthoic acid (**3m**)*

^{1}H NMR (400 MHz, d_6-DMSO) δ 12.6 (s, 1H), 8.3 (s, 1H), 7.8 (d, J = 8.6 Hz, 1H), 7.8 (dd, J = 8.6, 1.8 Hz, 1H), 7.6 (d, J = 8.8 Hz, 1H), 7.1 (dd, J = 8.8, 2.2 Hz, 1H), 7.0 (s, 1H), 3.6 (s, 2H).

2. Synthetic procedures and analytical tabulations for synthesized analogs

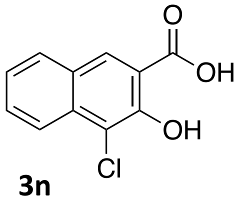

3n

*4-chloro-3-hydroxy-2-naphthoic acid (**3n**)*

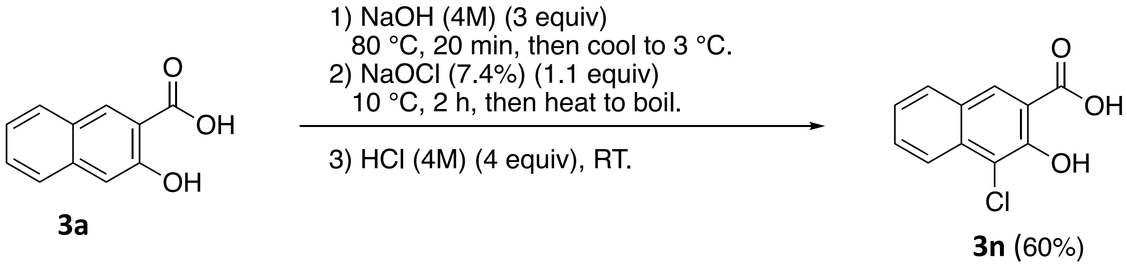

Following the literature procedure[1], 3-hydroxy-2-naphthoic acid (100 mg, 0.53 mmol) was added to a hot (80 °C) 4M sodium hydroxide solution (0.4 mL, 1.6 mmol). The solution was heated for 20 min to dissolve all the 3-hydroxy-2-naphthoic acid and then allowed to cool to 3 °C in an ice bath. A solution of (7.4%) sodium hypochlorite in water (588 mg, 0.58 mmol) was cooled to 3°C then added to the chilled solution of 3-hydroxy-2-naphthoic acid, keeping the temperature below 10 °C. The reaction mixture was held at about 10 °C for 2 hours, heated to the boil and then cooled to ambient temperature. To this solution was then added 4M hydrochloric acid (0.53 mL, 2.1 mmol). The resulting slurry was filtered, washed with water, and dried to constant weight. affording 71 mg of 4-chloro-3-hydroxy-2-naphthoic acid (**3n**) (60% yield).

^{1}H NMR (400 MHz, d_6-DMSO) δ 8.56 (s, 1H), 8.07 (dt, J = 8.2, 0.7 Hz, 1H), 8.04 (dd, J = 8.6, 1.1 Hz, 1H), 7.71 (ddd, J = 8.5, 6.8, 1.3 Hz, 1H), 7.46 (ddd, J = 8.1, 6.8, 1.2 Hz, 1H); the carboxylic acid and hydroxy proton signals were not detected.
^{13}C NMR (101 MHz, d_6-DMSO) δ 171.3, 152.7, 133.7, 131.1, 130.2, 130.1, 126.7, 124.3, 122.1, 116.4, 113.2.

HRMS calculated for $C_{11}H_6ClO_3^-$ 221.0011, found 221.0009

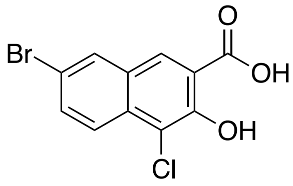

3o

*7-bromo-4-chloro-3-hydroxy-2-naphthoic acid (**3o**)*

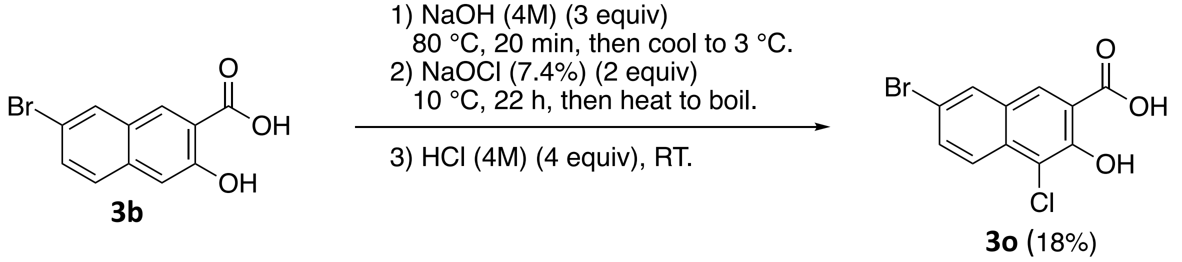

Following the literature procedure[1], 7-bromo-3-hydroxy-2-naphthoic acid (100 mg, 0.374 mmol) was added to a hot (80 °C) 4M sodium hydroxide solution (0.28 mL, 1.1 mmol). The solution was heated for 20 min to dissolve all the 7-bromo-3-hydroxy-2-naphthoic acid and then allowed to cool to 3 °C in an ice bath. A solution of (7.4%) sodium hypochlorite in water (75 mg, 0.74 mmol) was cooled to 3 °C then added to the chilled solution of 7-bromo-3-hydroxy-2-naphthoic acid, keeping the temperature below 10 °C. The reaction mixture is held at about 10°C for 2 hours, heated to the boil and then cooled to ambient temperature. To this solution was then added 4M hydrochloric acid (0.37 mL, 1.5 mmol). The resulting slurry was filtered, washed with water, and dried to constant weight. affording 20 mg of 7-bromo-4-chloro-3-hydroxy-2-naphthoic acid (**3o**) (18% yield).

^{1}H NMR (400 MHz, d_6-DMSO) δ 8.58 (s, 1H), 8.41 (d, J = 2.1 Hz, 1H), 7.99 (dt, J = 9.1, 0.7 Hz, 1H), 7.83 (dd, J = 9.1, 2.1 Hz, 1H); the carboxylic acid and hydroxyl proton signals were not detected.

^{13}C NMR (101 MHz, d_6-DMSO) δ 171.1, 152.6, 133.1, 132.3, 131.6, 130.5, 127.8, 124.5, 117.3, 116.8, 113.7.

HRMS calculated for $C_{11}H_5BrClO_3^-$ 298.9116, found 298.9110

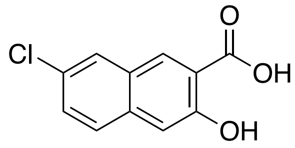

3p

*7-chloro-3-hydroxy-2-naphthoic acid (**3p**)*

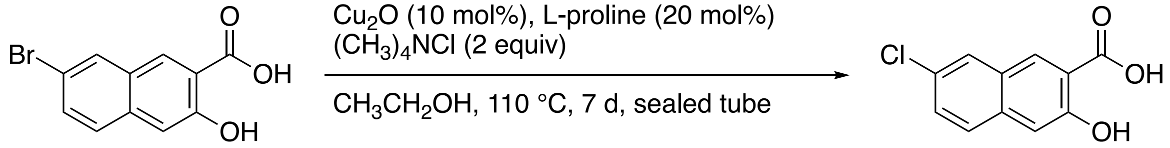

3b **3p**

Following the literature procedure[2], a pressure tube was charged with Cu_2O (16 mg, 10 mol%), L-proline (26 mg, 20 mol%), 7-bromo-3-hydroxy-2-naphthoic acid (300 mg, 1.1mmol), tetramethylammonium chloride (Me4NCl) (246 mg, 2.25 mmol), and EtOH (2.0 mL) under nitrogen atmosphere. The Schlenk tube was sealed with a teflon valve, and then the reaction mixture was stirred at 110 °C for 7 days. After the reaction was completed, the solvent was removed under reduced pressure. The residue obtained was purified via silica gel chromatography (eluent: DCM/MeOH = 98/2) to afford 168 mg of 7-chloro-3-hydroxy-2-naphthoic acid (**3p**) (67% yield).

^{1}H NMR (400 MHz, d_6-DMSO) δ 11.4 (s, 1H), 8.54 (s, 1H), 8.12 (dd, J = 2.2, 0.7 Hz, 1H), 7.82 (dd, J = 8.8, 0.7 Hz, 1H), 7.53 (dd, J = 8.9, 2.2 Hz, 1H), 7.37 (s, 1H); the carboxylic acid proton signal was not detected.
^{13}C NMR (126 MHz, d_6-DMSO) δ 171.3, 156.4, 135.5, 131.8, 129.4, 128.3, 128.2, 128.1, 127.6, 127.2, 111.1.

HRMS calculated for $C_{11}H_6ClO_3^-$ 221.0011, found 221.0028

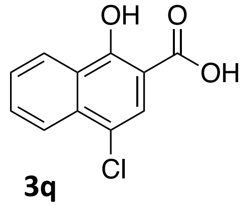

3q

*4-chloro-1-hydroxy-2-naphthoic acid (**3q**)*

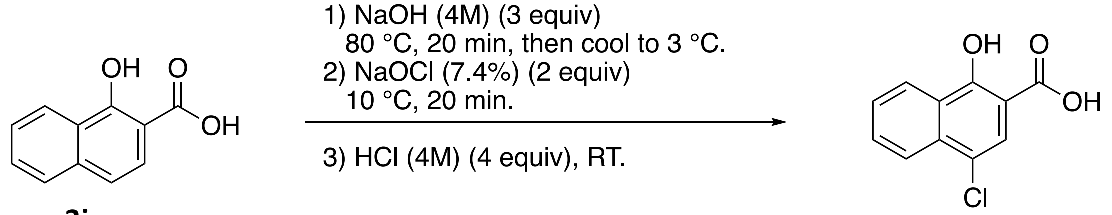

3j **3q** (14%)

Following the literature procedure[1], 1-hydroxy-2-naphthoic acid (1 g, 5.3 mmol) was added to a hot (80 °C) 4M sodium hydroxide solution (4 mL, 16 mmol). The solution was heated for 20 min

to dissolve all the 1-hydroxy-2-naphthoic acid and then allowed to cool to 3 °C in an ice bath. A solution of (7.4%) sodium hypochlorite in water (791 mg, 10.6 mmol) was cooled to 3°C then added to the chilled solution of 1-hydroxy-2-naphthoic acid, keeping the temperature below 10°C. The reaction mixture was held at about 10 °C. for 20 min and then heated to the boil then cooled to ambient temperature. To this solution was then added 4M hydrochloric acid (5.3 mL, 21.3 mmol). The resulting slurry was filtered washed with water and dried to obtain 614 mg of the crude. The residue was recrystallized from DCM affording 160 mg of 4-chloro-1-hydroxy-2-naphthoic acid (**3q**) (14% yield) as white needles.

^{1}H NMR (400 MHz, d_6-DMSO) δ 10.38 (s, 1H), 8.28 (ddd, J = 8.1, 1.5, 0.7 Hz, 1H), 8.09 (ddd, J = 8.3, 1.5, 0.7 Hz, 1H), 7.71 (ddd, J = 8.3, 6.8, 1.6 Hz, 1H), 7.70 (s, 1H), 7.66 (ddd, J = 8.2, 6.9, 1.4 Hz, 1H); the carboxylic acid proton signal is not detected.
^{13}C NMR (101 MHz, d_6-DMSO) δ 174.9, 148.0, 129.5, 128.0, 127.0, 126.8, 126.6, 123.8, 122.8, 121.2, 113.9.

HRMS calculated for $C_{11}H_8ClO_3^+$ 223.0156, found 223.0162

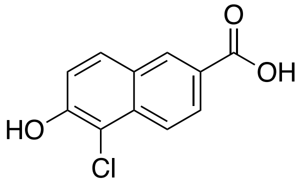

3r

*5-chloro-6-hydroxy-2-naphthoic acid (**3r**)*

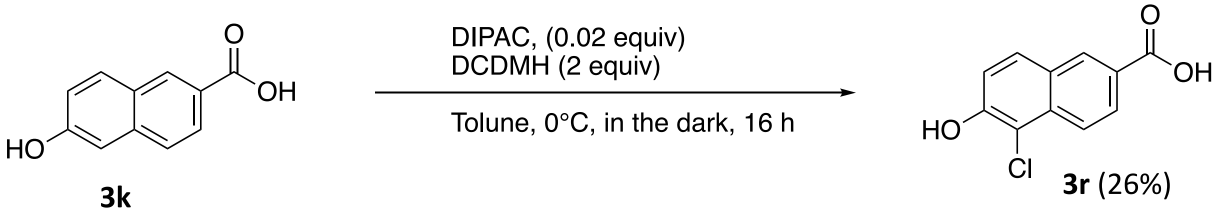

Following the literature procedure[3], to a solution of 6-hydroxy-2-naphthoic acid (100 mg, 0.53 mmol), diisopropylammonium chloride (DIPAC) (1.5 mg, 0.01 mmol) in toluene (2 mL) was added 1,3-dichloro-5,5-dimethylimidazolidine-2,4-diimine (DCDMH) (207 mg, 1.1 mmol) at 0 °C in the absence of light. The mixture was stirred at 0 °C for 16 h and quenched by saturated aqueous Na_2SO_3 (3 mL). The solution was diluted with water (5 mL) and extracted with EtOAc. The combined organic extracts were washed with brine, dried with anhydrous Na_2SO_4, filtered, and concentrated under vacuum. The residue was recrystallized from MeOH to give 30 mg of 5-chloro-6-hydroxy-2-naphthoic acid (**3r**) (26% yield) as yellow needles.

^{1}H NMR (400 MHz, d_6-DMSO) δ 12.98 (s, 1H), 10.88 (s, 1H), 8.54 (d, J = 1.7 Hz, 1H), 8.08 (d, J = 8.9 Hz, 1H), 8.04 (dd, J = 8.9, 1.7 Hz, 1H), 7.99 (d, J = 8.7 Hz, 1H), 7.37 (d, J = 8.9 Hz, 1H).
^{13}C NMR (101 MHz, d_6-DMSO) δ 167.2, 153.2, 133.6, 131.0, 129.7, 127.4, 126.9, 125.6, 122.5, 119.2, 112.4.

HRMS calculated for $C_{11}H_6ClO_3^-$ 221.0011, found 221.0005

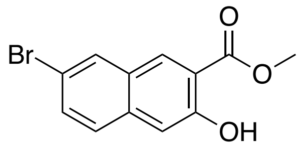

4b

*methyl 7-bromo-3-hydroxy-2-naphthoate (**4b**)*

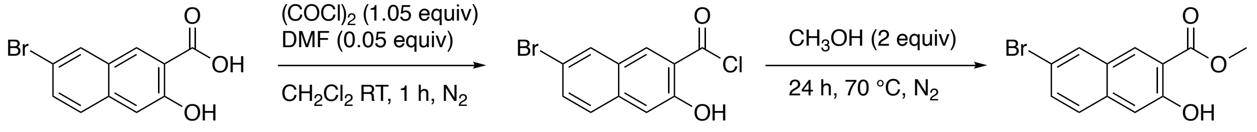

3b **4b** (76%)

A 25 mL RBF was charged with 7-bromo-3-hydroxy-2-naphthoic acid (**3b**) (500 mg, 1.87 mmol), and purged with nitrogen. Then anhydrous CH_2Cl_2 (5 mL) was added as solvent. Oxalyl chloride (0.18 mL, 2.06 mmol) was added. Then a drop of DMF was added, and the resulting mixture was stirred for 1 h at room temperature. Then the mixture was concentrated in vacuo. The acid chloride was dissolved in (5 mL) MeOH under nitrogen atmosphere in a sealed tube and stirred at 70 °C for 24 h. The residue was purified by column chromatography on silica gel to obtained 400 mg of methyl 7-bromo-3-hydroxy-2-naphthoate (**4b**) (76% yield).

^{1}H NMR (400 MHz, $CDCl_3$) δ 10.46 (s, 1H), 8.40 (s, 1H), 7.96 (s, 1H), 7.61 – 7.51 (m, 2H), 7.27 (d, J = 11.3 Hz, 1H), 4.04 (s, 3H).
^{13}C NMR (101 MHz, $CDCl_3$) δ 169.9, 156.6, 136.1, 132.3, 131.3, 130.8, 127.97, 127.92, 117.3, 114.9, 111.9, 52.7.

HRMS calculated for $C_{12}H_{10}BrO_3^{+}$ 280.9808, found 280.9805

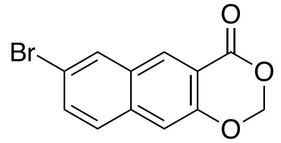

5b

*7-bromo-4H-naphtho[2,3-d][1,3]dioxin-4-one (**5b**)*

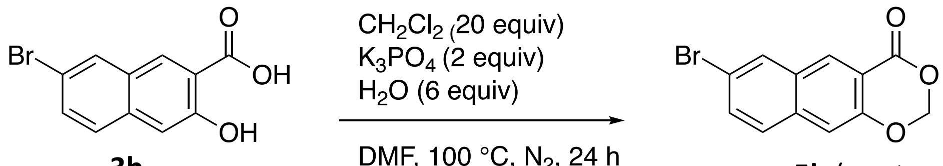

3b **5b** (77%)

Following a related literature procedure[4], a pressure tube was charged with 7-bromo-3-hydroxy-2-naphthoic acid (**3b**) (50 mg, 0.19 mmol), K3PO4 (80 mg, 0.37 mmol), DCM (0.24 mL, 3.7 mmol, H_2O (0.02 mL, 1.1 mmol) and DMF (2 mL) and stirred at 100 °C under nitrogen atmosphere for 24 hours. After cooling to room temperature, the reaction solution was extracted with EtOAc and the resulting solution was washed with saturated $NaHCO_3$ solution, water and brine. Then the organic layer was dried over anhydrous $MgSO_4$, and purified by silica gel chromatography (eluent: DCM/MeOH = 95/5) to afford 40 mg of 7-bromo-4*H*-naphtho[2,3-*d*][1,3]dioxin-4-one (**5b**) (77% yield).

^1H NMR (400 MHz, d_6-DMSO) δ 8.70 (s, 1H), 8.46 (d, J = 1.4 Hz, 1H), 7.91 (d, J = 8.2 Hz, 1H), 7.78 (dd, J = 8.9, 2.0 Hz, 1H), 7.72 (s, 1H), 5.91 (s, 2H).
^{13}C NMR (101 MHz, d_6-DMSO) δ 161.2, 153.5, 135.3, 132.6, 131.6, 131.2, 130.1, 129.2, 118.6, 116.2, 112.4, 91.5.

HRMS calculated for $C_{12}H_8BrO_3^+$278.9651, found 278.9641.

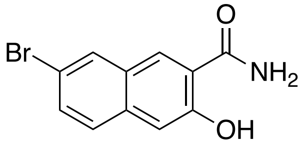

6b

*7-bromo-3-hydroxy-2-naphthamide (**6b**)*

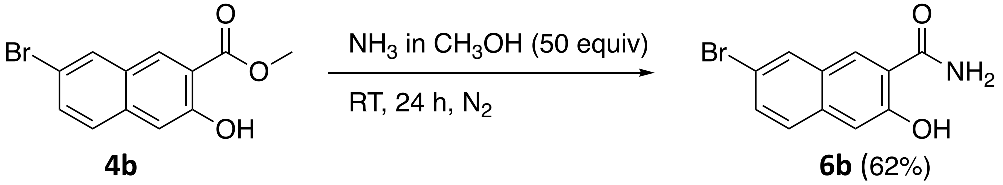

To a 10 mL RBF were added **4b** (50 mg, 0.18 mmol), and dissolved in (2 mL) ammonia (15 w% in MeOH, 151 mg) under nitrogen atmosphere and stirred at room temperature for 24h. Then DCM (0.5 mL) was added, and the product (**6b**) precipitated as white solid. The precipitate was filtered and dried to obtain 29 mg of 7-bromo-3-hydroxy-2-naphthamide (**6b**) (62% yield).

^1H NMR (400 MHz, d_6-DMSO) δ 12.39 (s, 1H), 8.56 (s, 1H), 8.48 (s, 1H), 8.07 (s, 1H), 8.02 (d, J = 2.0 Hz, 1H), 7.72 (d, J = 8.8 Hz, 1H), 7.60 (dd, J = 8.9, 2.0 Hz, 1H), 7.29 (s, 1H).
^{13}C NMR (101 MHz, d_6-DMSO) δ 170.6, 156.5, 134.7, 131.1, 130.2, 129.2, 128.1, 127.5, 118.9, 116.1, 110.9.

HRMS calculated for $C_{11}H_7BrNO_2^-$ 263.9666, found 263.9672.

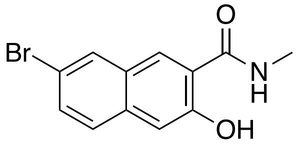

7b

*7-bromo-3-hydroxy-N-methyl-2-naphthamide (7**b**)*

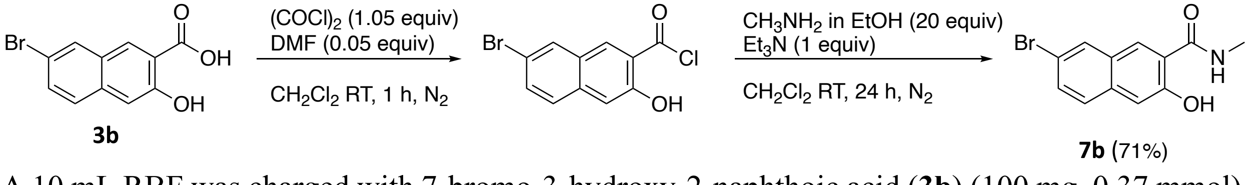

A 10 mL RBF was charged with 7-bromo-3-hydroxy-2-naphthoic acid (**3b**) (100 mg, 0.37 mmol), and purged with nitrogen. Then anhydrous CH_2Cl_2 (2 mL) was added as solvent. Oxalyl chloride (0.04 mL, 0.41 mmol) was added. Then a drop of DMF was added, and the resulting mixture was stirred for 1 h at room temperature. Then the mixture was concentrated in vacuo. The acid chloride

was dissolved in methyl amine (33 w% in EtOH, 659 mg, 7 mmol), and triethylamine (0.05 mL, 35 mg, 0.37 mmol) was added under nitrogen atmosphere and stirred at room temperature for 24 h. The mixture was diluted with CH_2Cl_2 (3 mL) and H_2O (5 mL) and extracted with CH_2Cl_2 (3 x 5 mL). The combined organic extracts were washed with brine, dried with sodium sulfate, concentrated in vacuo, and purified by silica gel chromatography (eluent: Hexanes/EtOAc = 6/4) to afford 70 mg of 7-bromo-3-hydroxy-*N*-methyl-2-naphthamide (**7b**) (71% yield).

H NMR (400 MHz, d_6-DMSO) δ 11.99 (s, 1H), 8.92 (d, J = 4.9 Hz, 1H), 8.38 (s, 1H), 8.05 (d, J = 2.7 Hz, 1H), 7.71 (d, J = 9.4 Hz, 1H), 7.57 (dd, J = 8.8, 2.0 Hz, 1H), 7.29 (s, 1H), 2.85 (d, J = 4.6 Hz, 3H).
^{13}C NMR (101 MHz, d_6-DMSO) δ 167.9, 155.6, 134.5, 130.8, 130.1, 128.6, 128.1, 127.7, 120.1, 116.1, 110.9, 26.2.
HRMS calculated for $C_{12}H_{11}BrNO_2^+$ 279.9968, found 279.9959

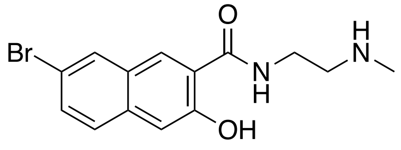

8b

*7-bromo-3-hydroxy-N-(2-(methylamino)ethyl)-2-naphthamide (**8b**)*

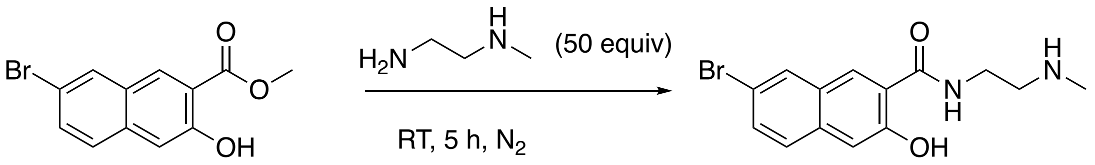

4b

8b (89%)

To a 10 mL RBF were added **4b** (40 mg, 0.14 mmol), and dissolved in N^1-methylethane-1,2-diamine (0.25 mL, 2.8 mmol), under nitrogen atmosphere and stirred at room temperature for 5h. the reaction solution was extracted with EtOAc and the resulting solution was washed with saturated $NaHCO_3$ solution, water and brine. Then the organic layer was dried over anhydrous $MgSO_4$ and concentrated in vacuo to give 40 mg of **8b** (89% yield).

^{1}H NMR (400 MHz, d_6-DMSO) δ 9.92 (s, 1H), 8.41 (s, 1H), 8.01 (s, 1H), 7.57 (d, J = 8.9 Hz, 1H), 7.46 (dd, J = 8.8, 2.0 Hz, 1H), 7.16 (s, 1H), 3.48 (t, J = 6.2 Hz, 2H), 2.78 (t, J = 6.2 Hz, 2H), 2.37 (s, 3H).
^{13}C NMR (101 MHz, d_6-DMSO) δ 167.1, 158.6, 134.7, 130.2, 130.1, 129.4, 127.9, 126.7, 122.1, 114.6, 111.0, 50.1, 38.1, 35.2.
HRMS calculated for $C_{14}H_{16}BrN_2O_2^+$ 323.0390, found 323.0394.

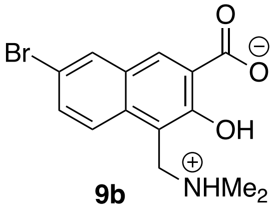

9b

*7-bromo-4-((dimethylamino)methyl)-3-hydroxy-2-naphthoic acid (**9b**)*

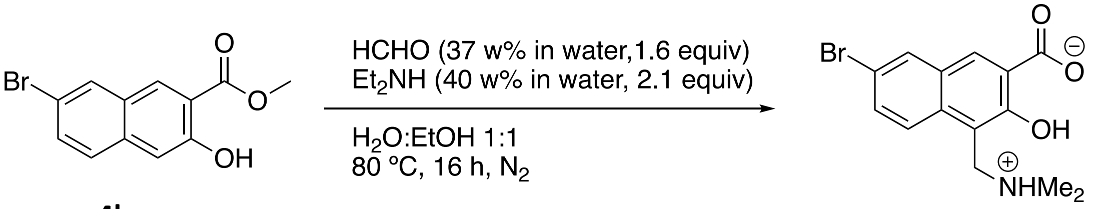

4b **9b** (46%)

Following a related literature procedure[5], to a 10 mL RBF were added **4b** (100 mg, 0.36 mmol), water (1 mL), EtOH (1 mL), formalin (37 w% in water, 46 mg), and diethylamine (40 w% in water 84 mg). The mixture was heated to 80 ºC for 16 h. After cooling to room temperature, crystals formed, and were collected by filitration, affording 55 mg of methyl 7-bromo-4-((dimethylamino)methyl)-3-hydroxy-2-naphthoic acid (**9b**) (46% yield) as yellow cubes. Note that under these basic Mannich reaction conditions, hydrolysis of the methyl ester occurred.

^{1}H NMR (400 MHz, d_6-DMSO) δ 9.1 (s, 1H), 8.4 (s, 1H), 8.2 (d, J = 2.2 Hz, 1H), 7.9 (d, J = 9.2 Hz, 1H), 7.6 (dd, J = 9.1, 2.2 Hz, 1H), 4.6 (s, 2H), 2.8 (s, 6H).
^{13}C NMR (101 MHz, d_6-DMSO) δ 169.4, 162.9, 133.9, 131.6, 131.4, 130.4, 126.5, 123.6, 122.5, 114.1, 107.6, 51.8, 42.4.

HRMS calculated for $C_{14}H_{15}BrNO_3^+$ 324.0230, found 324.0240

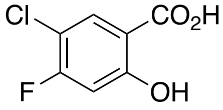

1m

*5-chloro-4-fluoro-2-hydroxybenzoic acid (**1m**)*

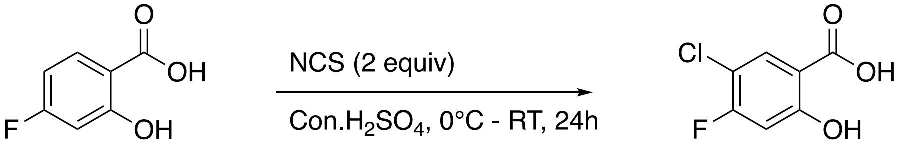

1f **1m** (42%)

Following a related literature procedure[6], 4-fluoro-2-hydroxybenzoic acid (**1f**) (100 mg, 0.64 mmol), was dissolved in concentrated H_2SO_4 (2 mL) and placed in an ice bath. *N*-Chlorosuccinimide (171 mg, 1.28 mmol), was slowly added in small portions, at which point the ice bath was removed. The reaction mixture was stirred at room temperature for 24h. After complete consumption of starting material, water was carefully added. The formed precipitate was filtered and washed with cold water to give 90 mg of the crude product. The residue was recrystallized from EtOAc/DCM to give 50 mg of 4-fluoro-2-hydroxybenzoic acid (**1m**) (42% yield) as colorless crystals.

^{1}H NMR (400 MHz, d_6-DMSO) δ 7.9 (d, $J = 8.7$ Hz, 1H), 7.1 (d, $J = 10.9$ Hz, 1H); the carboxylic acid and hydroxyl protons were not detected.
^{13}C NMR (126 MHz, d_6-DMSO) 170.3, 161.7 (d, $^{3}J = 13.1$ Hz), 161.0 (d, $^{1}J = 253.0$ Hz), 131.8 (d, $^{4}J = 2.3$ Hz), 111.5 (d, $^{3}J = 2.7$ Hz), 110.0 (d, $^{2}J = 18.7$ Hz), 105.9 (d, $^{2}J = 23.8$ Hz).
^{19}F NMR (376 MHz, d_6-DMSO) δ -105.9 (dd, $J = 10.9$, 8.4 Hz).
HRMS calculated for $C_7H_5ClFO_3^+$ 190.9906, found 190.9900

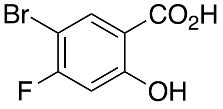

1n

*5-bromo-4-fluoro-2-hydroxybenzoic acid (**1n**)*

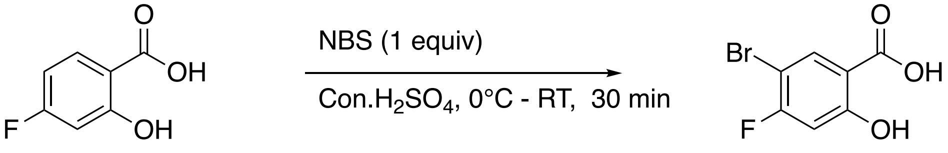

1f **1n** (22%)

Following the related literature procedure[6], 4-fluoro-2-hydroxybenzoic acid (**1f**) (100 mg, 0.64 mmol), was dissolved in concentrated H_2SO_4 (2 mL). *N*-bromosuccinimide (114 mg, 0.64 mmol), was slowly added in small portions while keeping the temperature between 0 and 5 °C during the addition. The reaction mixture was stirred at room temperature for 30 min. After complete consumption of starting material, water was carefully added. The formed precipitate was filtered and washed with cold water to give 123 mg of the crude product. The residue was recrystallized from EtOAc/DCM to give 32 mg 5-bromo-4-fluoro-2-hydroxybenzoic acid (**1n**) (22% yield) as colorless crystals.

^{1}H NMR (400 MHz, d_6-DMSO) δ 8.0 (d, $J = 8.2$ Hz, 1H), 7.1 (d, $J = 10.4$ Hz, 1H); the carboxylic acid and hydroxy proton signals were not detected.
^{13}C NMR (126 MHz, d_6-DMSO) δ 170.1, 162.2 (d, $^{3}J = 13.2$ Hz), 162.0 (d, $^{1}J = 251.1$ Hz), 134.6, 112.0, 105.6 (d, $^{2}J = 25.1$ Hz), 97.3 (d, $^{2}J = 22.1$ Hz).
^{19}F NMR (376 MHz, d_6-DMSO) δ -98.0 (dd, $J = 10.2$, 8.2 Hz).
HRMS calculated for $C_7H_5BrFO_3^+$ 234.9401, found 234.9400

3. References

1. Ruud, J. United States Patent : 3871965 United States Patent : 3871965. *Yeast* **2**, 4–6 (2010).
2. Feng, X. *et al.* Copper-catalyzed conversion of aryl and heteroaryl bromides into the corresponding chlorides. *Chem. Commun.* **48**, 9468–9470 (2012).
3. Xiong, X. & Yeung, Y. Y. Ammonium Salt-Catalyzed Highly Practical Ortho-Selective Monohalogenation and Phenylselenation of Phenols: Scope and Applications. *ACS Catal.* **8**, 4033–4043 (2018).

4. Lin, F., Song, Q., Gao, Y. & Cui, X. A catalyst-free, facile and efficient approach to cyclic esters: Synthesis of 4H-benzo[d][1,3]dioxin-4-ones. *RSC Adv.* **4**, 19856–19860 (2014).
5. Ding, S., Fike, K. R., Klemba, M. & Carlier, P. R. In vitro and in vivo evaluation of the antimalarial MMV665831 and structural analogs. *Bioorganic Med. Chem. Lett.* **30**, 127348 (2020).
6. Paraskevopoulos, G. *et al.* Novel salicylanilides from 4,5-dihalogenated salicylic acids: Synthesis, antimicrobial activity and cytotoxicity. *Bioorganic Med. Chem.* **25**, 1524–1532 (2017).

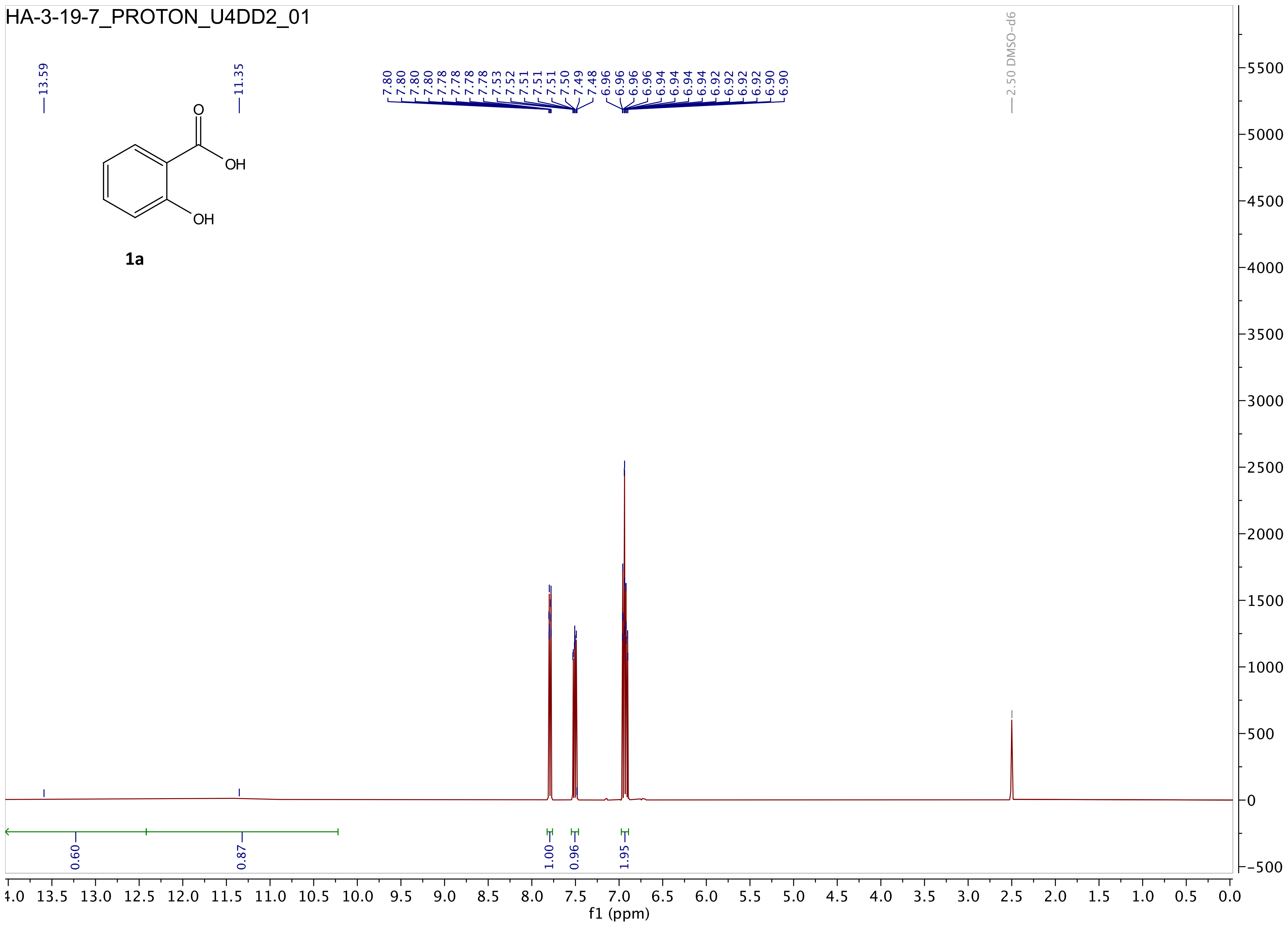
HA-3-19-7_PROTON_U4DD2_01
13.59
11.35
7.80 7.80 7.80 7.80 7.78 7.78 7.78 7.78 7.53 7.52 7.51 7.51 7.51 7.50 7.49 7.48 6.96 6.96 6.96 6.96 6.94 6.94 6.94 6.94 6.92 6.92 6.92 6.92 6.90 6.90
2.50 DMSO-d6
O
OH
OH
1a
0.60
0.87
1.00
0.96
1.95
f1 (ppm)

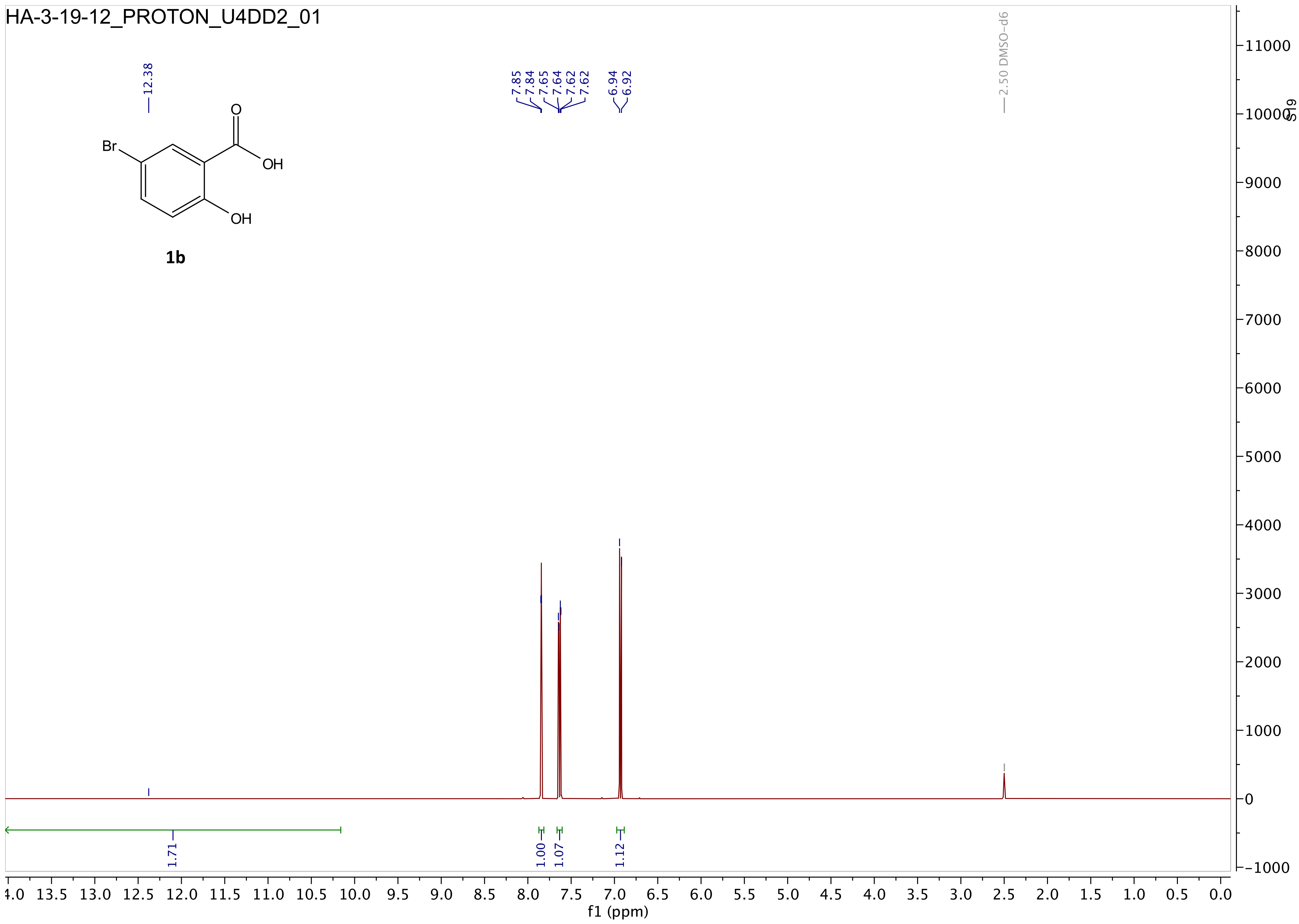

HA-3-19-12_PROTON_U4DD2_01
12.38
7.85
7.84
7.65
7.64
7.62
7.62
6.94
6.92
2.50 DMSO-d6
Br
O
OH
OH
1b
1.71
1.00
1.07
1.12
f1 (ppm)

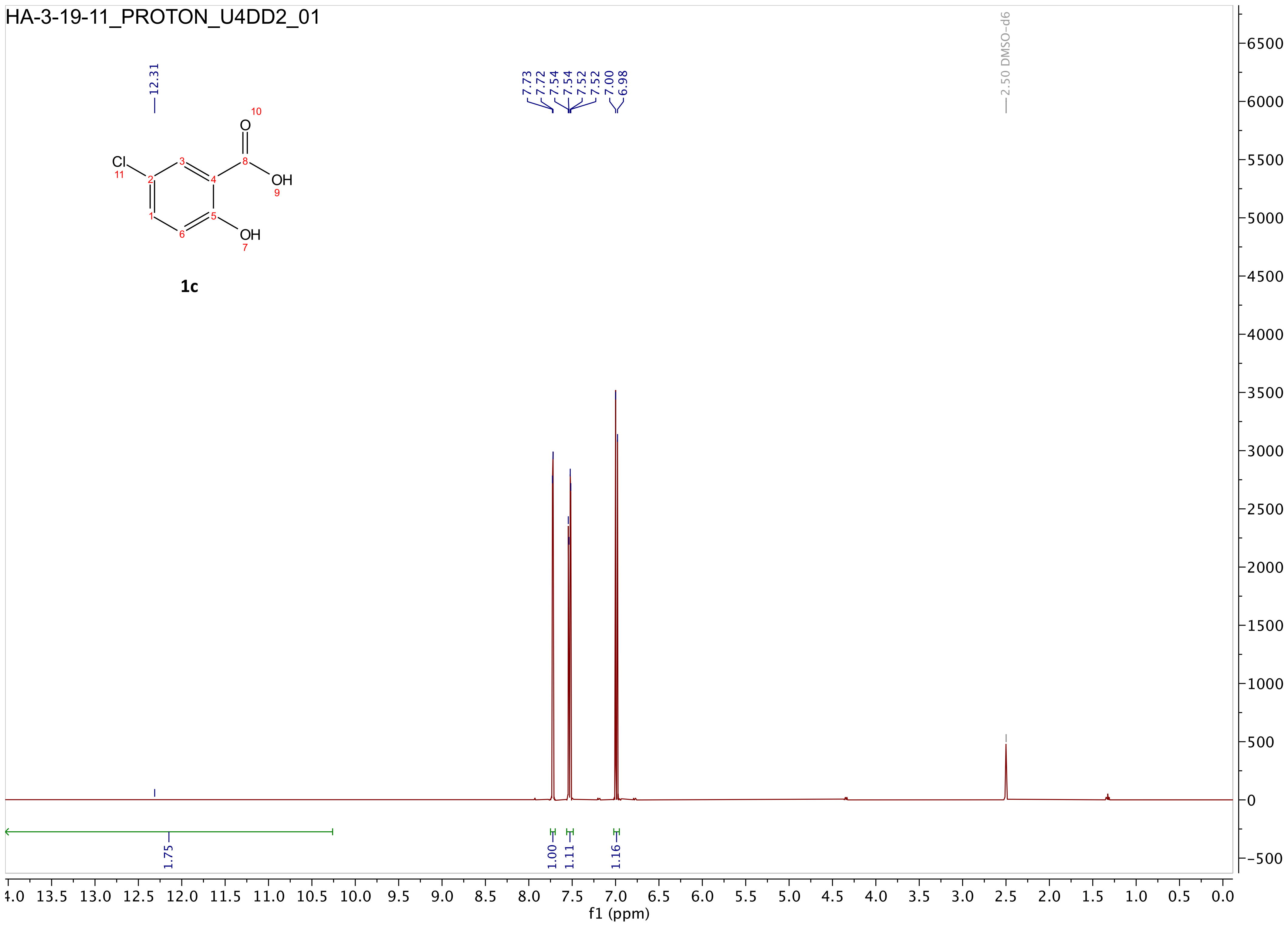
HA-3-19-11_PROTON_U4DD2_01
12.31
7.73
7.72
7.54
7.54
7.52
7.52
7.00
6.98
2.50 DMSO-d6
Cl
OH
O
OH
1c
1.75
1.00
1.11
1.16
f1 (ppm)

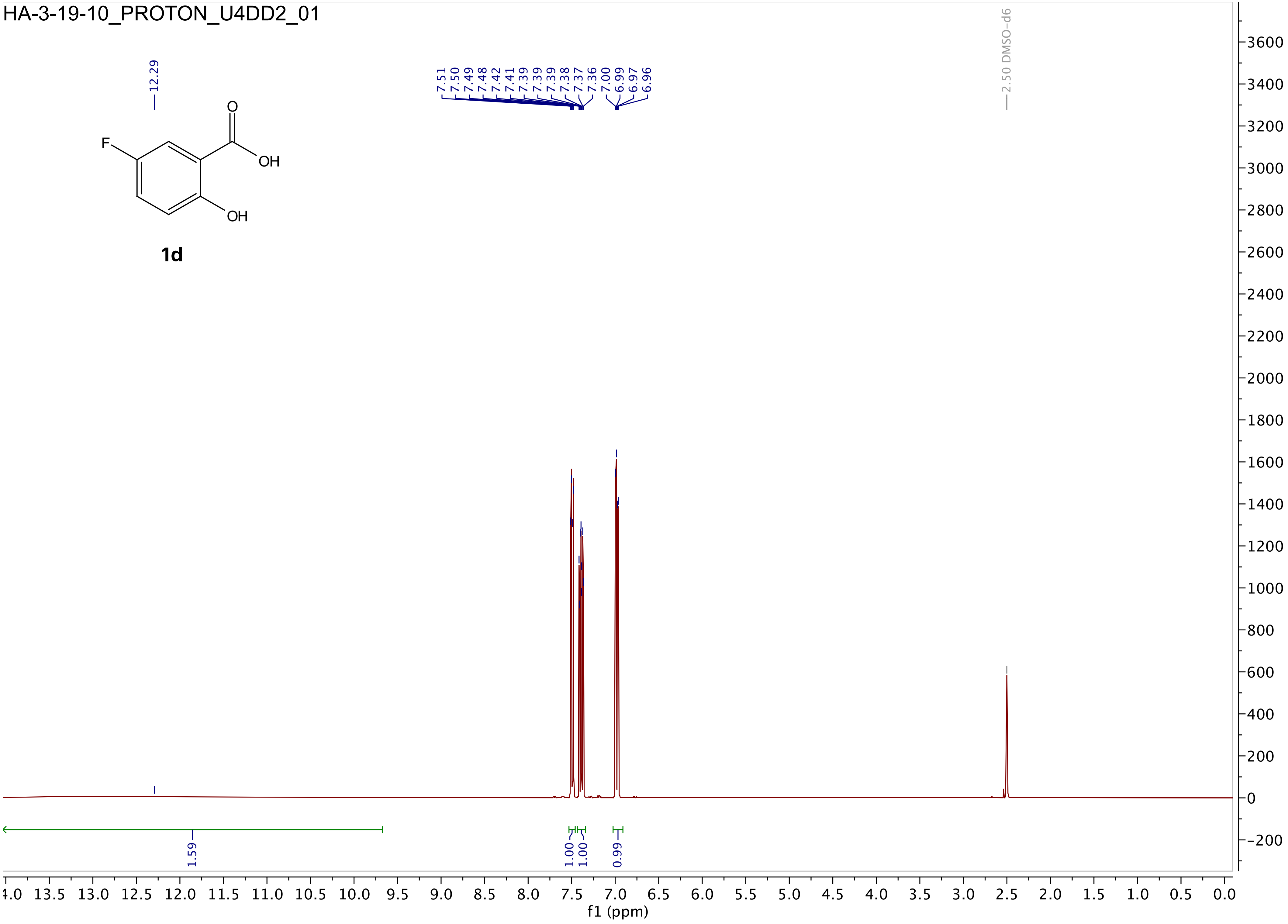
HA-3-19-10_PROTON_U4DD2_01
12.29
7.51
7.50
7.49
7.48
7.42
7.41
7.39
7.39
7.39
7.38
7.37
7.36
7.00
6.99
6.97
6.96
2.50 DMSO-d6
F
O
OH
OH
1.59
1.00
1.00
0.99
f1 (ppm)

1d

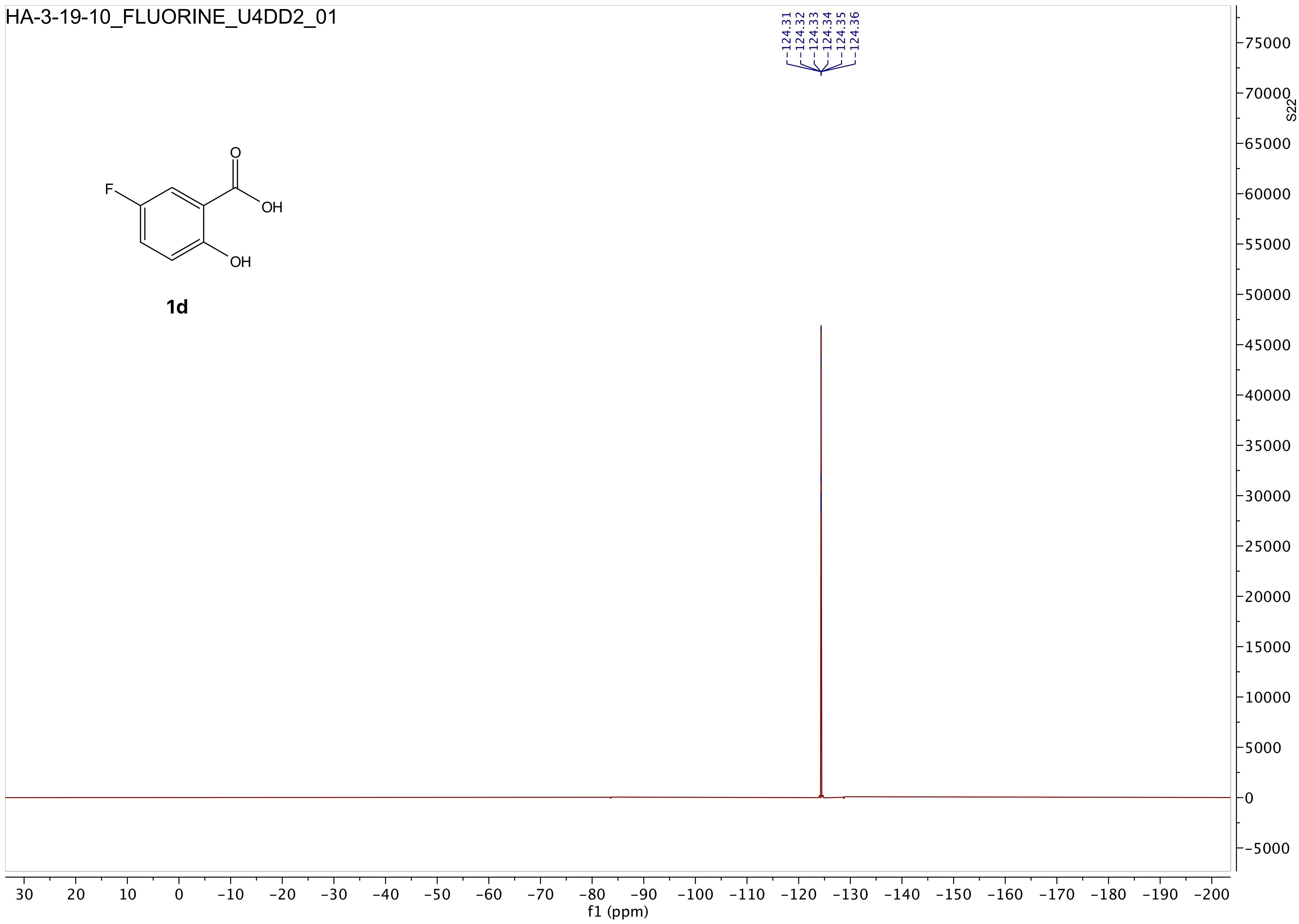

HA-3-19-10_FLUORINE_U4DD2_01
F
O
OH
OH
1d
-124.31
-124.32
-124.33
-124.34
-124.35
-124.36
30 20 10 0 −10 −20 −30 −40 −50 −60 −70 −80 −90 −100 −110 −120 −130 −140 −150 −160 −170 −180 −190 −200
f1 (ppm)
75000 70000 65000 60000 55000 50000 45000 40000 35000 30000 25000 20000 15000 10000 5000 0 −5000

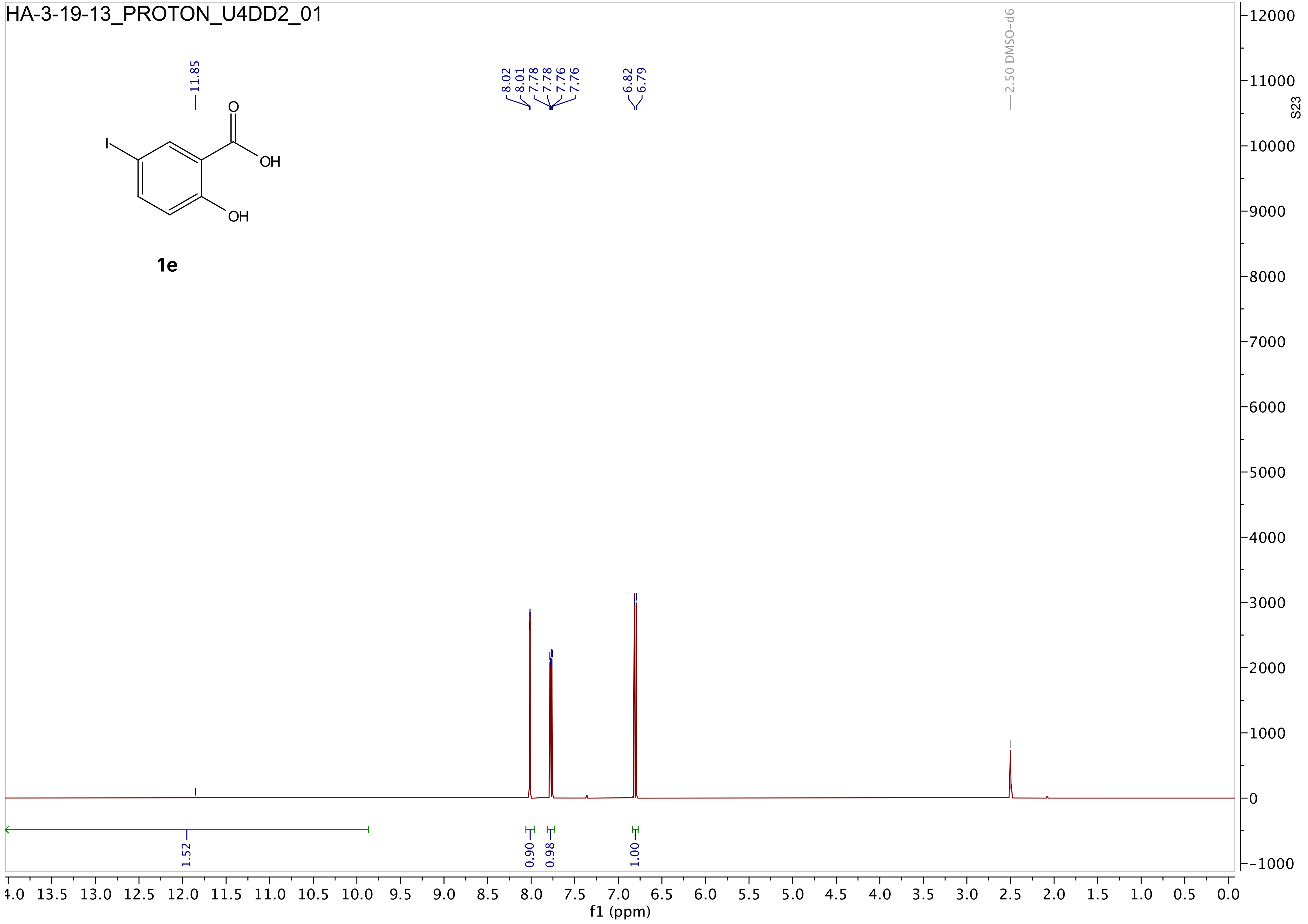
HA-3-19-13_PROTON_U4DD2_01
11.85
8.02
8.01
7.78
7.78
7.76
7.76
6.82
6.79
2.50 DMSO-d6
1.52
0.90
0.98
1.00
f1 (ppm)

1e

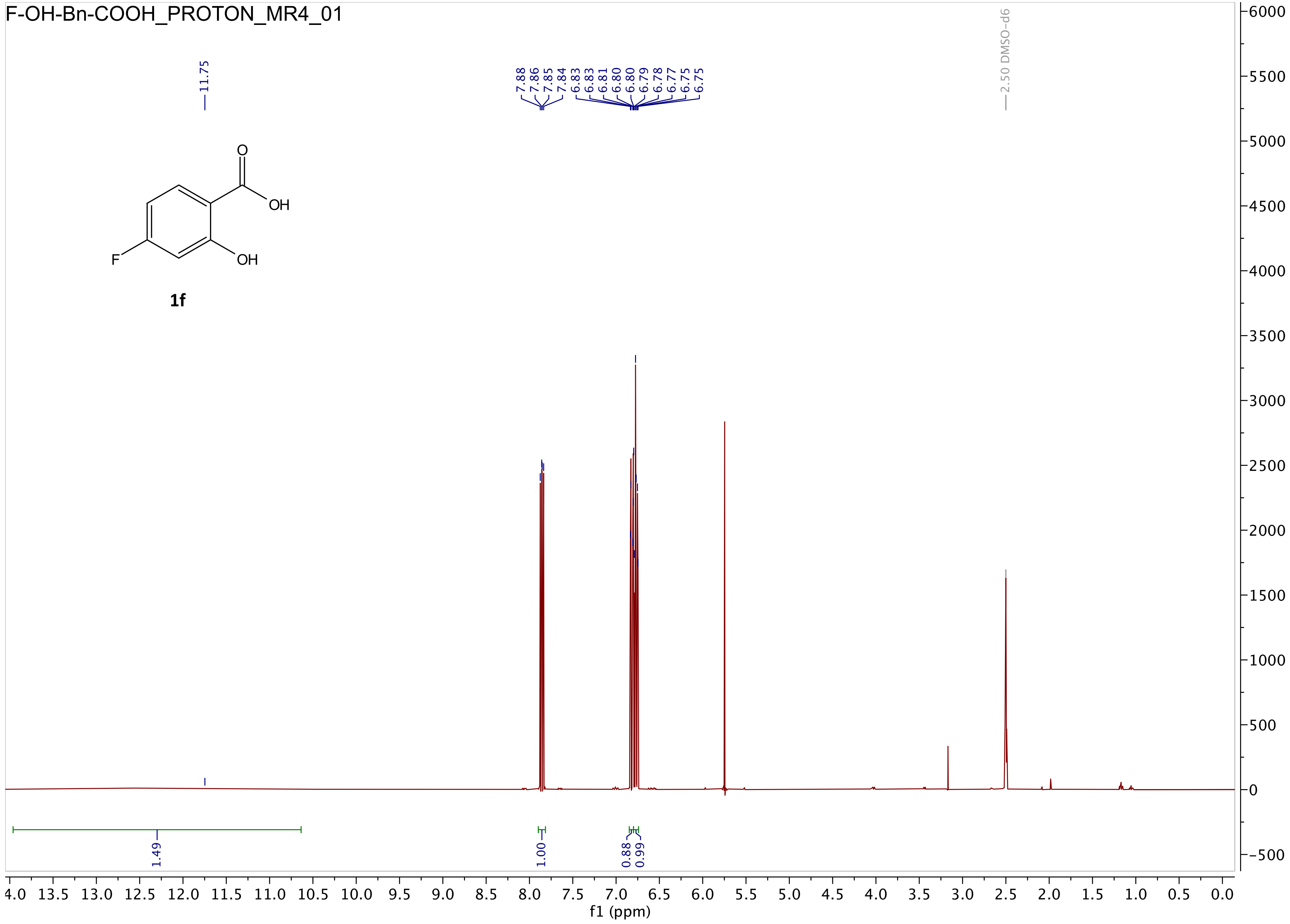
F-OH-Bn-COOH_PROTON_MR4_01
11.75
7.88
7.86
7.85
7.84
6.83
6.83
6.81
6.80
6.80
6.79
6.78
6.77
6.75
6.75
2.50 DMSO-d6
O
OH
F
OH
1f
1.49
1.00
0.88
0.99
f1 (ppm)

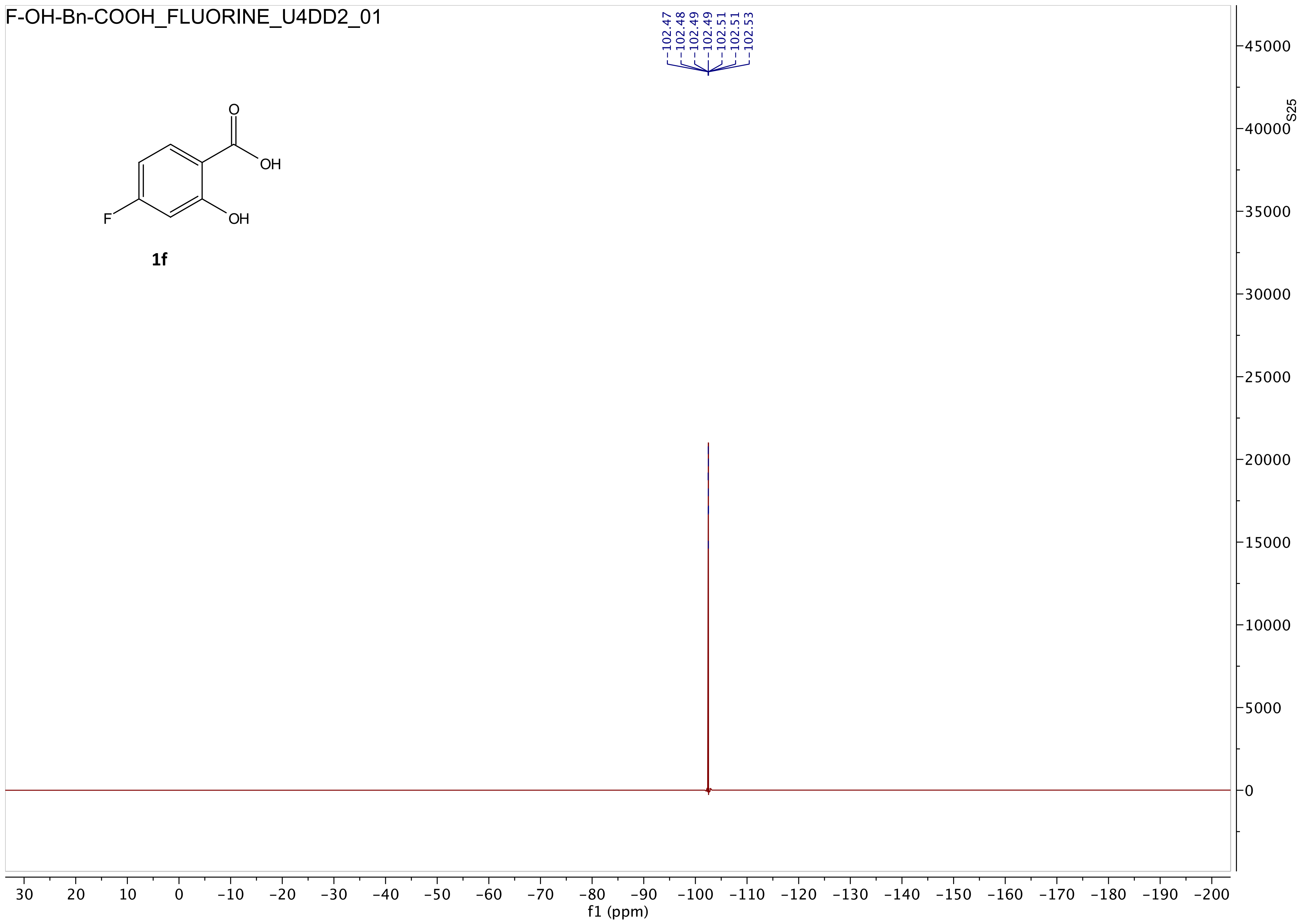
F-OH-Bn-COOH_FLUORINE_U4DD2_01
−102.47
−102.48
−102.49
−102.49
−102.51
−102.51
−102.53
O
OH
F
OH
1f
45000
40000
35000
30000
25000
20000
15000
10000
5000
0
30 20 10 0 −10 −20 −30 −40 −50 −60 −70 −80 −90 −100 −110 −120 −130 −140 −150 −160 −170 −180 −190 −200
f1 (ppm)

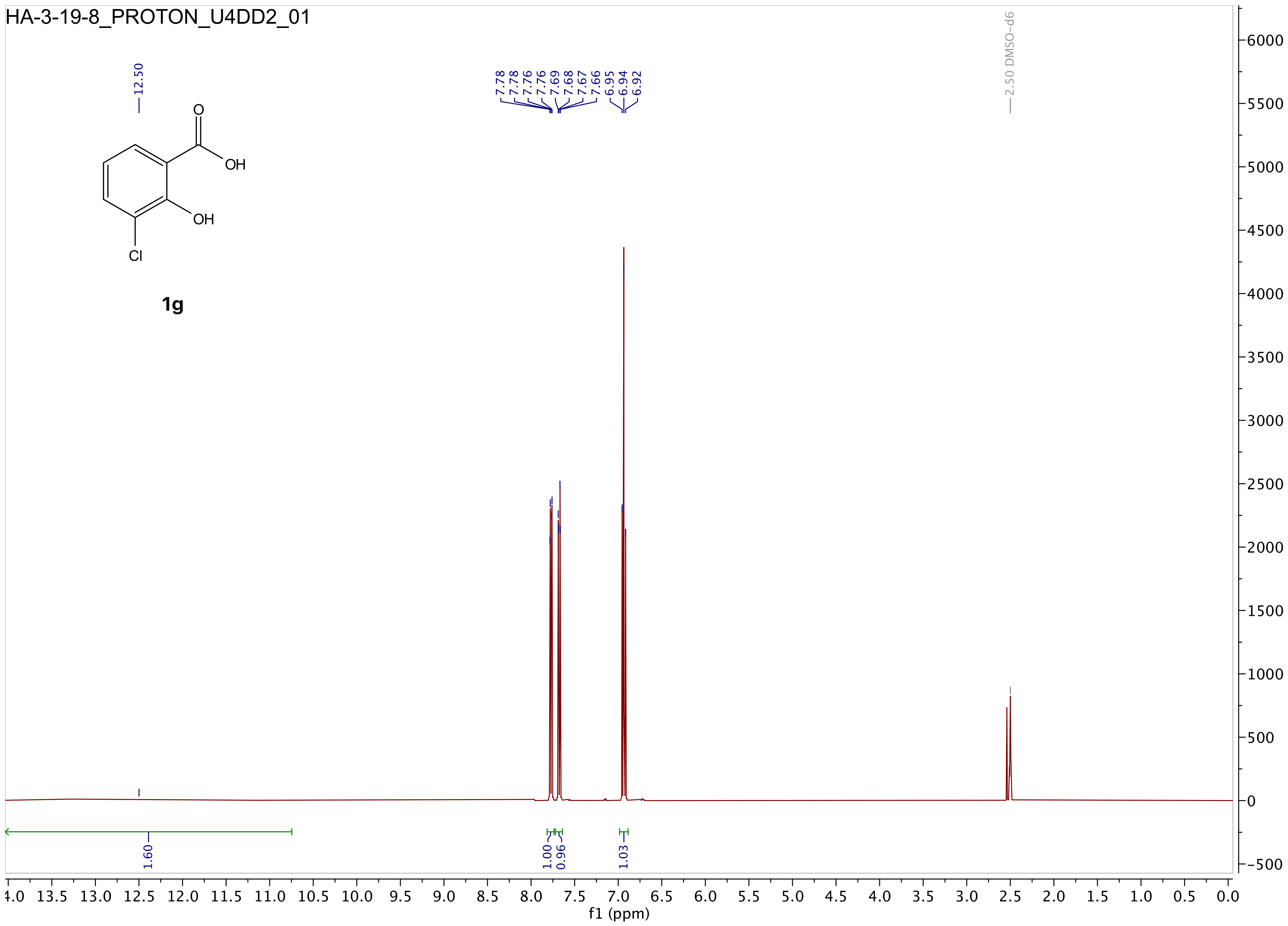
HA-3-19-8_PROTON_U4DD2_01
12.50
O
OH
OH
Cl
1g
7.78
7.78
7.76
7.76
7.69
7.68
7.67
7.66
6.95
6.94
6.92
2.50 DMSO-d6
1.60
1.00
0.96
1.03
f1 (ppm)

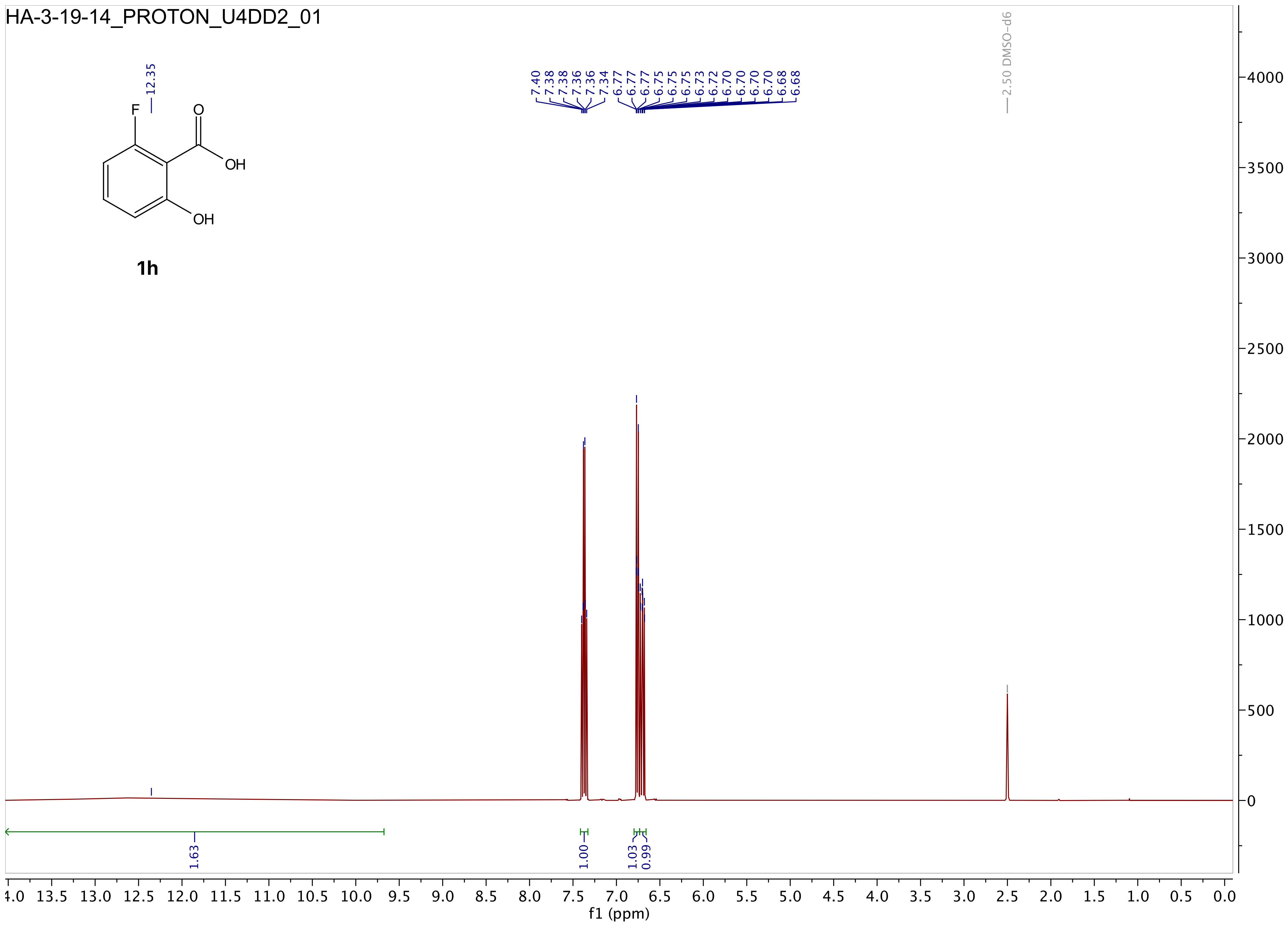
HA-3-19-14_PROTON_U4DD2_01
F
O
OH
OH
12.35
7.40 7.38 7.38 7.36 7.36 7.34 6.77 6.77 6.77 6.75 6.75 6.75 6.73 6.72 6.70 6.70 6.70 6.70 6.68 6.68
2.50 DMSO-d6
1.63
1.00
1.03
0.99
f1 (ppm)
14.0 13.5 13.0 12.5 12.0 11.5 11.0 10.5 10.0 9.5 9.0 8.5 8.0 7.5 7.0 6.5 6.0 5.5 5.0 4.5 4.0 3.5 3.0 2.5 2.0 1.5 1.0 0.5 0.0
4000 3500 3000 2500 2000 1500 1000 500 0
1h

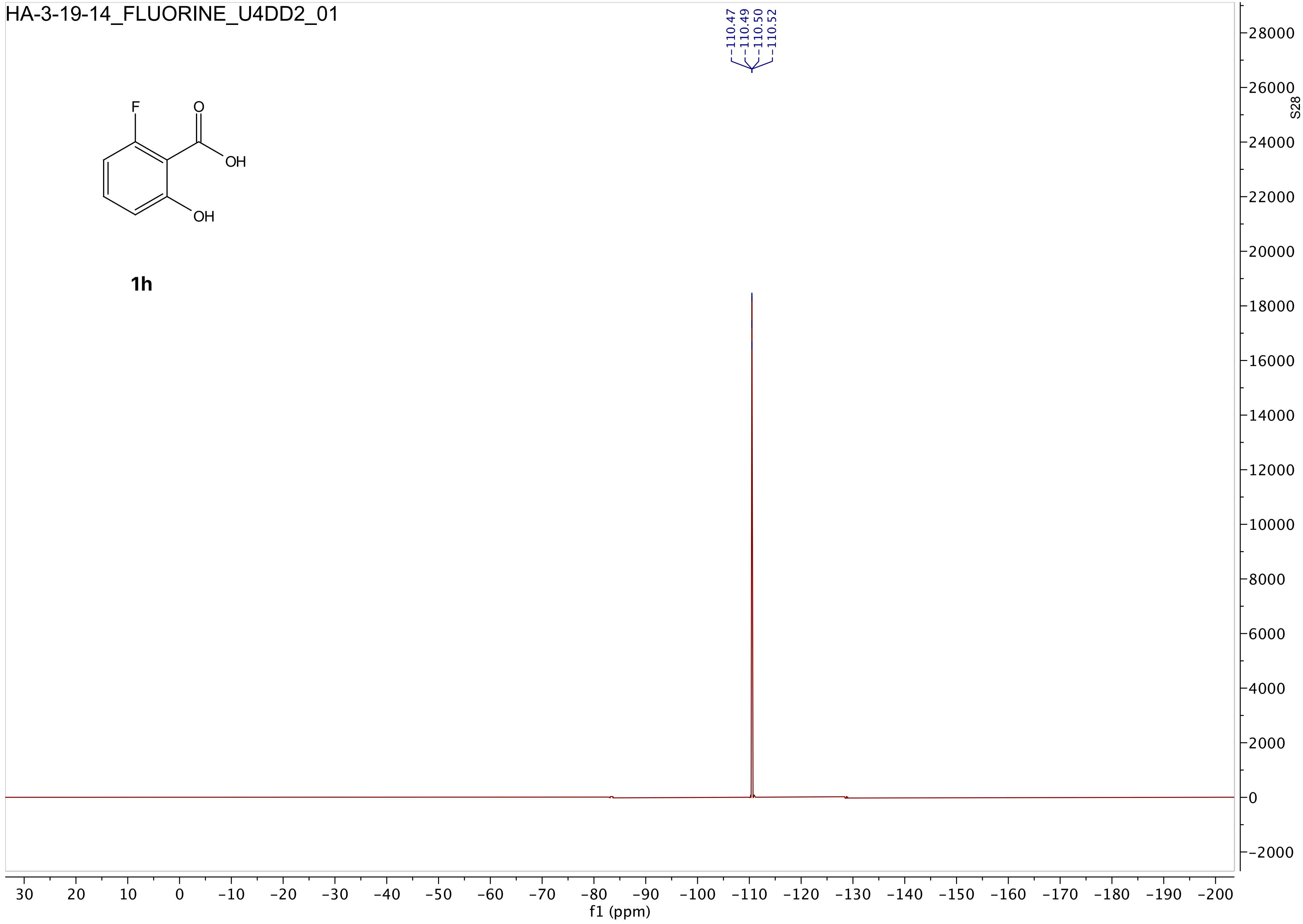
HA-3-19-14_FLUORINE_U4DD2_01
F
O
OH
OH
1h
-110.47
-110.49
-110.50
-110.52
f1 (ppm)

HA-3-19-1.1.fid
PROTON
PROTONRO_ICON DMSO /opt/topspin3.2/icon-data carlier-4 8

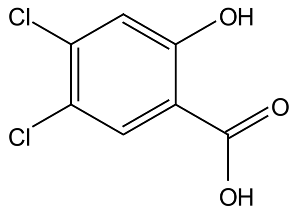

1i

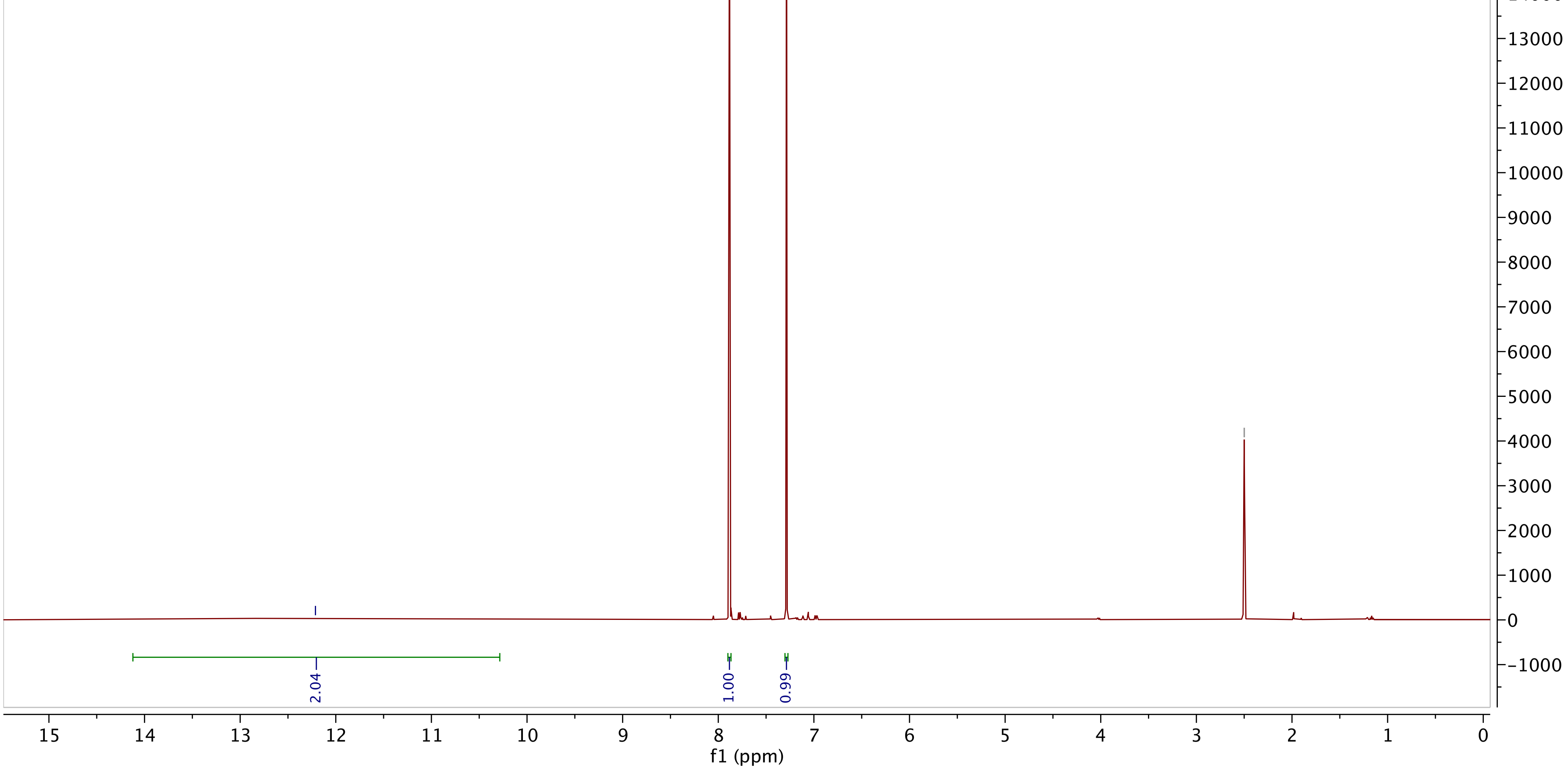

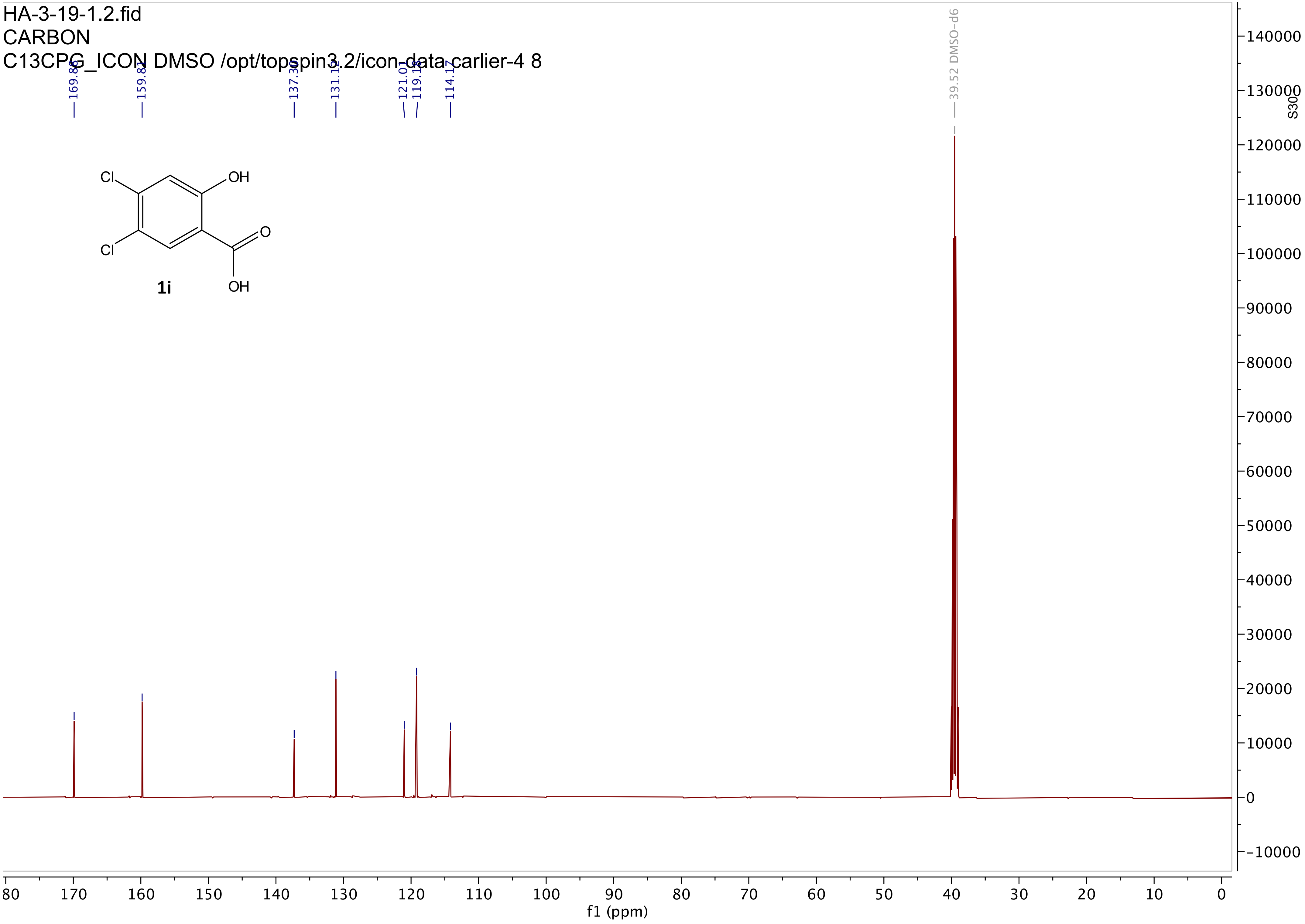
HA-3-19-1.2.fid
CARBON
C13CPG_ICON DMSO /opt/topspin3.2/icon-data carlier-4 8
169.86
159.82
137.30
131.1
121.01
119.1
114.17
39.52 DMSO-d6
Cl
Cl
OH
O
OH
1i
f1 (ppm)

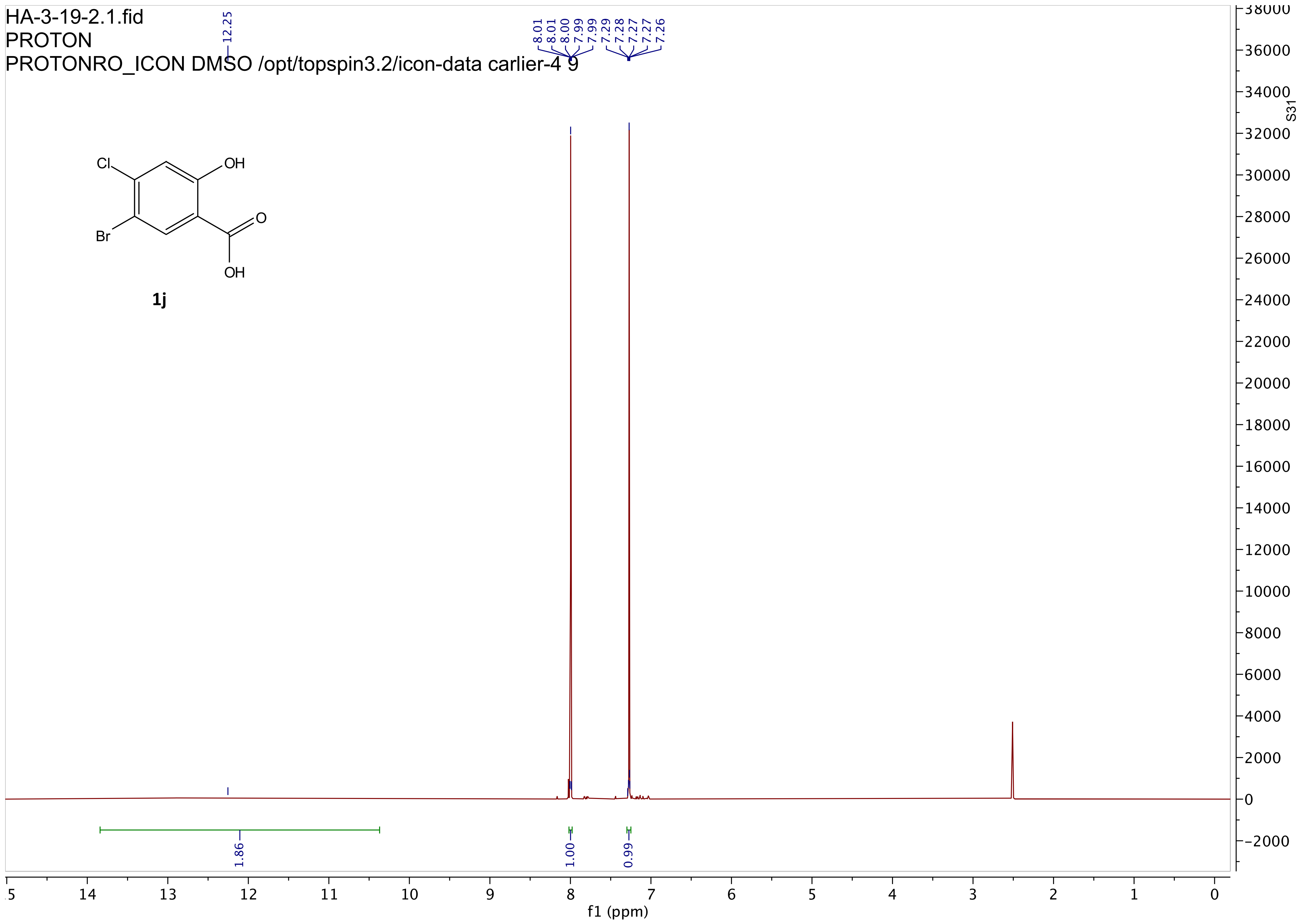
HA-3-19-2.1.fid
PROTON
PROTONRO_ICON DMSO /opt/topspin3.2/icon-data carlier-4 9
12.25
8.01
8.01
8.00
7.99
7.99
7.29
7.28
7.27
7.27
7.26
Cl
OH
O
Br
OH
1j
1.86
1.00
0.99
f1 (ppm)

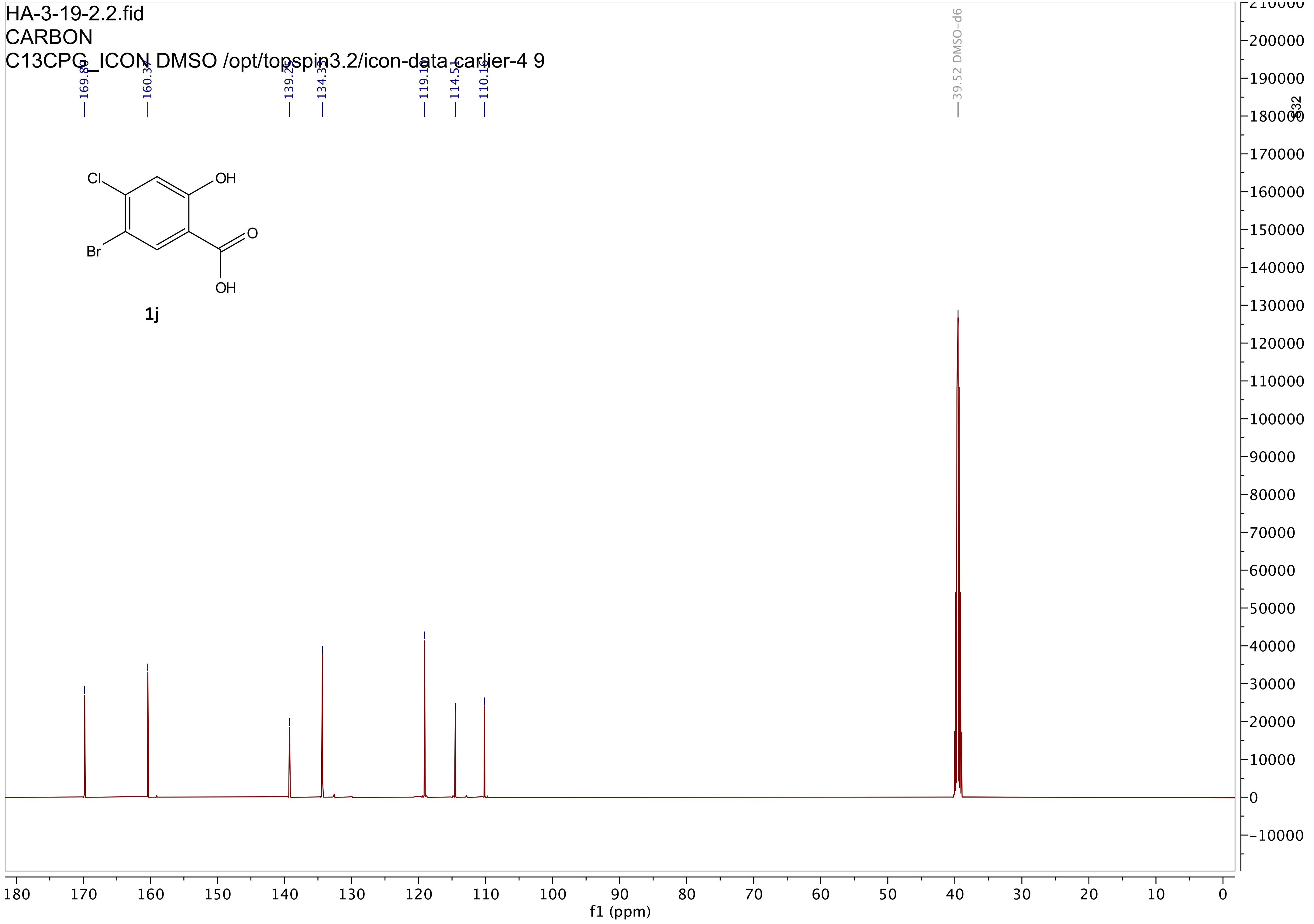
HA-3-19-2.2.fid
CARBON
C13CPG_ICON DMSO /opt/topspin3.2/icon-data carlier-4 9
169.80
160.37
139.29
134.33
119.10
114.51
110.16
39.52 DMSO-d6
Cl
OH
O
Br
OH
1j
f1 (ppm)

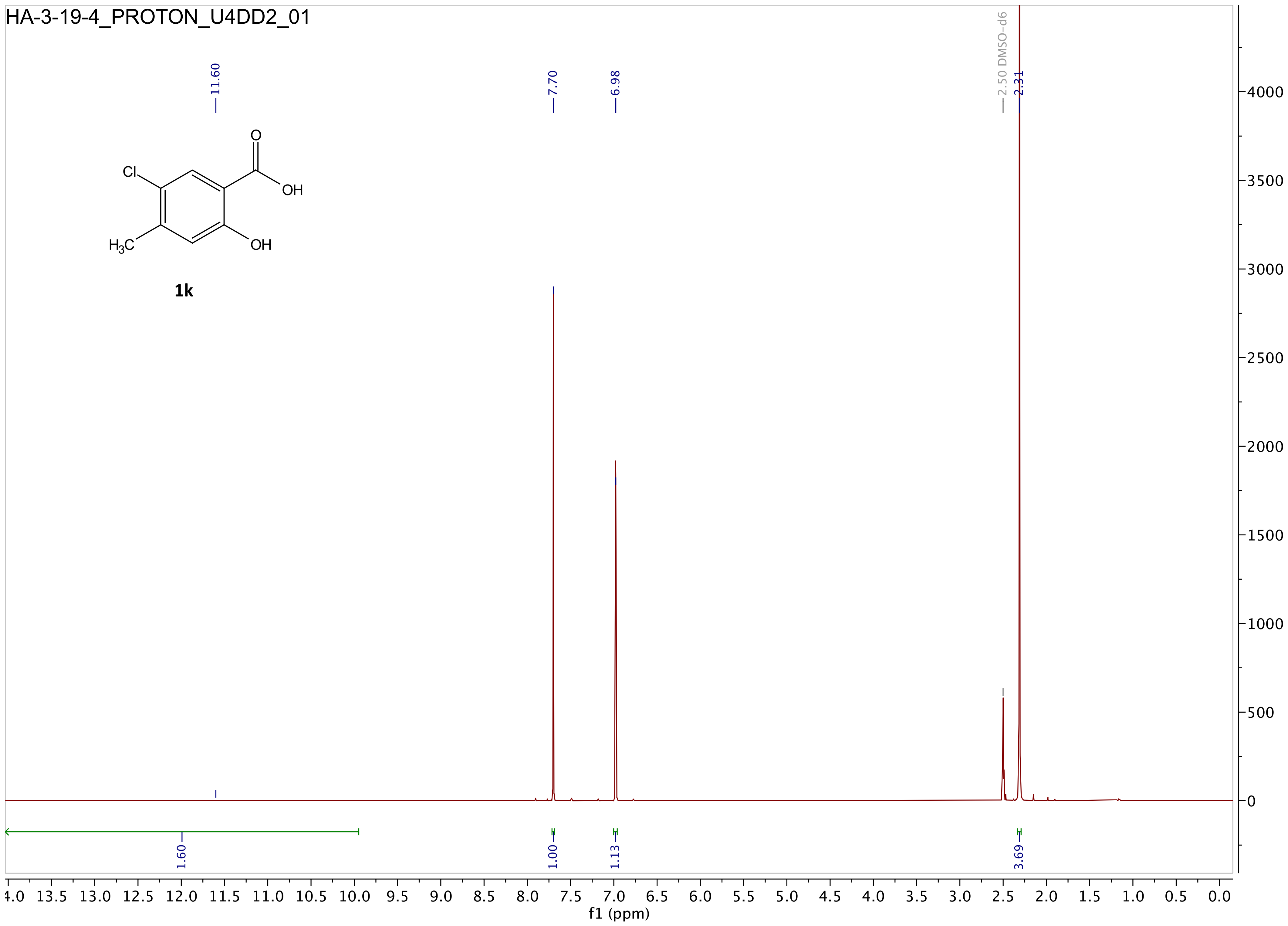
HA-3-19-4_PROTON_U4DD2_01
Cl
O
OH
OH
H_3C
1k
11.60
7.70
6.98
2.50 DMSO-d6
2.31
1.60
1.00
1.13
3.69
f1 (ppm)

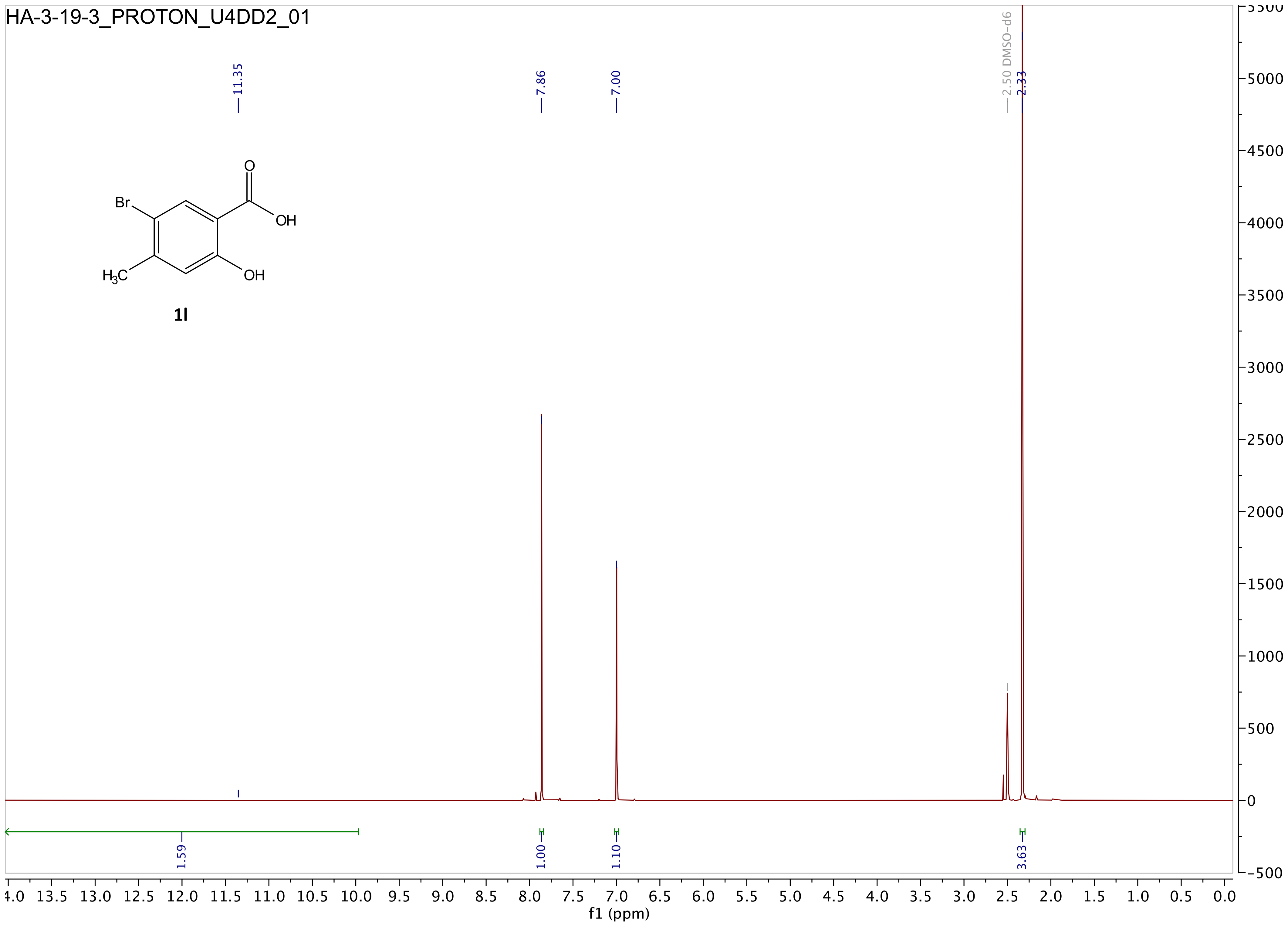

HA-3-19-3_PROTON_U4DD2_01
11.35
7.86
7.00
2.50 DMSO-d6
2.33
Br
O
OH
OH
H_3C
1l
1.59
1.00
1.10
3.63
f1 (ppm)

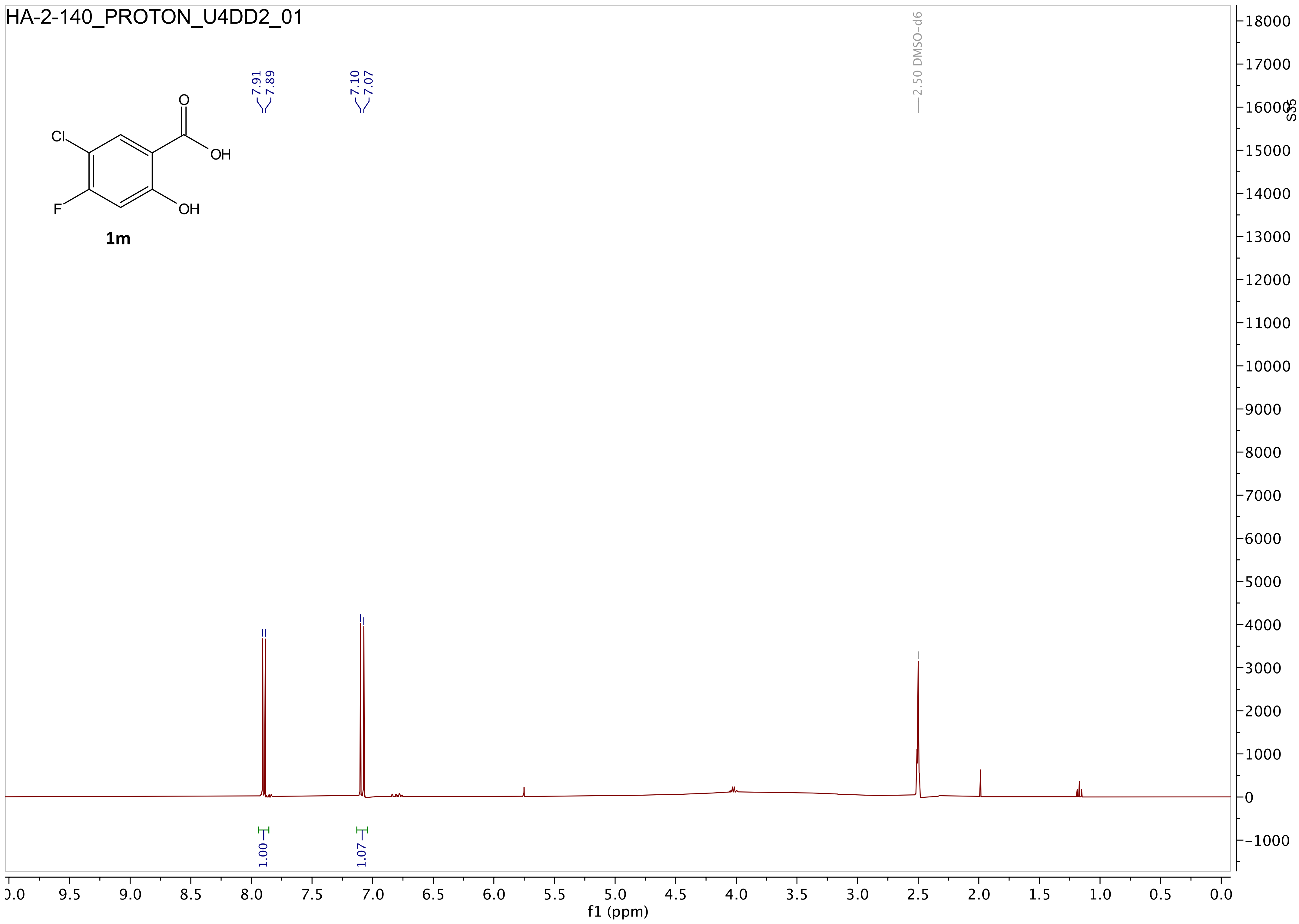
HA-2-140_PROTON_U4DD2_01
Cl
F
O
OH
OH
1m
7.91
7.89
7.10
7.07
2.50 DMSO-d6
1.00
1.07
f1 (ppm)
10.0
9.5
9.0
8.5
8.0
7.5
7.0
6.5
6.0
5.5
5.0
4.5
4.0
3.5
3.0
2.5
2.0
1.5
1.0
0.5
0.0
18000
17000
16000
15000
14000
13000
12000
11000
10000
9000
8000
7000
6000
5000
4000
3000
2000
1000
0
-1000

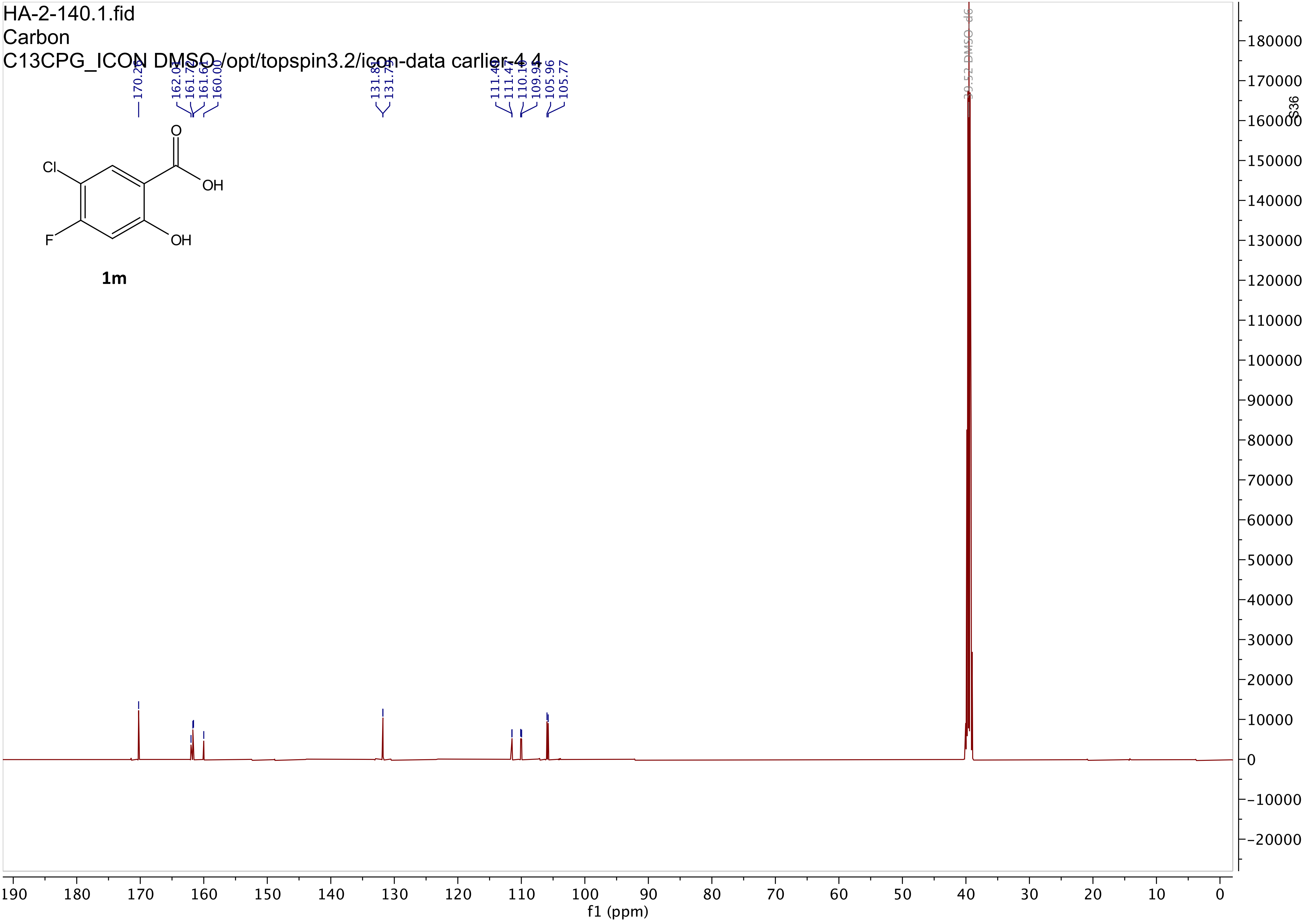
HA-2-140.1.fid
Carbon
C13CPG_ICON DMSO /opt/topspin3.2/icon-data carlier 4 4
170.26
162.03
161.72
161.61
160.00
131.81
131.79
111.49
111.47
110.16
109.95
105.96
105.77
39.52 DMSO_d6
Cl
F
O
OH
OH
1m
f1 (ppm)

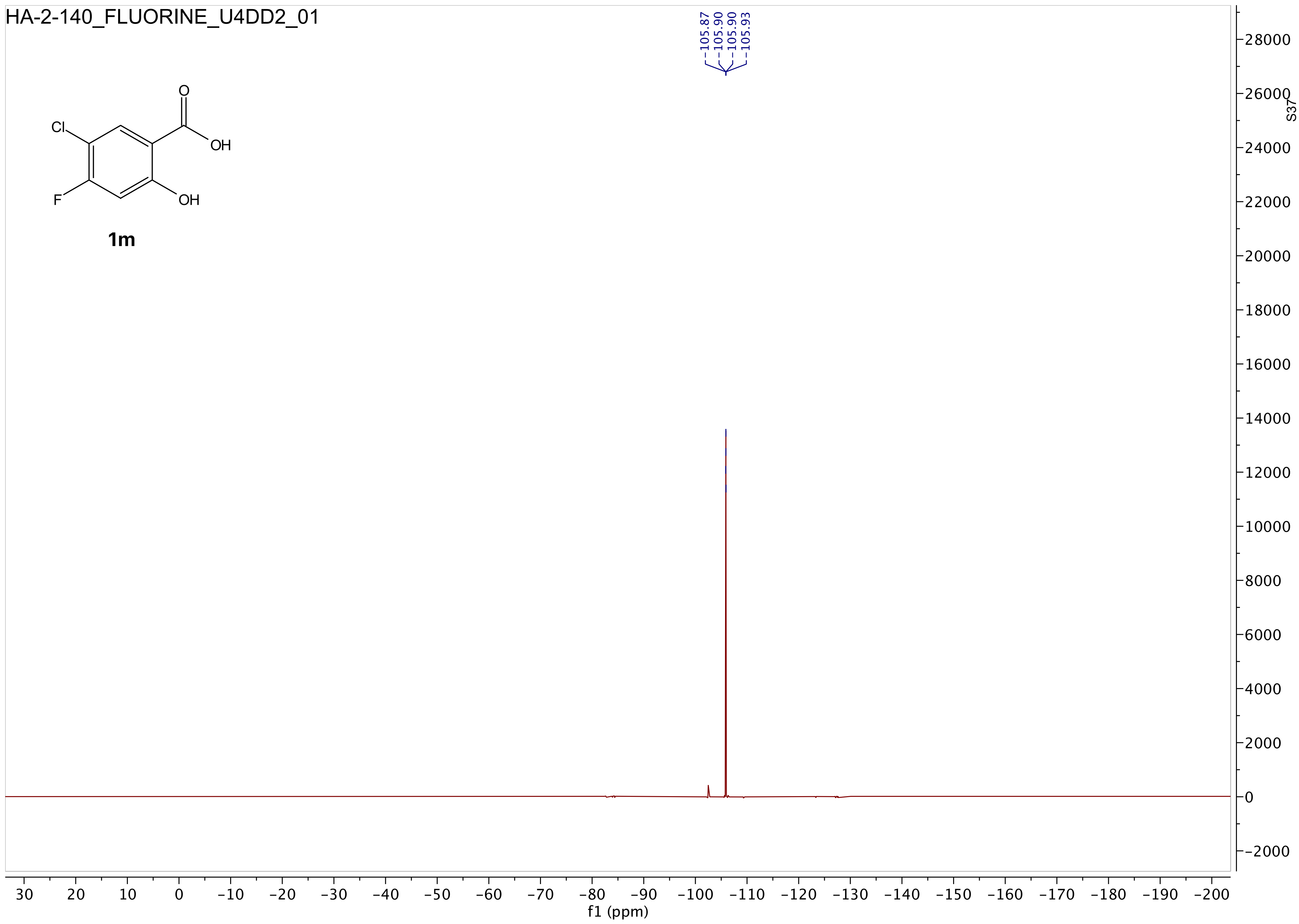
HA-2-140_FLUORINE_U4DD2_01
Cl
F
O
OH
OH
1m
−105.87
−105.90
−105.90
−105.93
f1 (ppm)

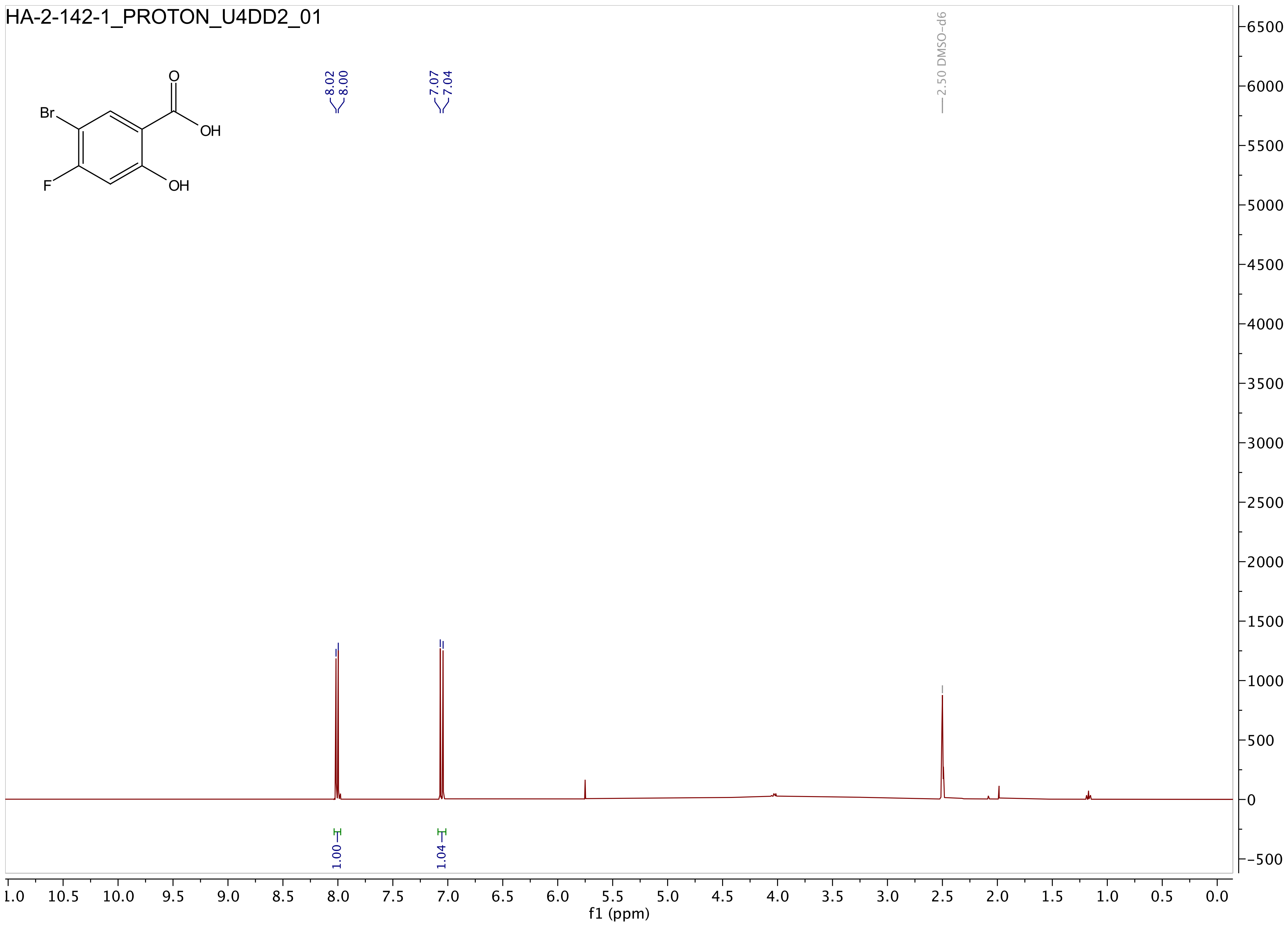

HA-2-142-1_PROTON_U4DD2_01
Br
F
O
OH
OH
8.02
8.00
7.07
7.04
2.50 DMSO-d6
1.00
1.04
f1 (ppm)

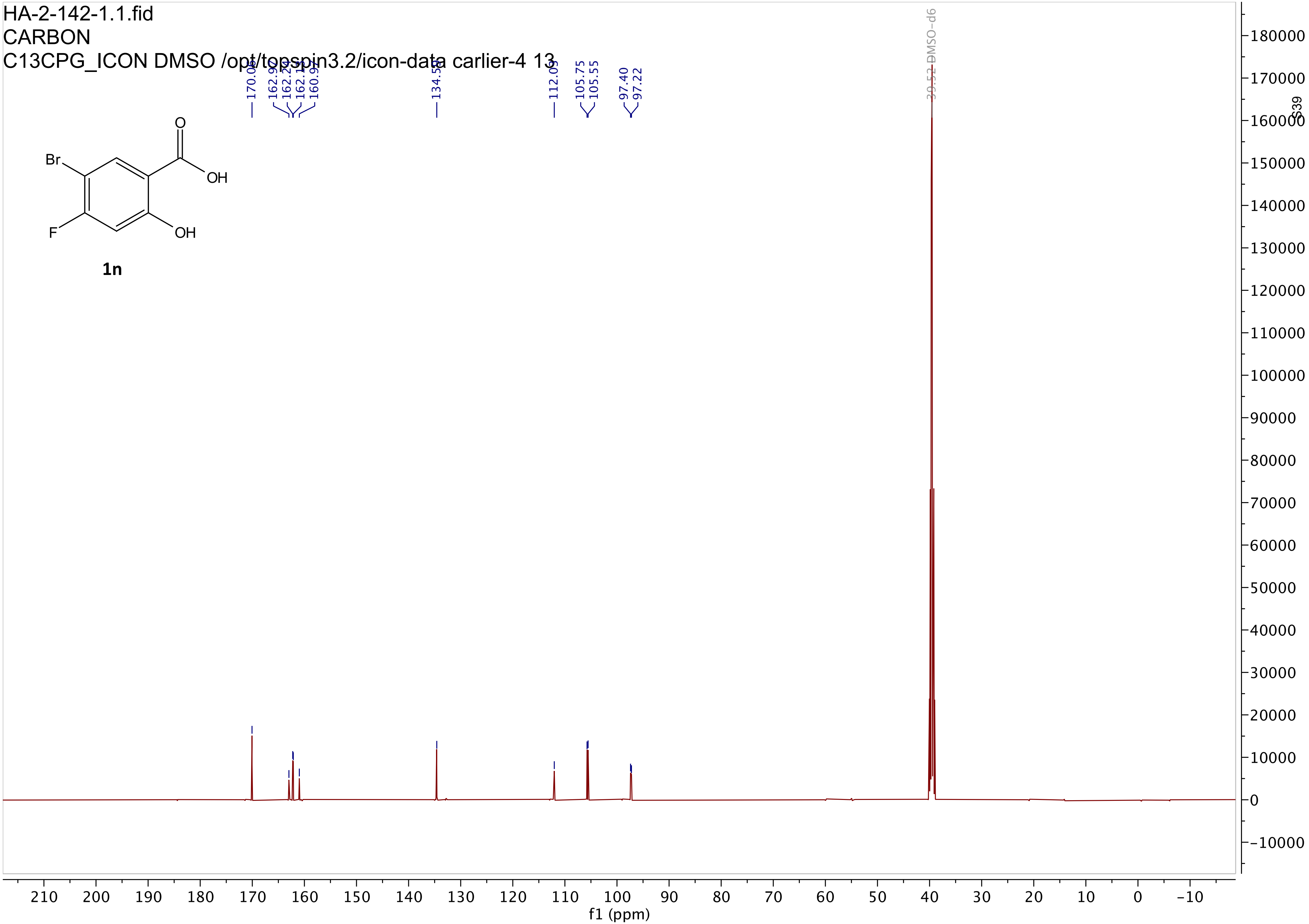
HA-2-142-1.1.fid
CARBON
C13CPG_ICON DMSO /opt/topspin3.2/icon-data carlier-4 13
170.06
162.97
162.24
162.13
160.97
134.58
112.05
105.75
105.55
97.40
97.22
39.52 DMSO-d6
Br
F
OH
OH
O
f1 (ppm)
1n

HA-2-142-1_FLUORINE_U4DD2_01

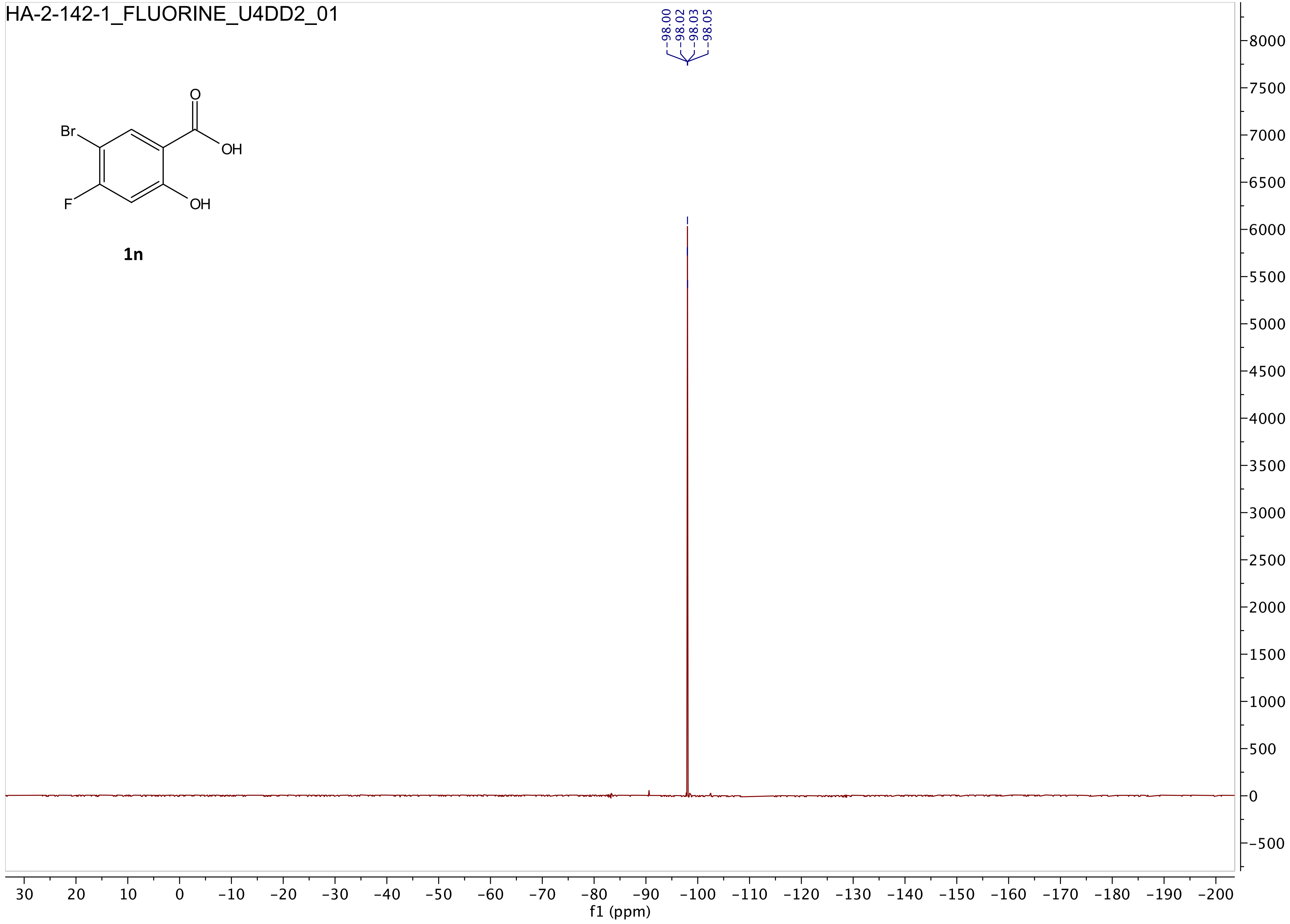

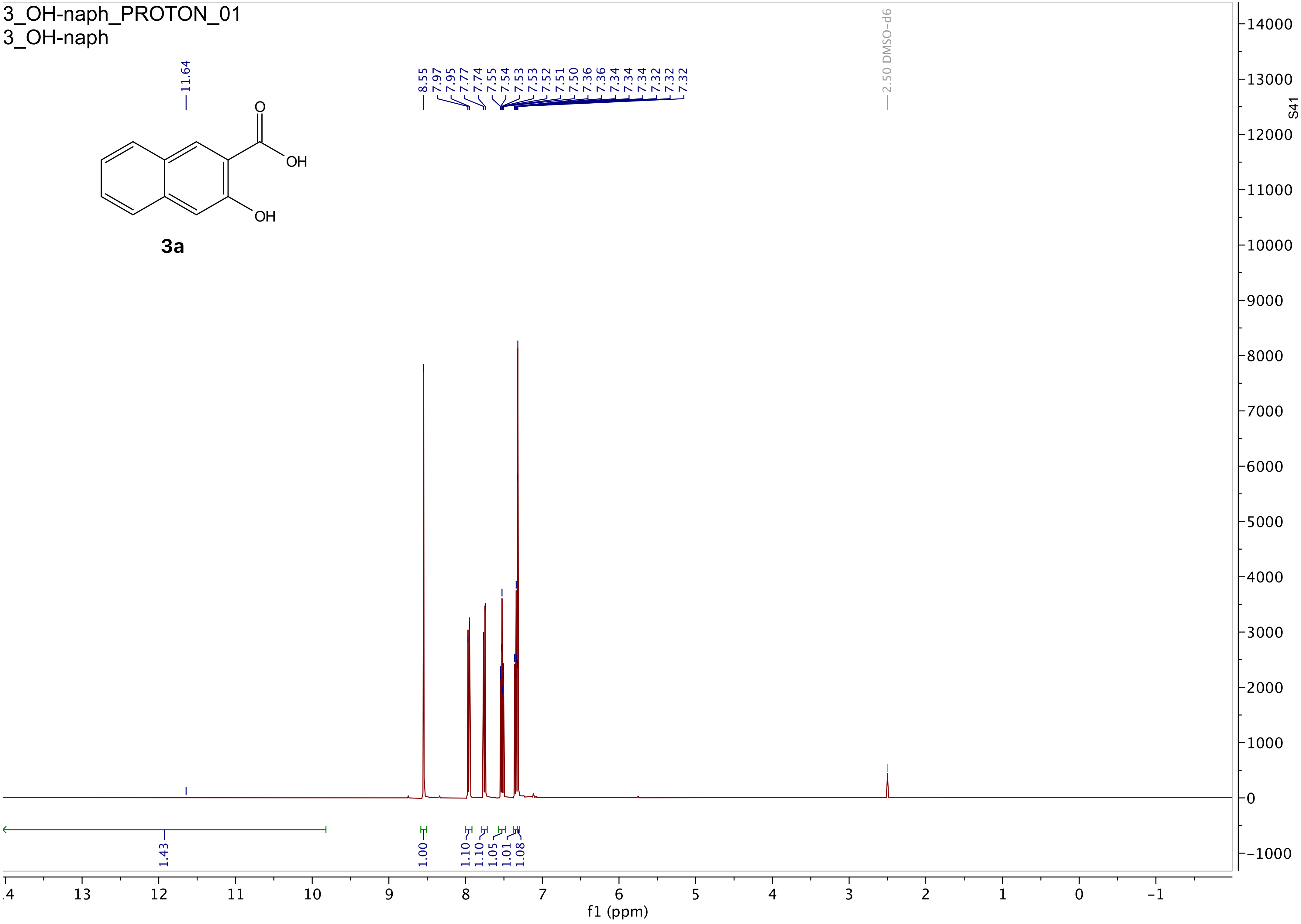
3_OH-naph_PROTON_01
3_OH-naph
11.64
8.55
7.97
7.95
7.77
7.74
7.55
7.54
7.53
7.53
7.52
7.51
7.50
7.36
7.36
7.34
7.34
7.34
7.32
7.32
7.32
2.50 DMSO-d6
O
OH
OH
3a
1.43
1.00
1.10
1.10
1.05
1.01
1.08
f1 (ppm)

HA-3-18-1.1.fid
PROTON
PROTONRO_ICON DMSO /opt/topspin3.2/icon-data carlier-4 7

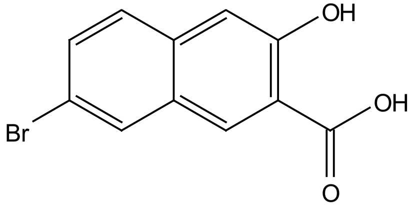

3b

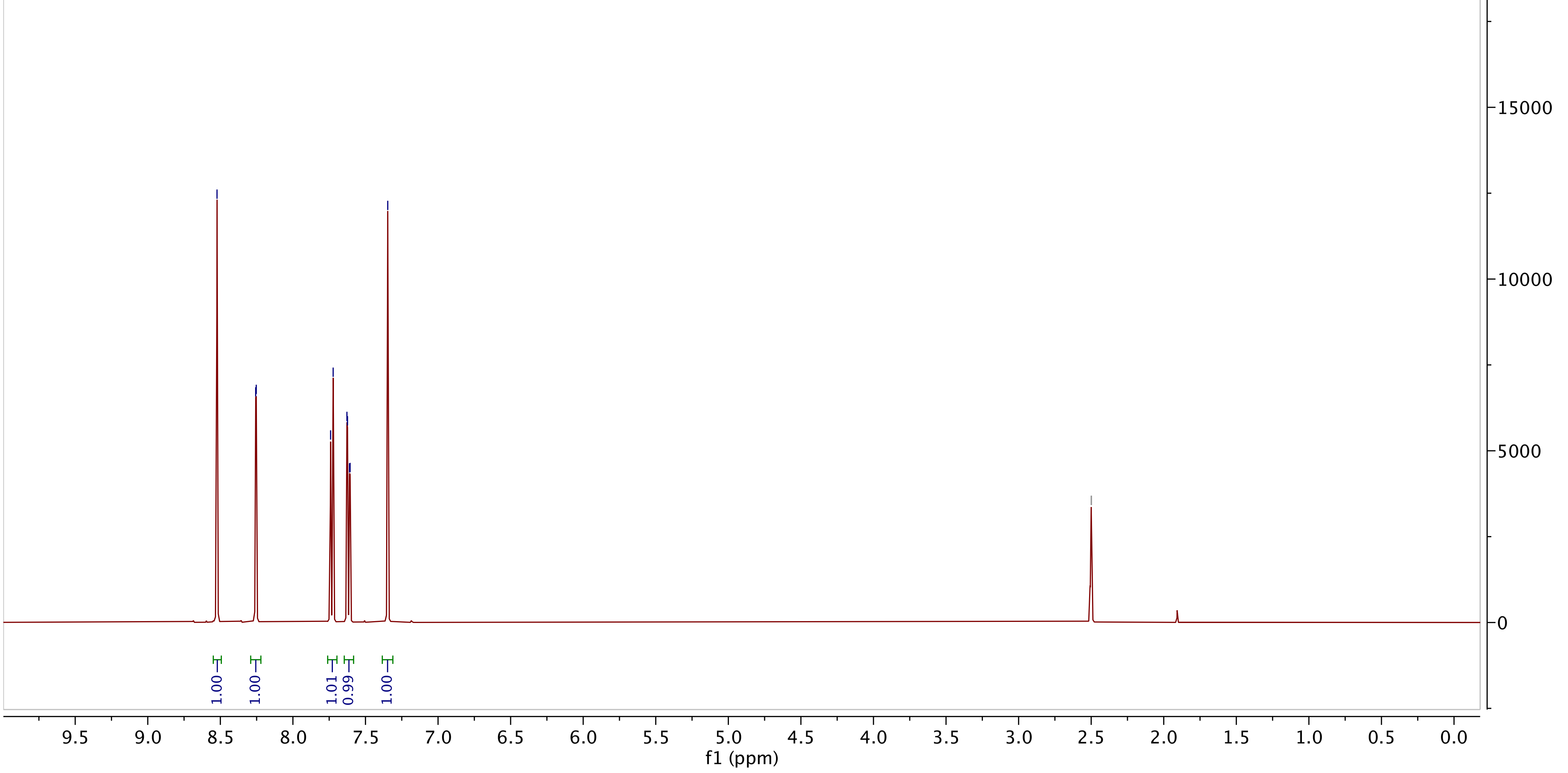

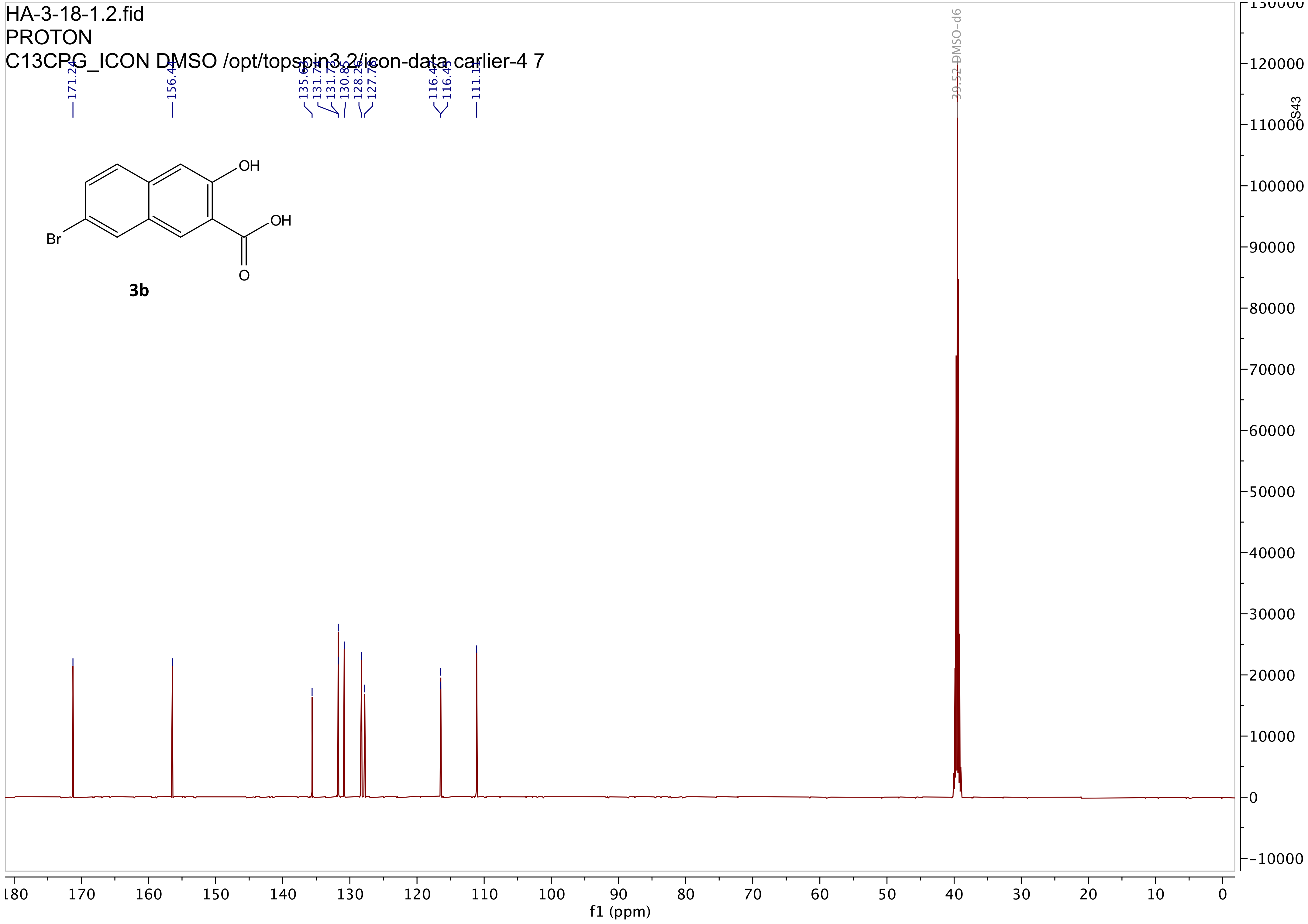
HA-3-18-1.2.fid
PROTON
C13CPG_ICON DMSO /opt/topspin3.2/icon-data carlier-4 7
171.24
156.44
130.85
39.52 DMSO-d6
Br
OH
OH
O
3b
f1 (ppm)

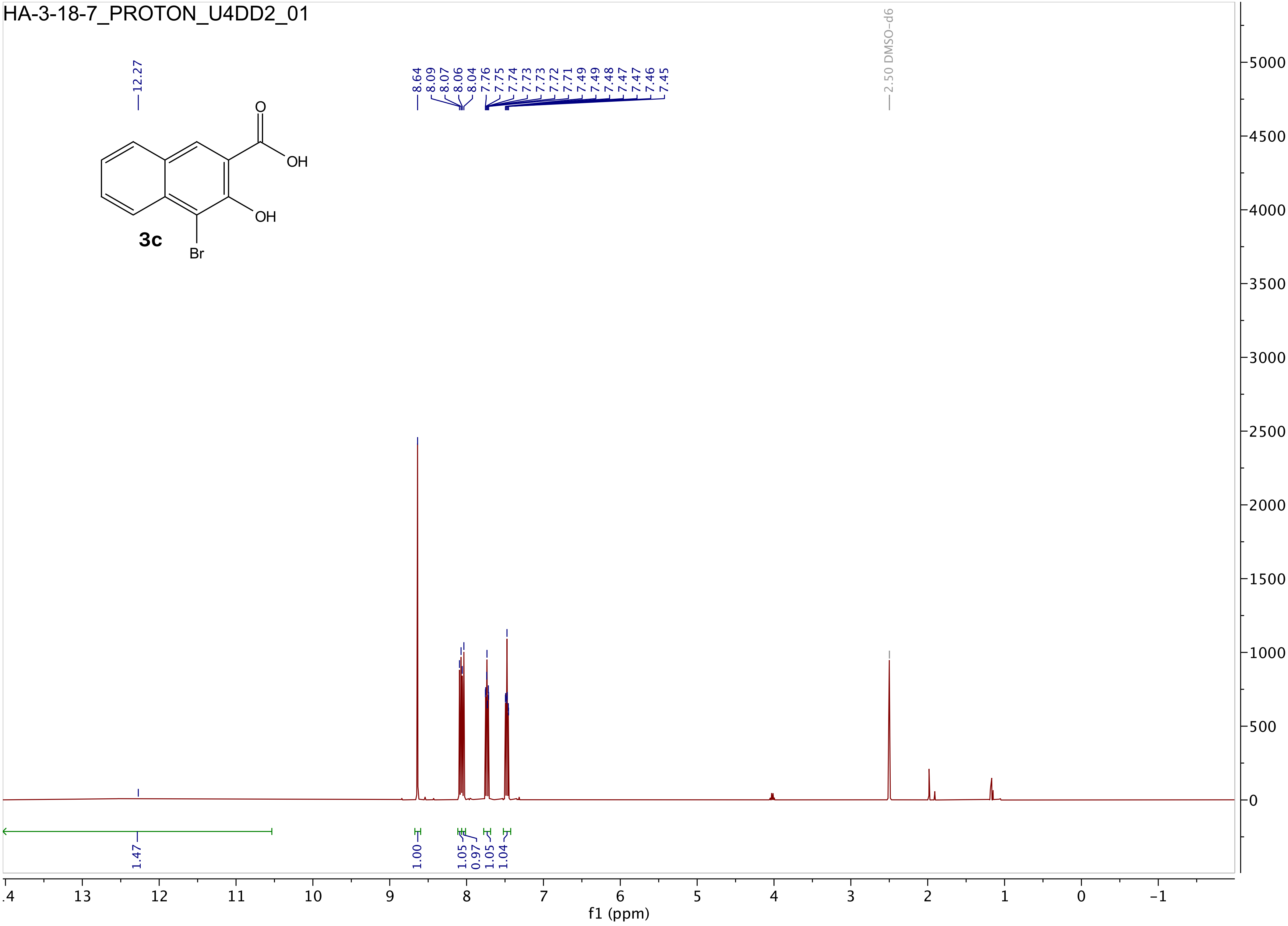

HA-3-18-7_PROTON_U4DD2_01
3c
OH
OH
O
Br
12.27
8.64
8.09
8.07
8.06
8.04
7.76
7.75
7.74
7.73
7.73
7.72
7.71
7.49
7.49
7.48
7.47
7.47
7.46
7.45
2.50 DMSO-d6
1.47
1.00
1.05
0.97
1.05
1.04
f1 (ppm)

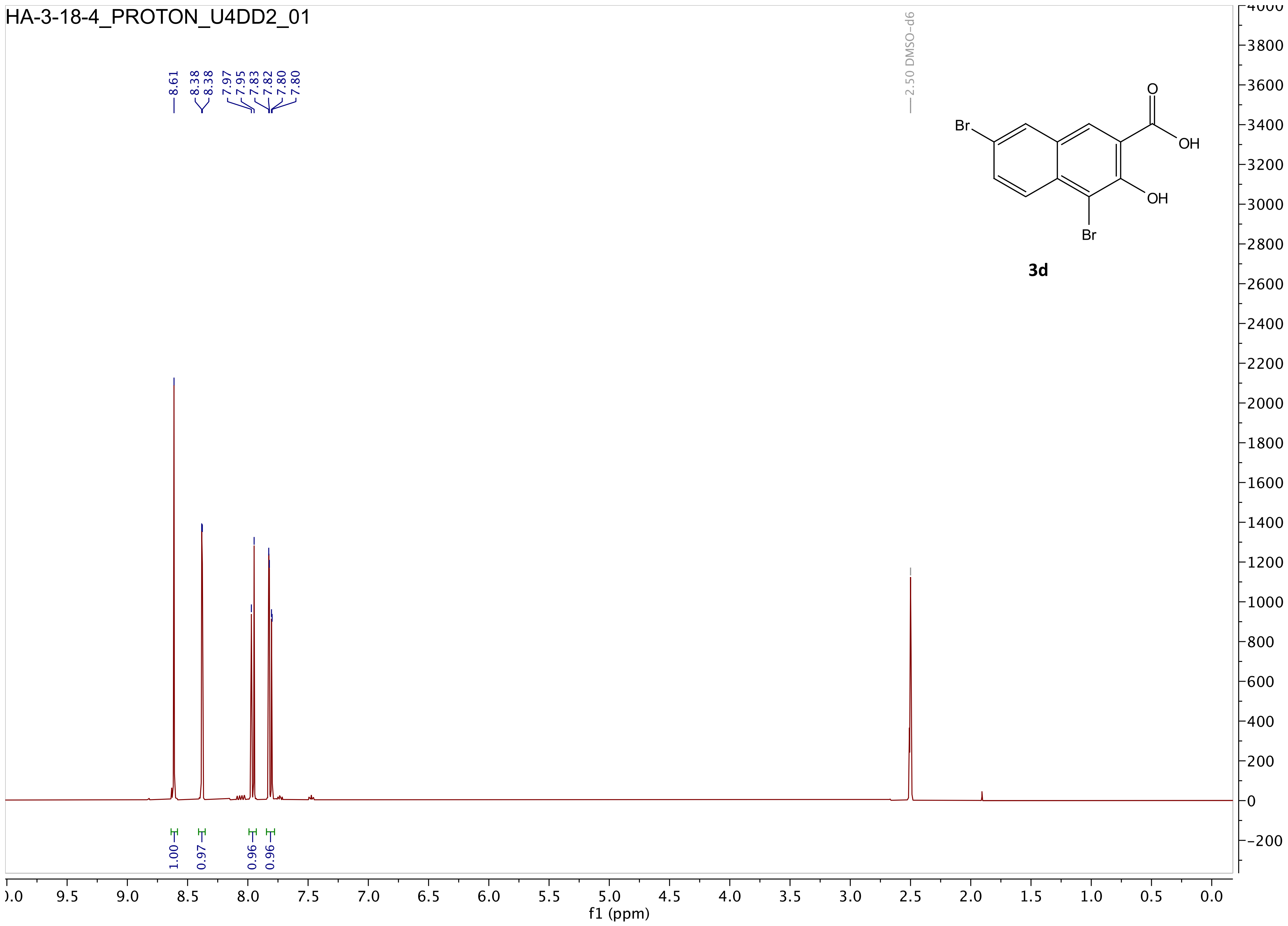
HA-3-18-4_PROTON_U4DD2_01
8.61
8.38
8.38
7.97
7.95
7.83
7.82
7.80
7.80
2.50 DMSO-d6
Br
O
OH
OH
Br
1.00
0.97
0.96
0.96
f1 (ppm)

3d

HA-3-18-5_PROTON_U4DD2_01

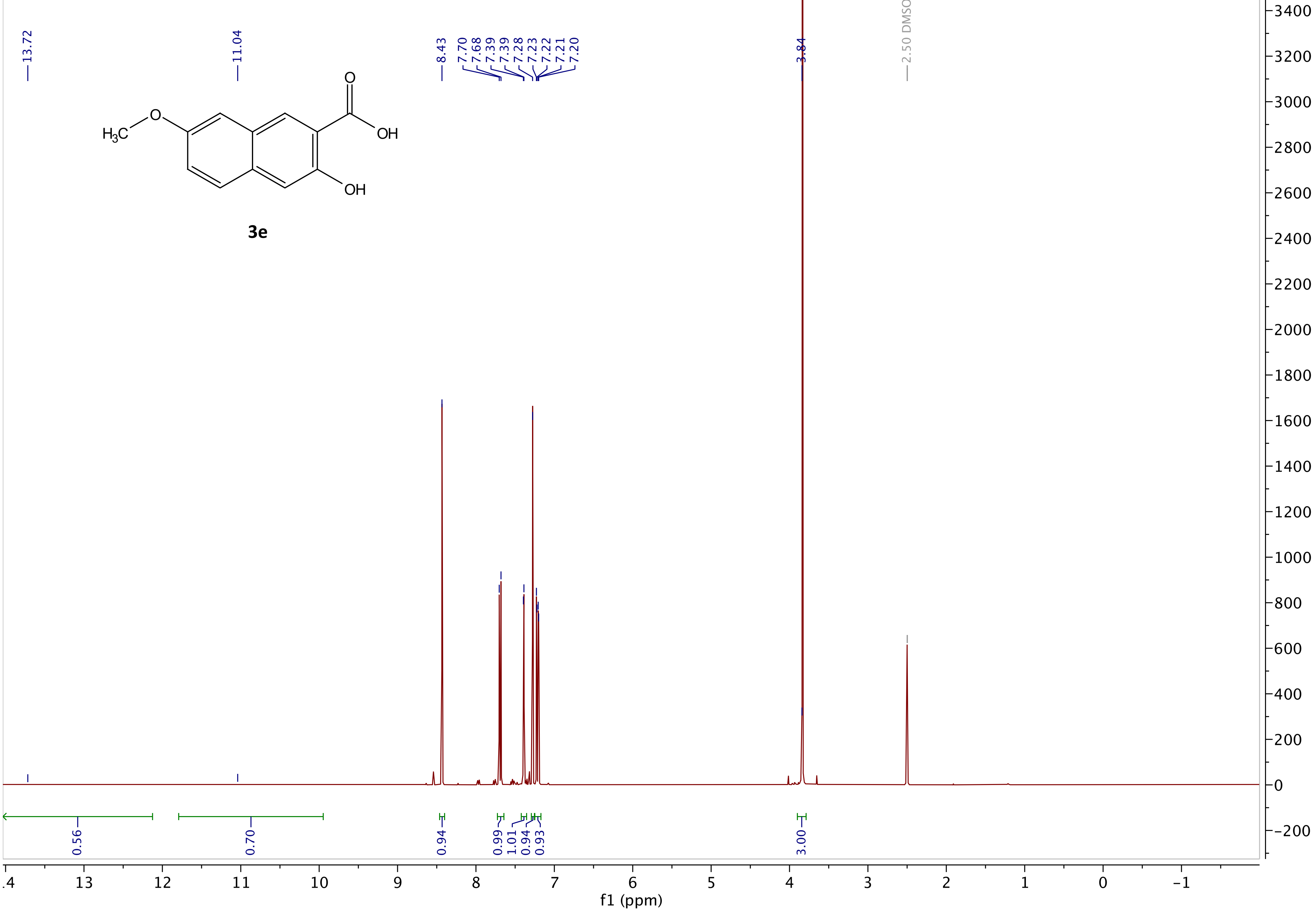

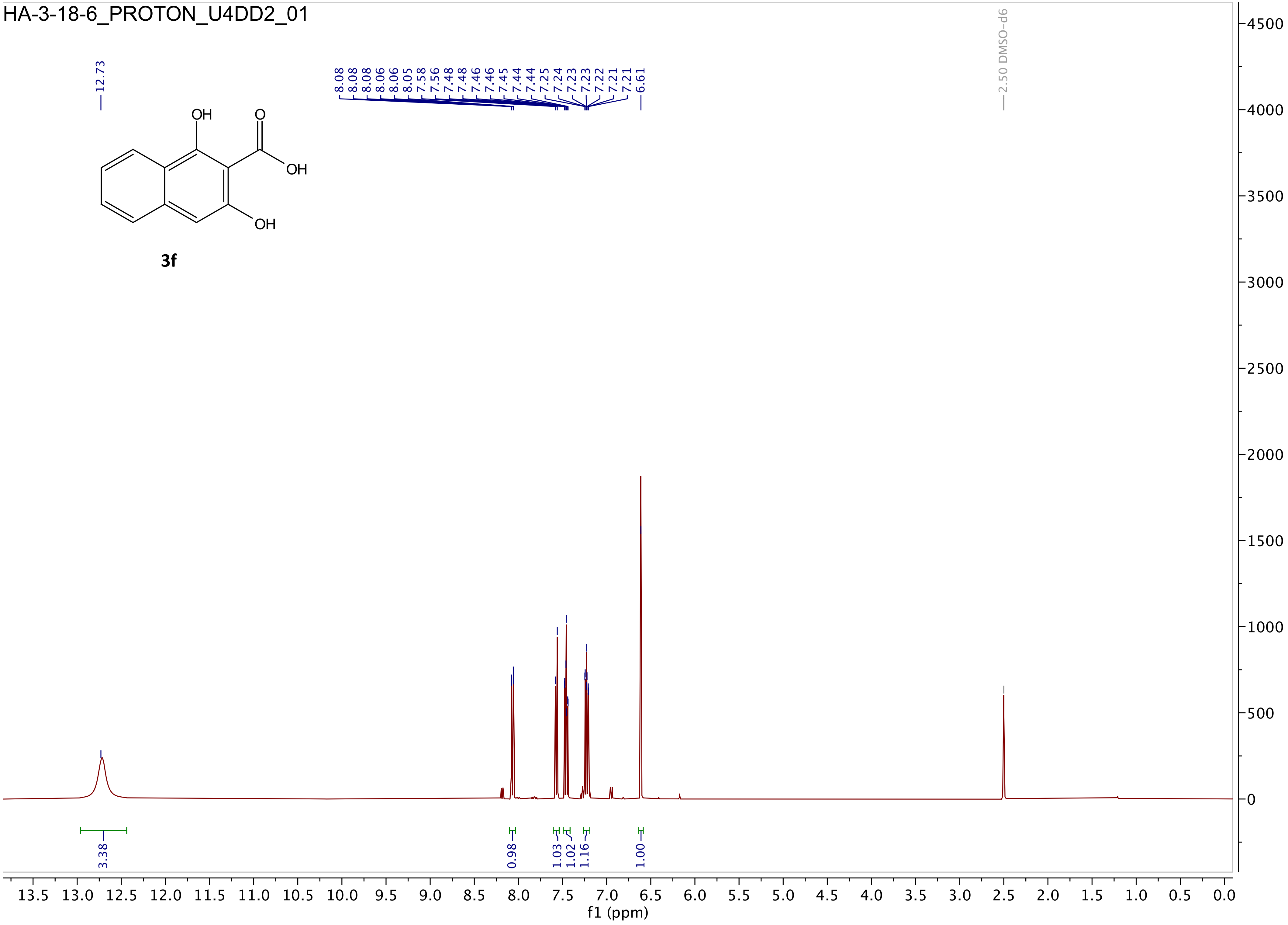
HA-3-18-6_PROTON_U4DD2_01
12.73
8.08
8.08
8.08
8.06
8.06
8.05
7.58
7.56
7.48
7.48
7.46
7.46
7.45
7.44
7.44
7.25
7.24
7.23
7.23
7.22
7.21
7.21
6.61
2.50 DMSO-d6
OH
O
OH
OH
3f
3.38
0.98
1.03
1.02
1.16
1.00
13.5 13.0 12.5 12.0 11.5 11.0 10.5 10.0 9.5 9.0 8.5 8.0 7.5 7.0 6.5 6.0 5.5 5.0 4.5 4.0 3.5 3.0 2.5 2.0 1.5 1.0 0.5 0.0
f1 (ppm)
4500 4000 3500 3000 2500 2000 1500 1000 500 0

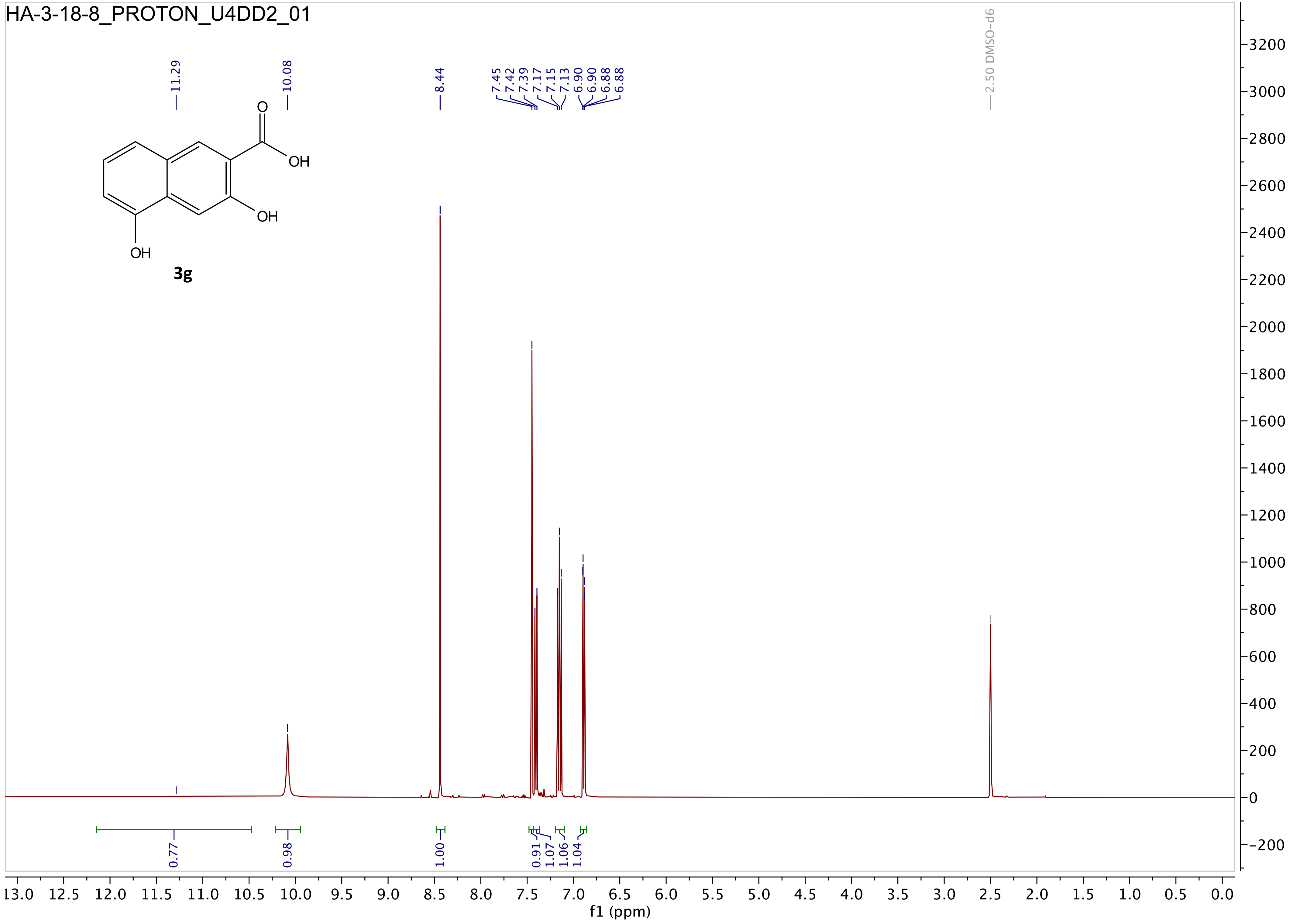
HA-3-18-8_PROTON_U4DD2_01
11.29
10.08
8.44
7.45
7.42
7.39
7.17
7.15
7.13
6.90
6.90
6.88
6.88
2.50 DMSO-d6
OH
OH
OH
O
3g
0.77
0.98
1.00
0.91
1.07
1.06
1.04
f1 (ppm)

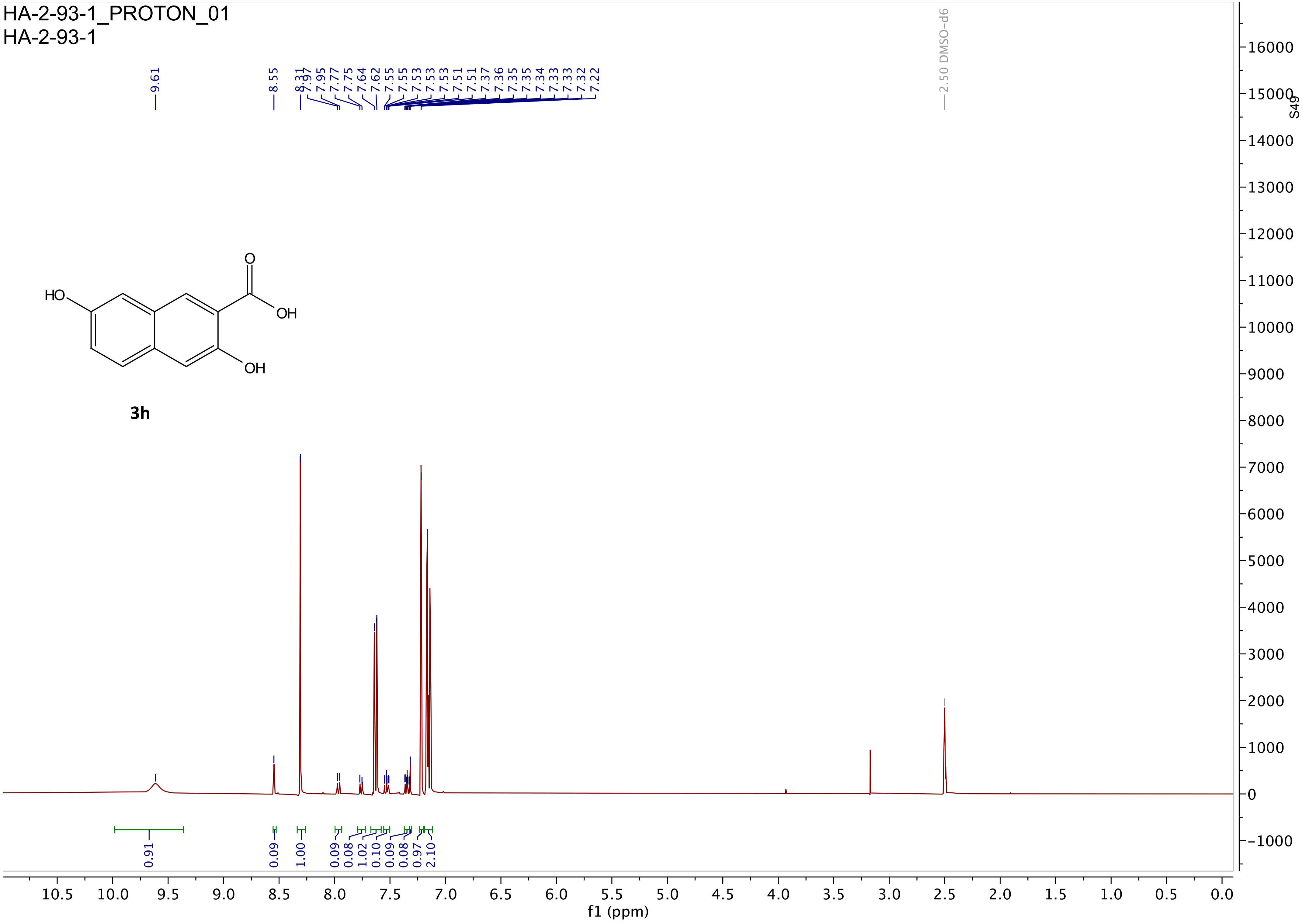

HA-2-93-1_PROTON_01
HA-2-93-1
9.61
8.55
8.31
7.97
7.95
7.77
7.75
7.64
7.62
7.55
7.55
7.53
7.53
7.53
7.51
7.51
7.37
7.36
7.35
7.35
7.34
7.33
7.33
7.32
7.22
2.50 DMSO-d6
HO
O
OH
OH
3h
0.91
0.09
1.00
0.09
0.08
1.02
0.10
0.09
0.08
0.97
2.10
f1 (ppm)

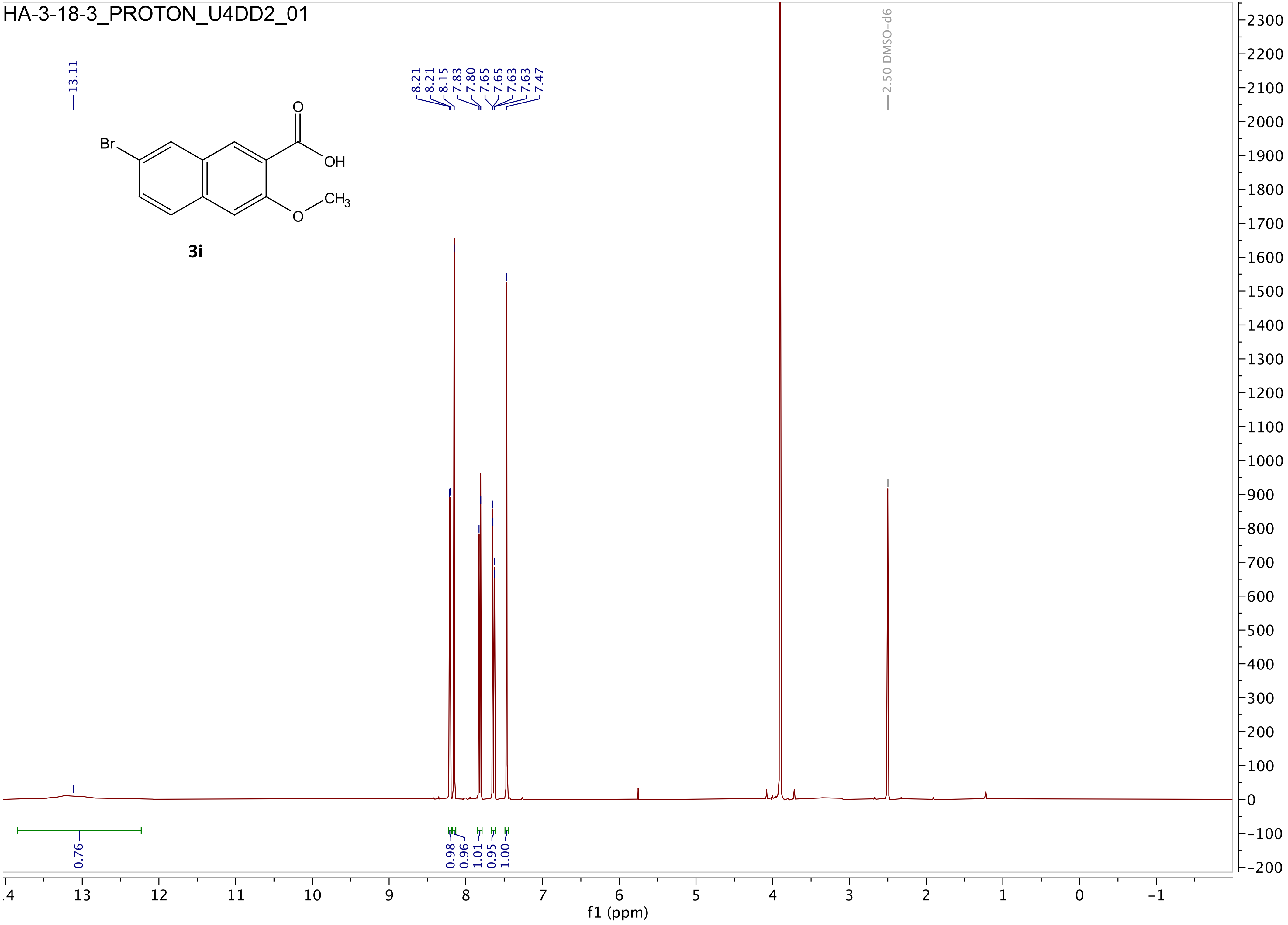
HA-3-18-3_PROTON_U4DD2_01
13.11
8.21
8.21
8.15
7.83
7.80
7.65
7.65
7.63
7.63
7.47
2.50 DMSO-d6
Br
O
OH
O
CH3
3i
0.76
0.98
0.96
1.01
0.95
1.00
f1 (ppm)

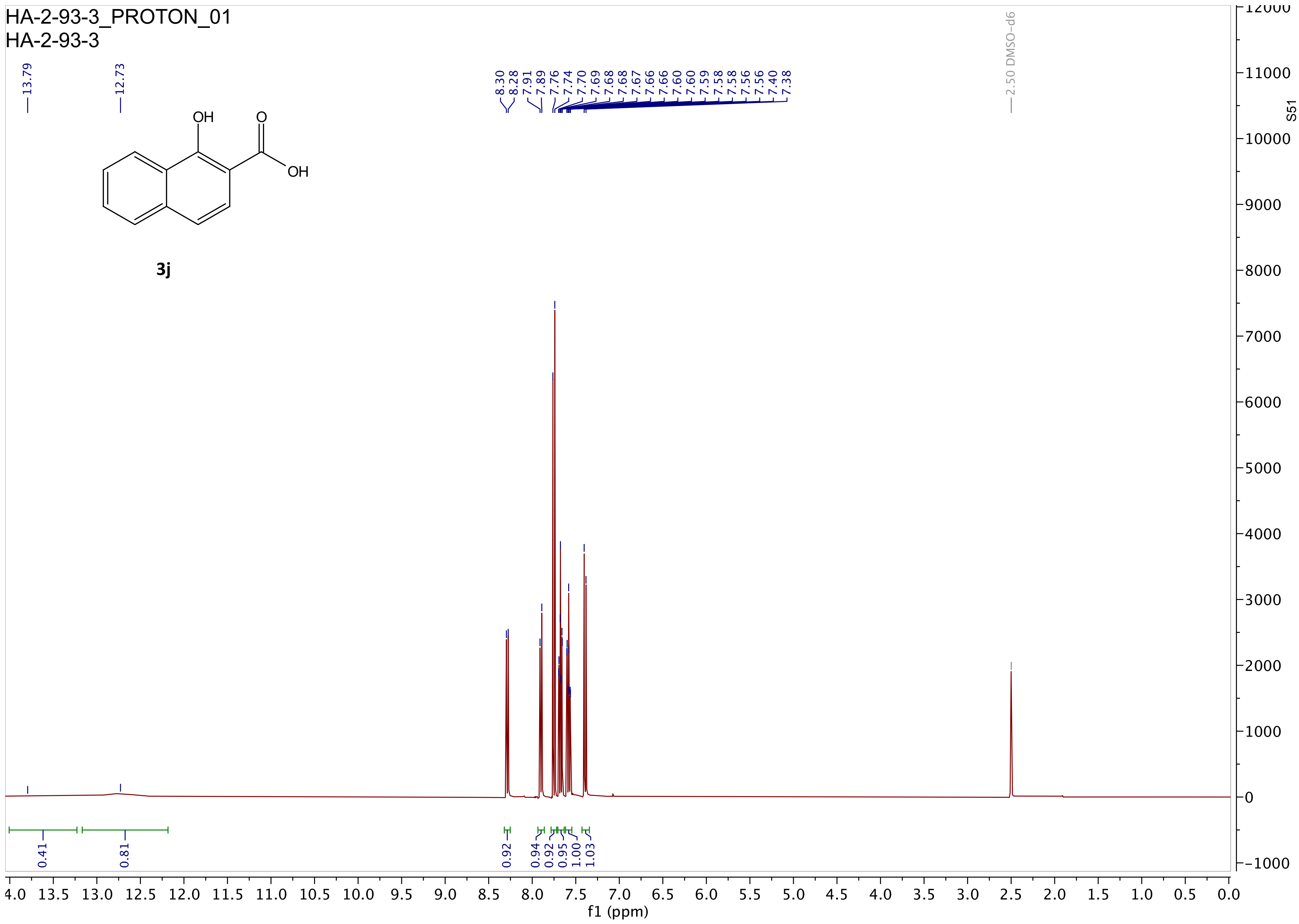
HA-2-93-3_PROTON_01
HA-2-93-3
13.79
12.73
8.30
8.28
7.91
7.89
7.76
7.74
7.70
7.69
7.68
7.68
7.67
7.66
7.66
7.60
7.60
7.59
7.58
7.58
7.56
7.56
7.40
7.38
2.50 DMSO-d6
OH
O
OH
3j
0.41
0.81
0.92
0.94
0.92
0.95
1.00
1.03
f1 (ppm)

HA-2-93-2_PROTON_01
HA-2-93-2

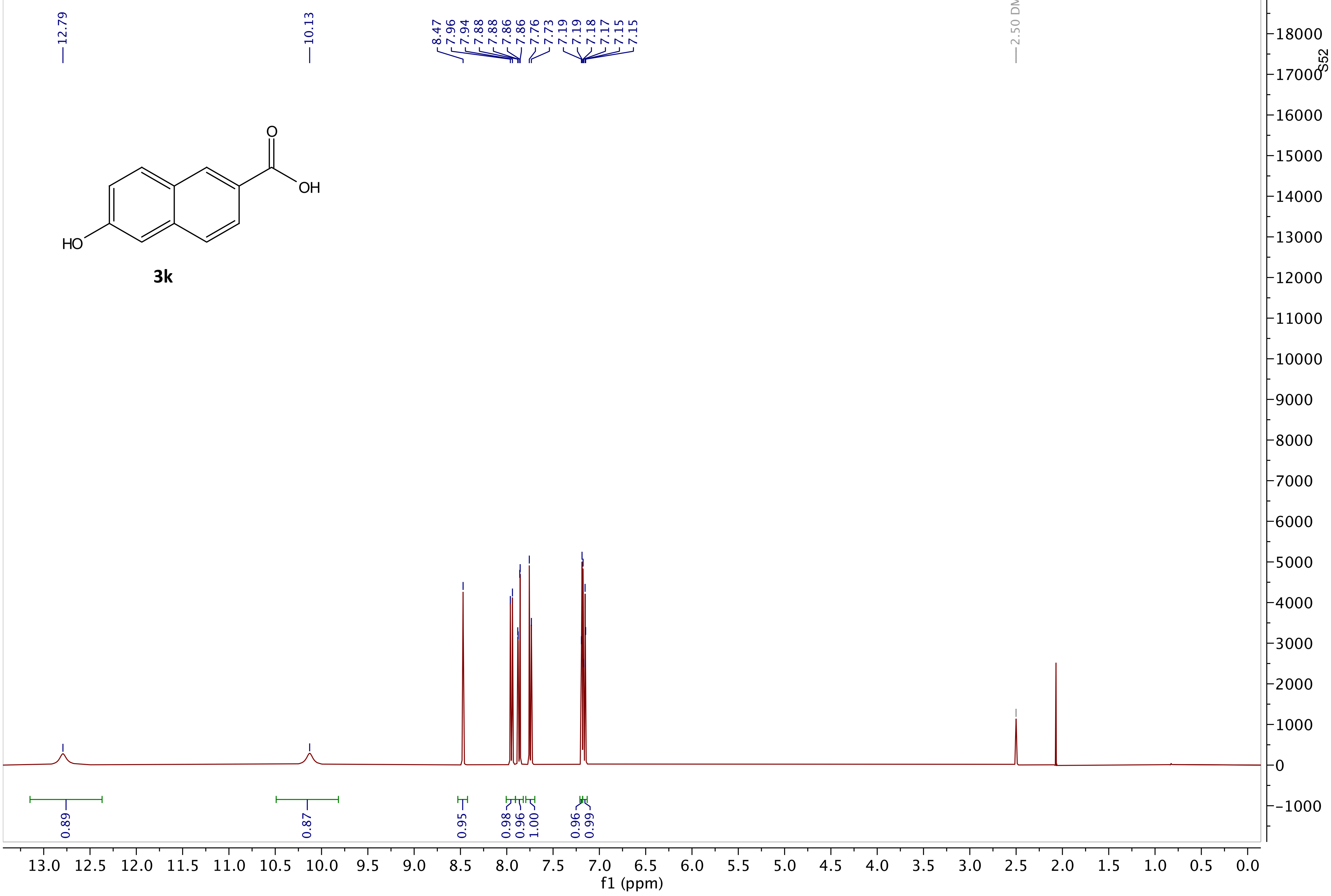

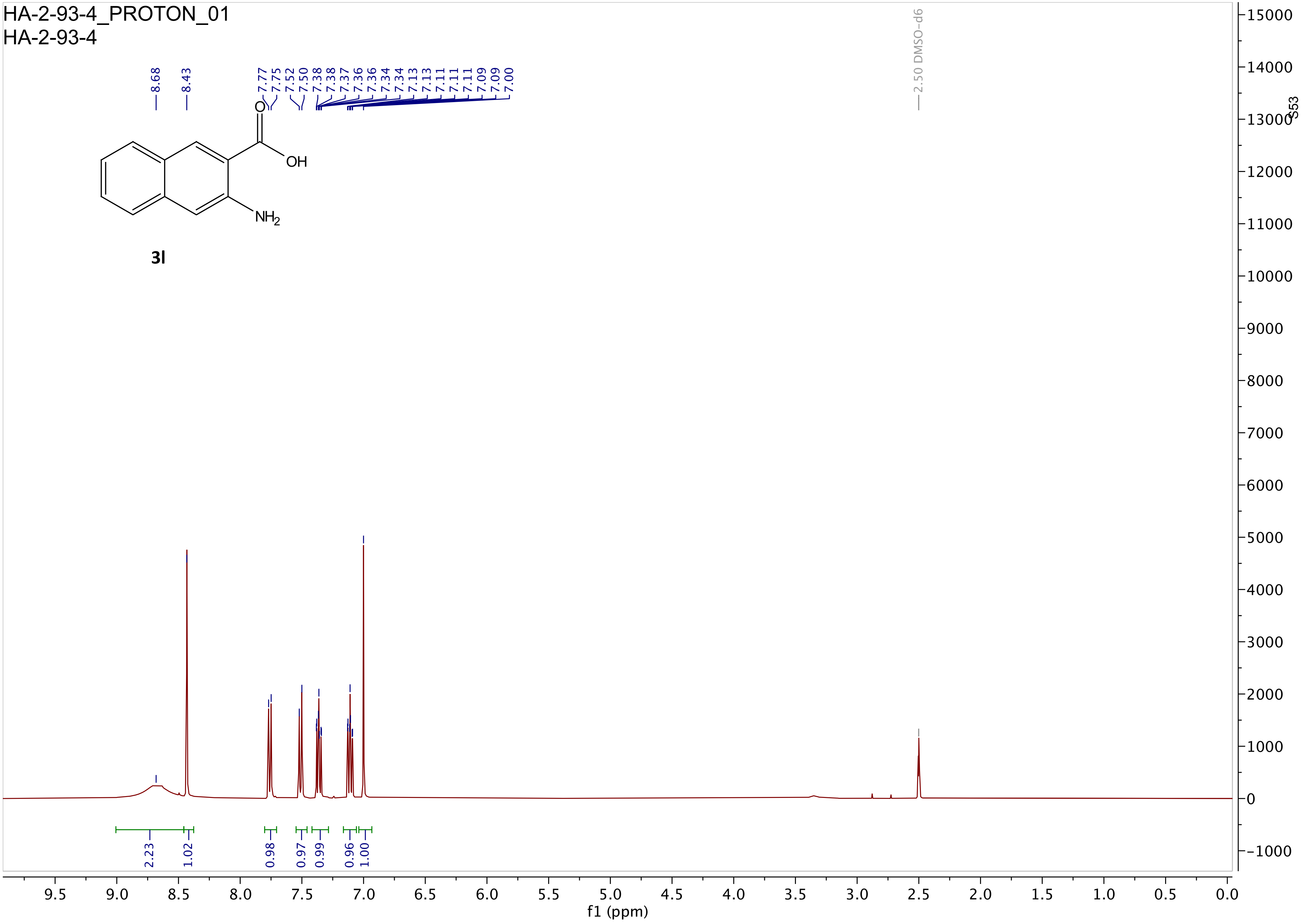

HA-2-93-4_PROTON_01
HA-2-93-4
8.68
8.43
7.77
7.75
7.52
7.50
7.38
7.38
7.37
7.36
7.36
7.34
7.34
7.13
7.13
7.11
7.11
7.11
7.09
7.09
7.00
2.50 DMSO-d6
O
OH
NH2
3l
2.23
1.02
0.98
0.97
0.99
0.96
1.00
f1 (ppm)

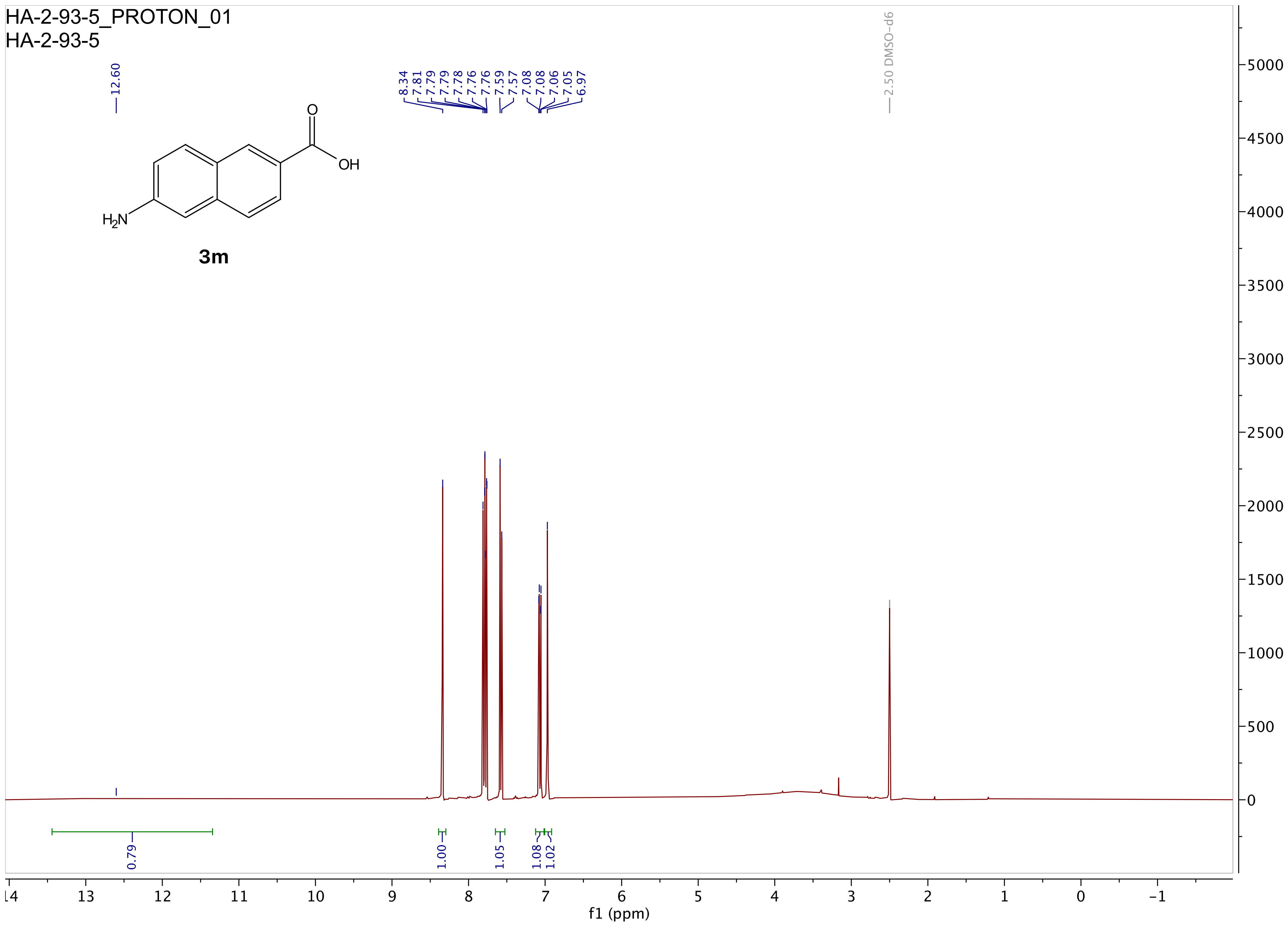

HA-2-93-5_PROTON_01
HA-2-93-5
12.60
8.34
7.81
7.79
7.79
7.78
7.76
7.76
7.59
7.57
7.08
7.08
7.06
7.05
6.97
2.50 DMSO-d6
O
OH
H_2N
3m
0.79
1.00
1.05
1.08
1.02
f1 (ppm)

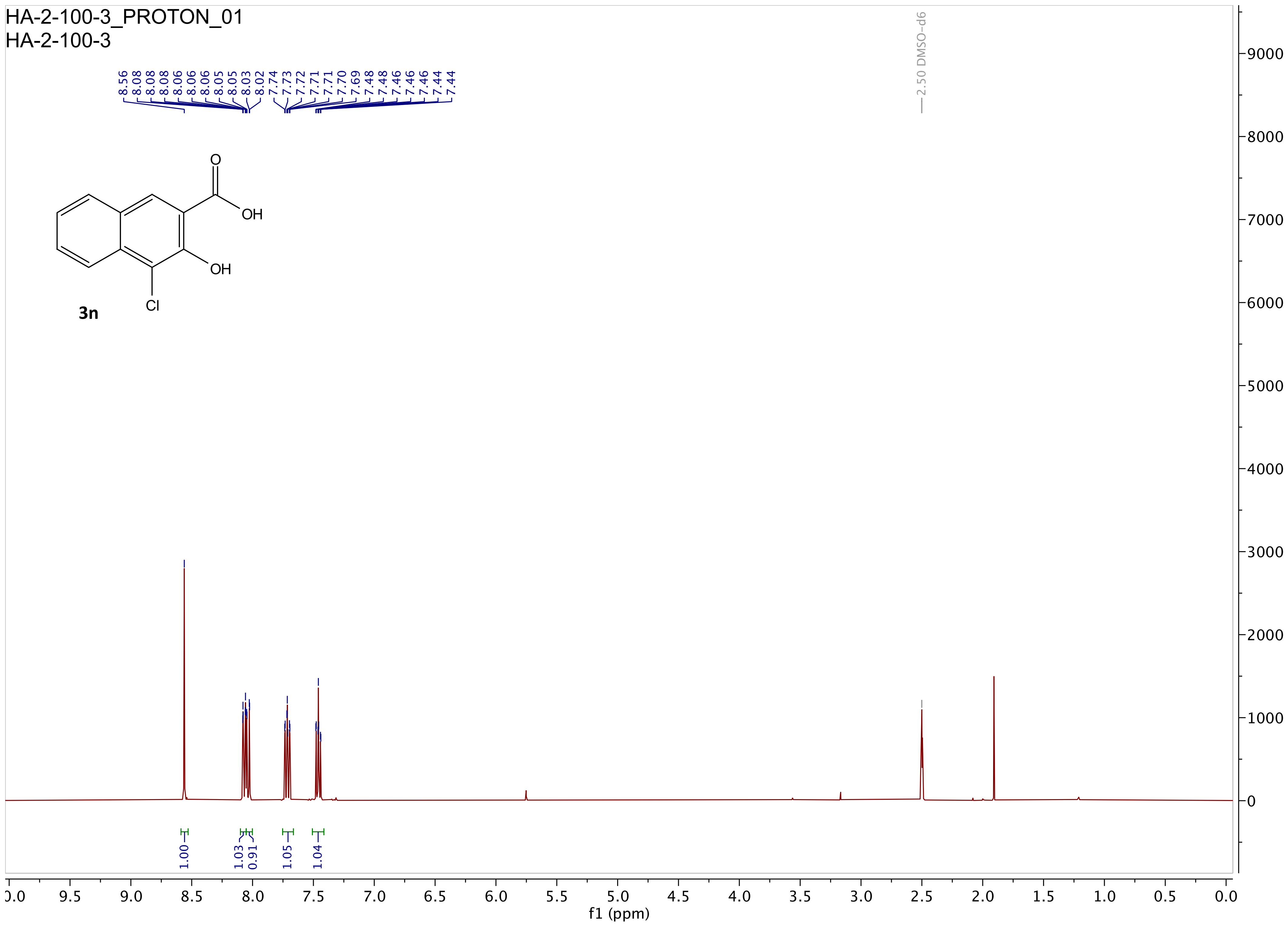
HA-2-100-3_PROTON_01
HA-2-100-3
8.56 8.08 8.08 8.08 8.06 8.06 8.06 8.05 8.05 8.03 8.02 7.74 7.73 7.72 7.71 7.71 7.70 7.69 7.48 7.48 7.46 7.46 7.46 7.44 7.44
2.50 DMSO-d6
O
OH
OH
Cl
3n
1.00
1.03
0.91
1.05
1.04
f1 (ppm)

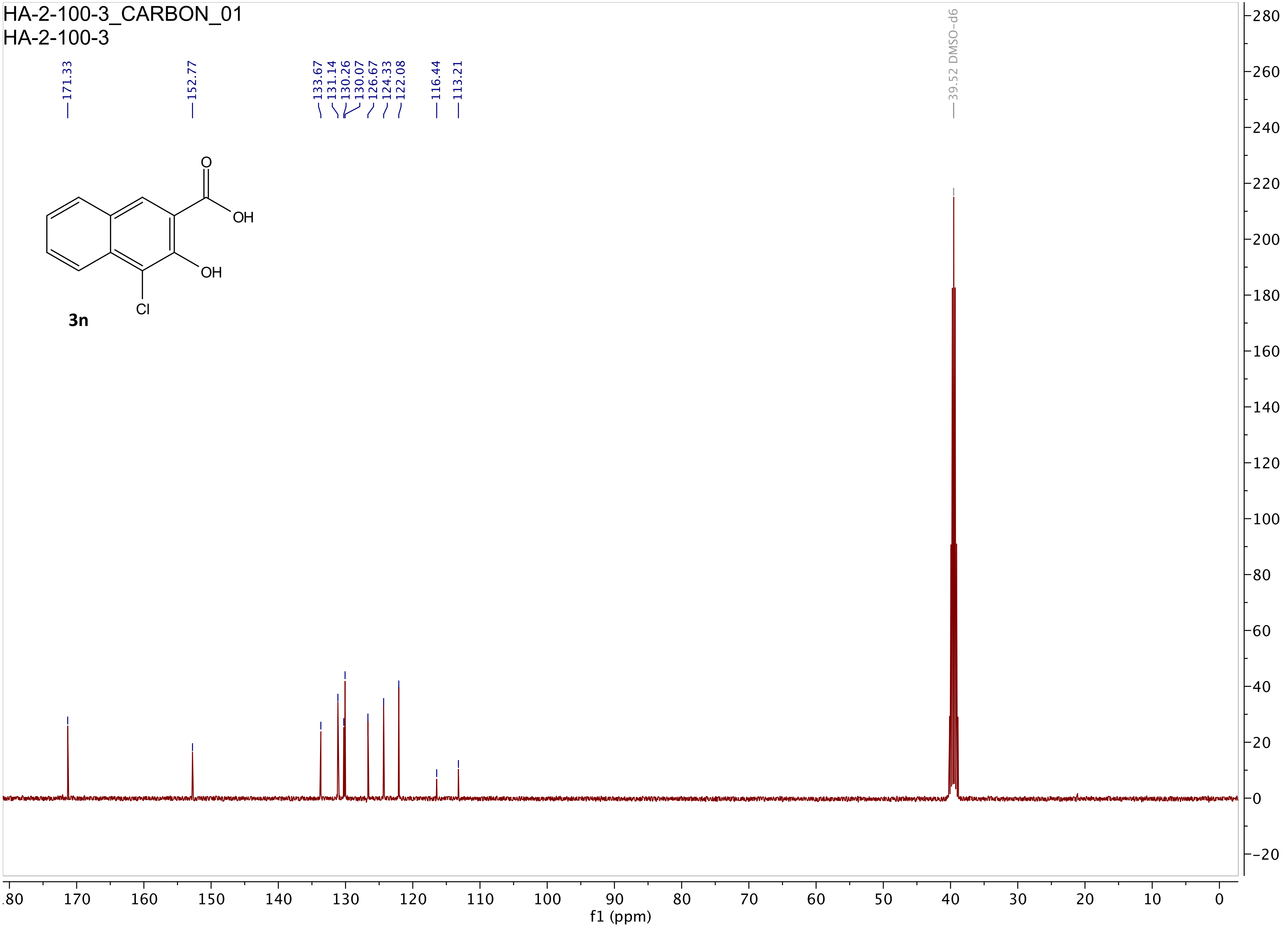
HA-2-100-3_CARBON_01
HA-2-100-3
171.33
152.77
133.67
131.14
130.26
130.07
126.67
124.33
122.08
116.44
113.21
39.52 DMSO-d6
O
OH
OH
Cl
3n
f1 (ppm)

HA-2-111-2_PROTON_U4DD2_01
HA-2-111-2

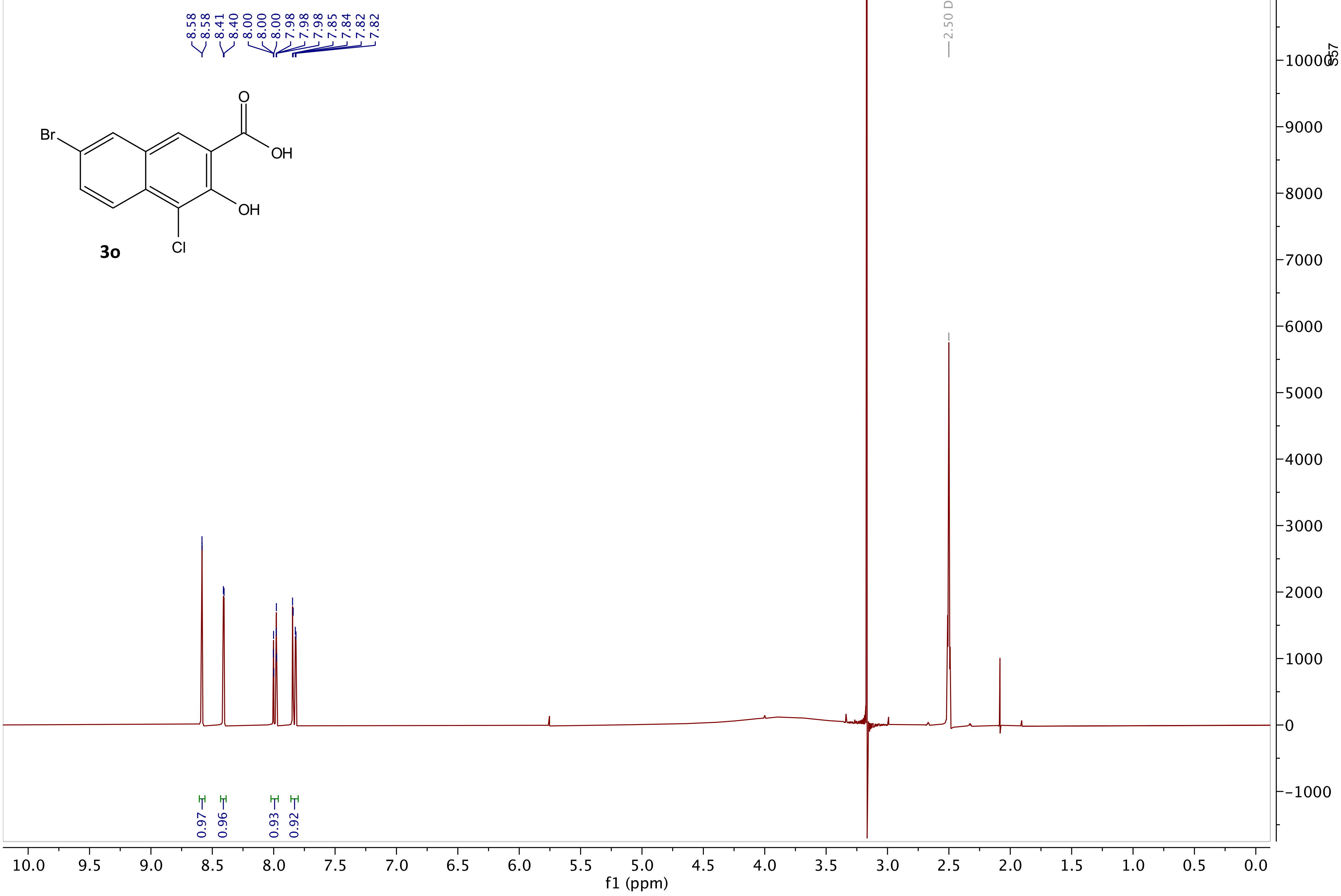

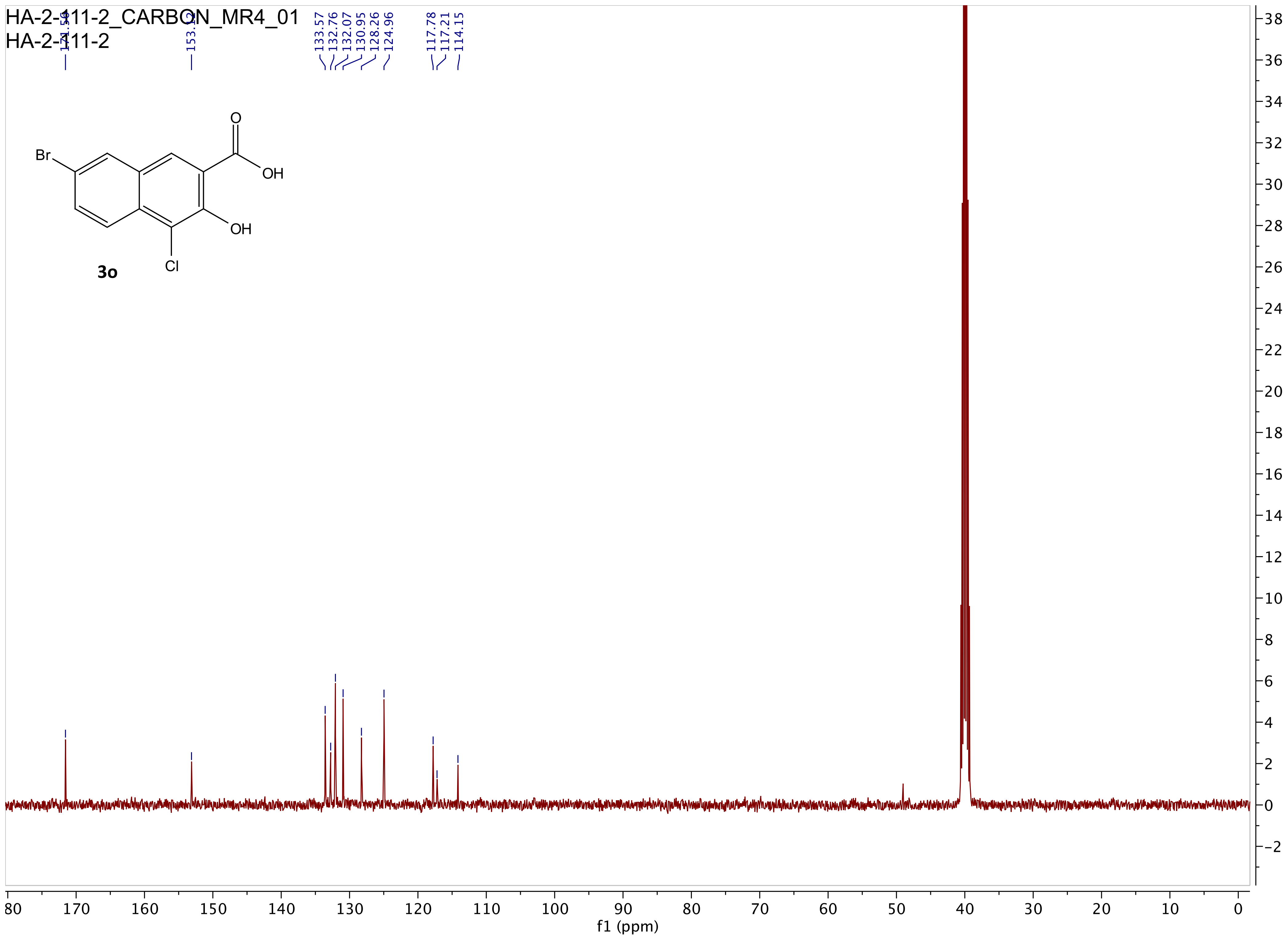
HA-2-111-2_CARBON_MR4_01
HA-2-111-2
133.57
132.76
132.07
130.95
128.26
124.96
117.78
117.21
114.15
Br
O
OH
OH
Cl
3o
f1 (ppm)

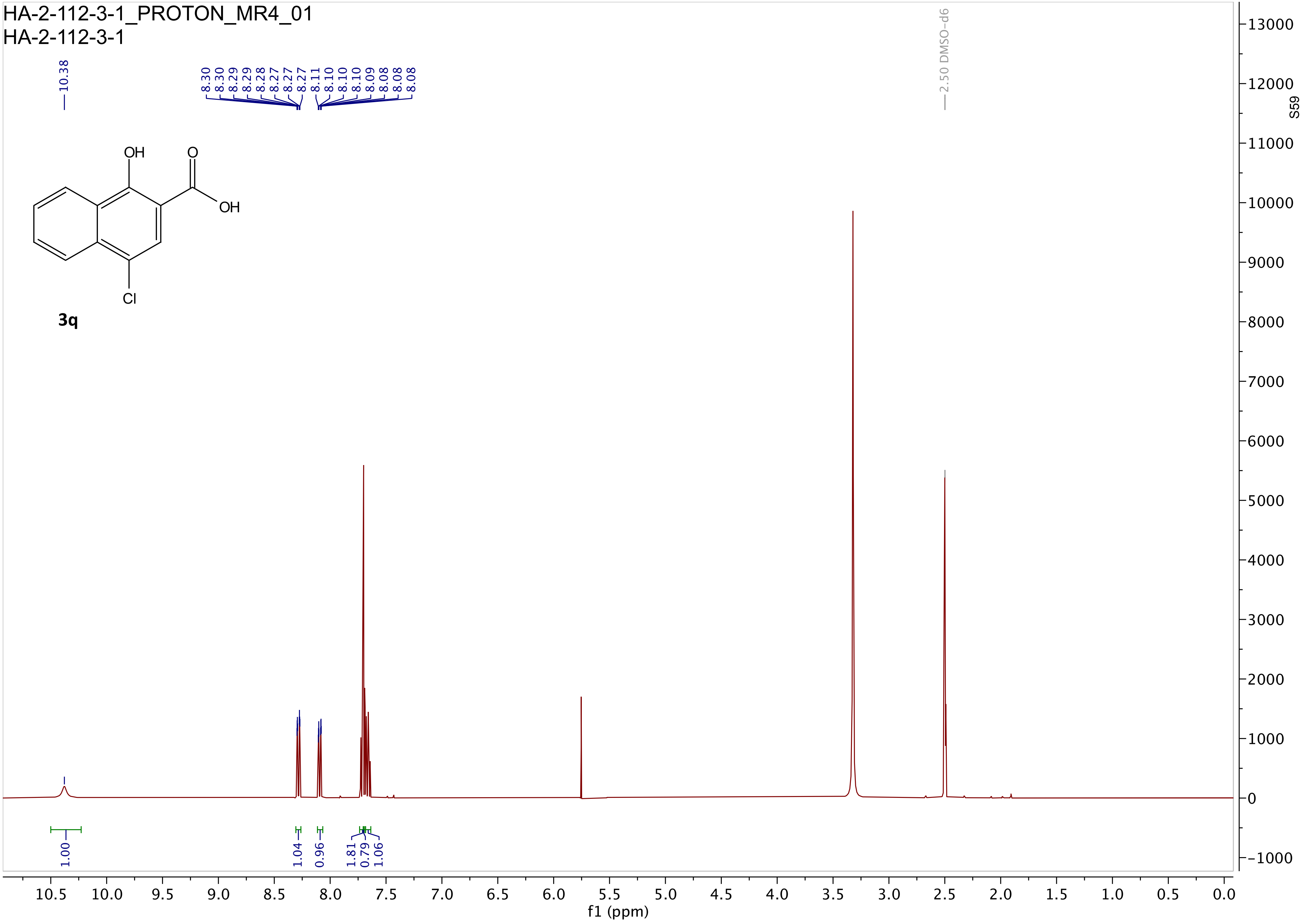
HA-2-112-3-1_PROTON_MR4_01
HA-2-112-3-1
10.38
8.30
8.30
8.29
8.29
8.28
8.27
8.27
8.27
8.11
8.10
8.10
8.10
8.09
8.08
8.08
8.08
2.50 DMSO-d6
OH
O
OH
Cl
3q
1.00
1.04
0.96
1.81
0.79
1.06
f1 (ppm)

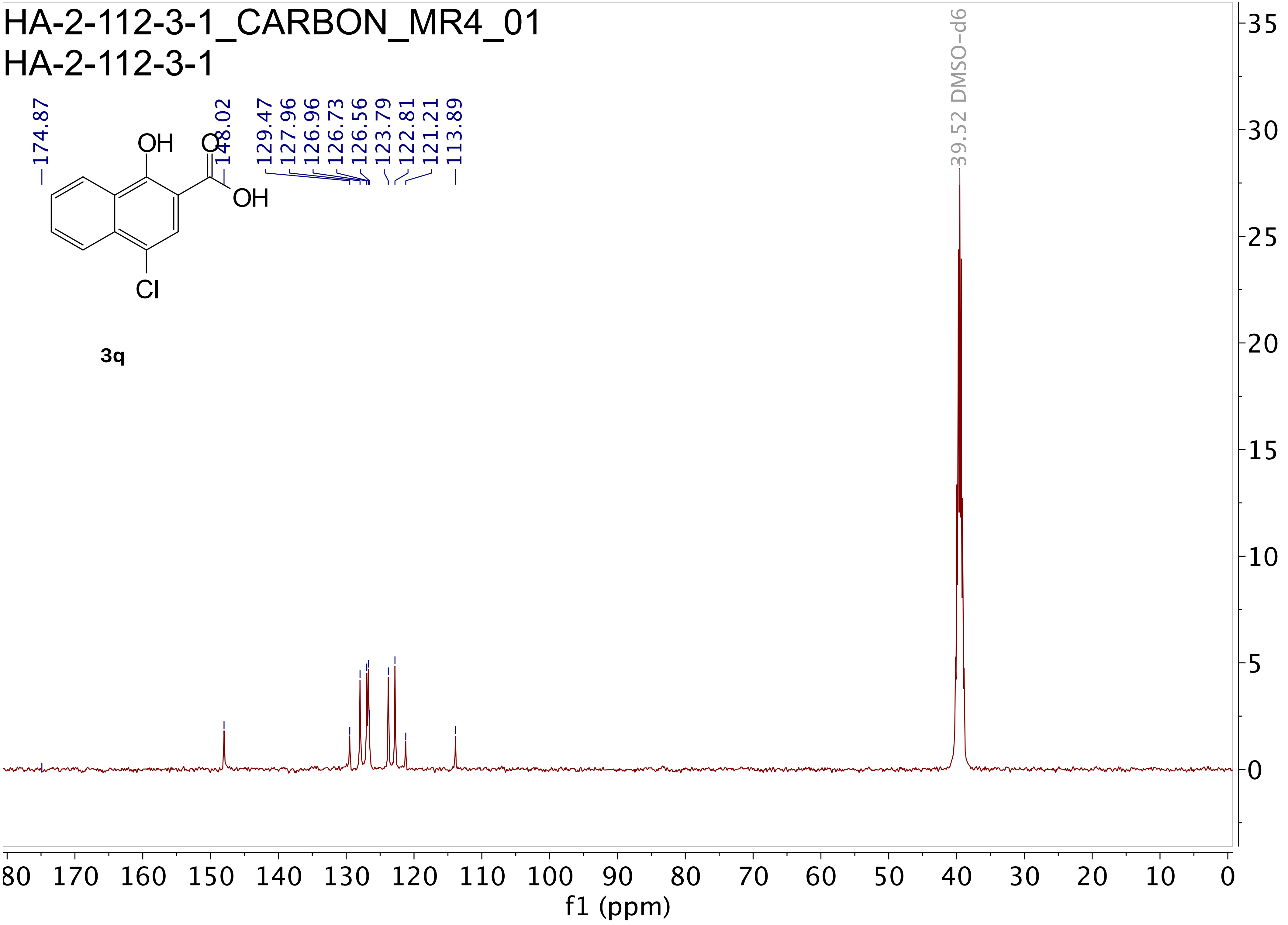
HA-2-112-3-1_CARBON_MR4_01
HA-2-112-3-1
174.87
148.02
129.47
127.96
126.96
126.73
126.56
123.79
122.81
121.21
113.89
39.52 DMSO-d6
OH
O
OH
Cl
3q
f1 (ppm)

HA-2-122-4-1_PROTON_MR4_01
HA-2-122-4-1

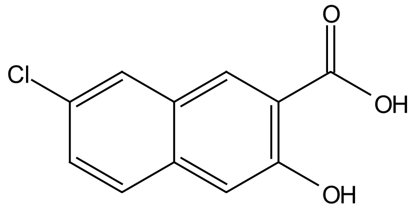

3p

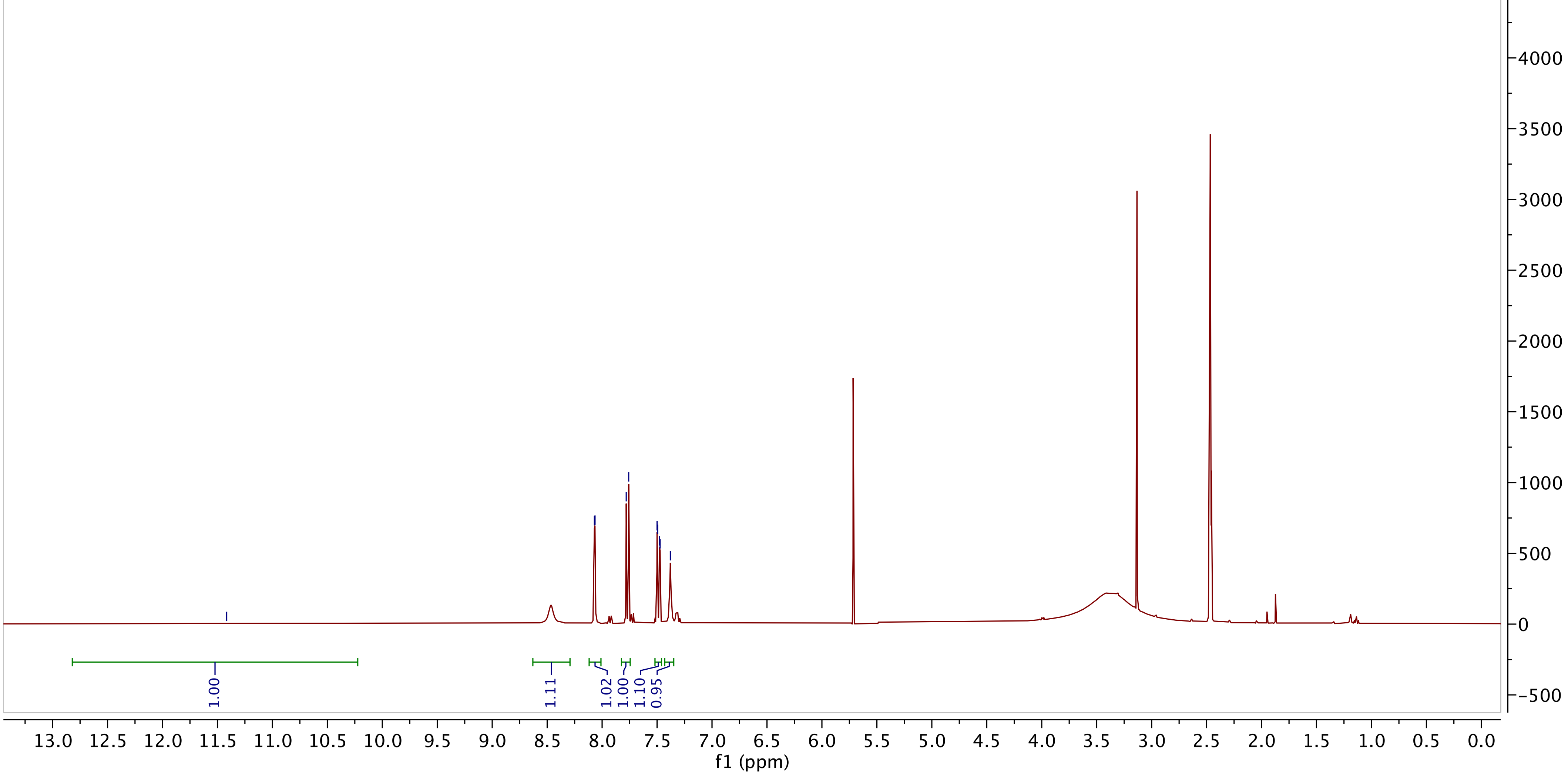

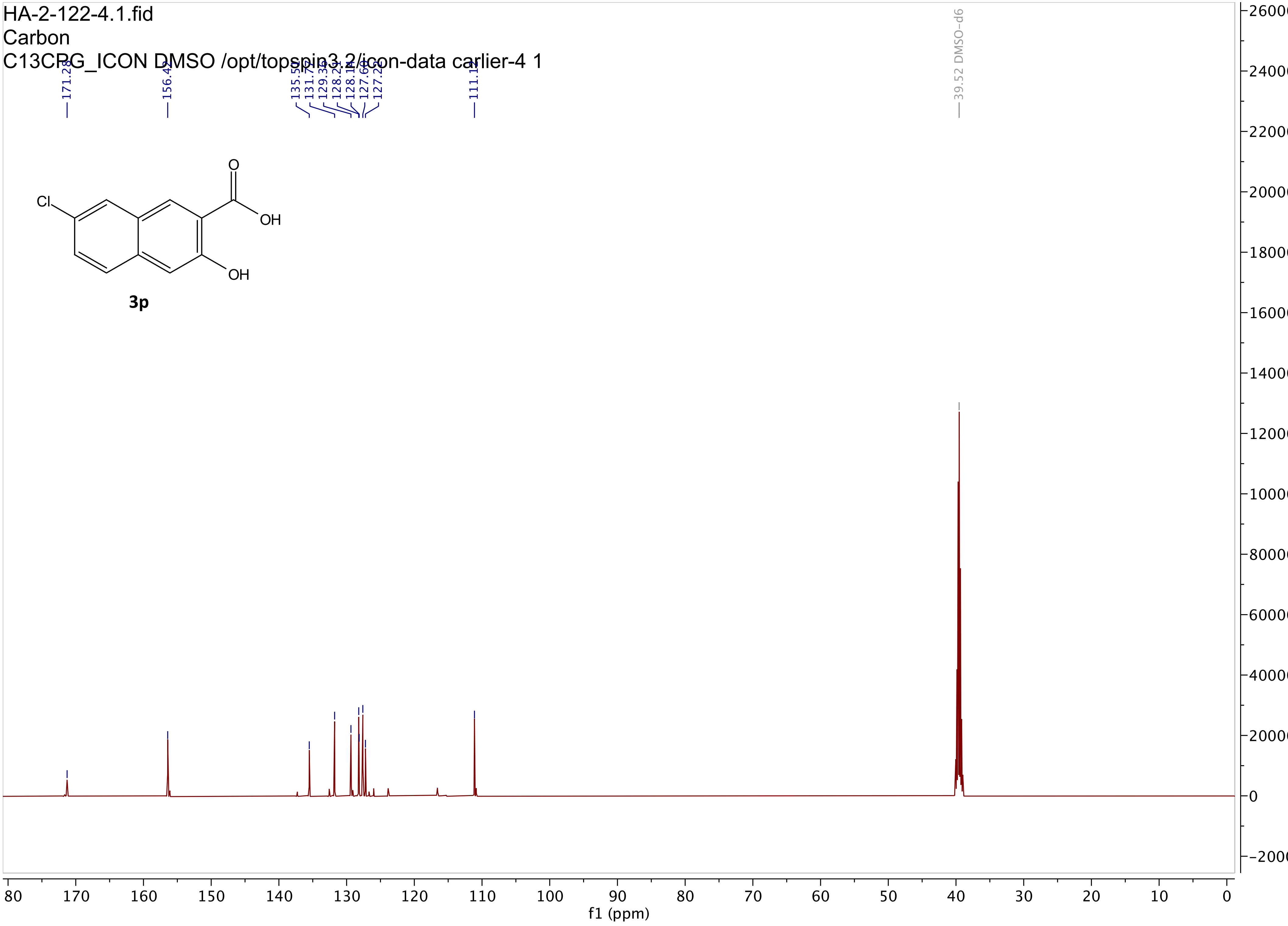
HA-2-122-4.1.fid
Carbon
C13CPG_ICON DMSO /opt/topspin3.2/icon-data carlier-4 1
171.28
156.42
135.5
131.7
129.3
128.2
128.1
127.6
127.2
111.1
39.52 DMSO-d6
Cl
O
OH
OH
3p
f1 (ppm)

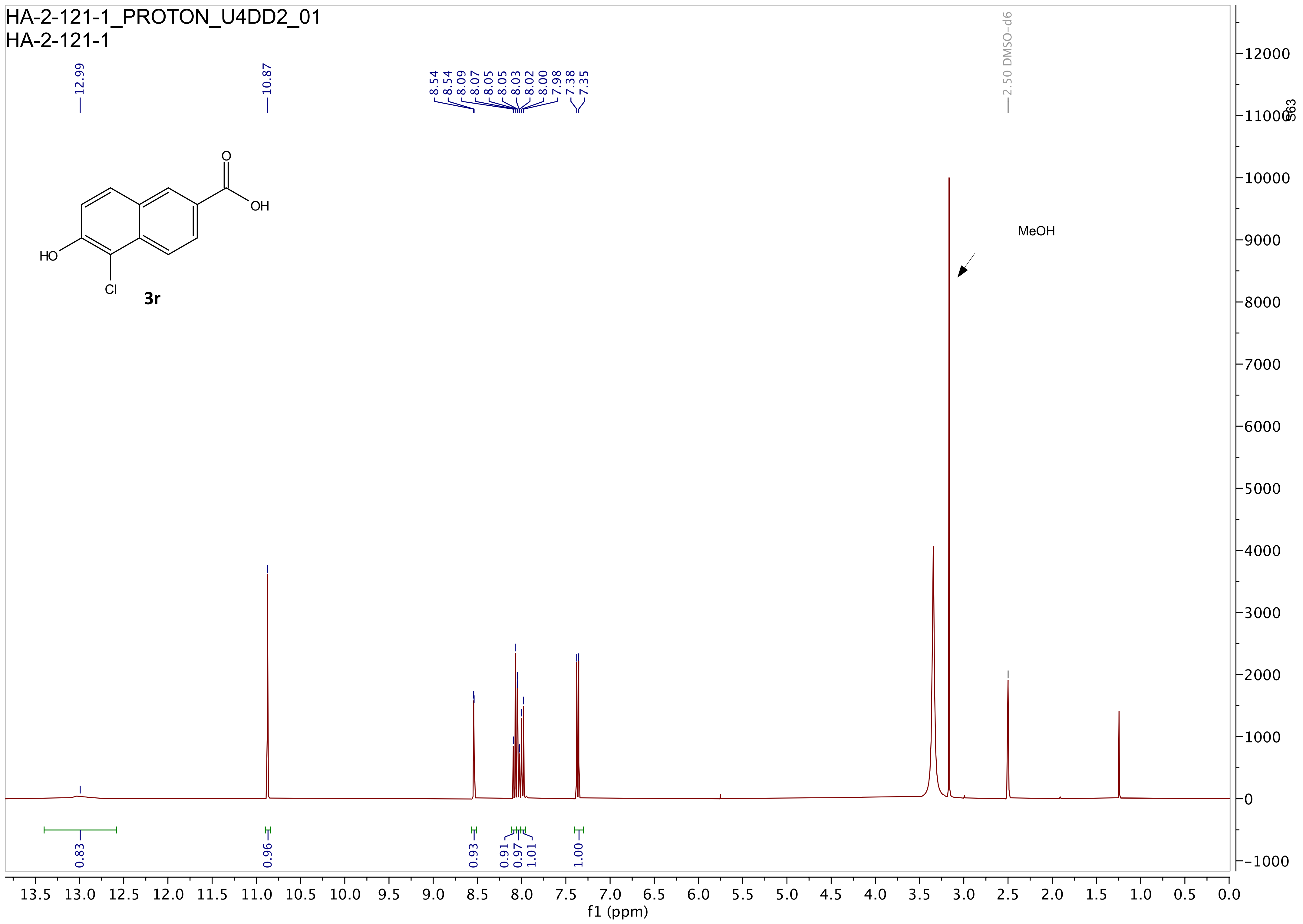

HA-2-121-1_PROTON_U4DD2_01
HA-2-121-1
12.99
10.87
8.54
8.54
8.09
8.07
8.05
8.05
8.03
8.02
8.00
7.98
7.38
7.35
2.50 DMSO-d6
O
OH
HO
Cl
MeOH
0.83
0.96
0.93
0.91
0.97
1.01
1.00
f1 (ppm)

3r

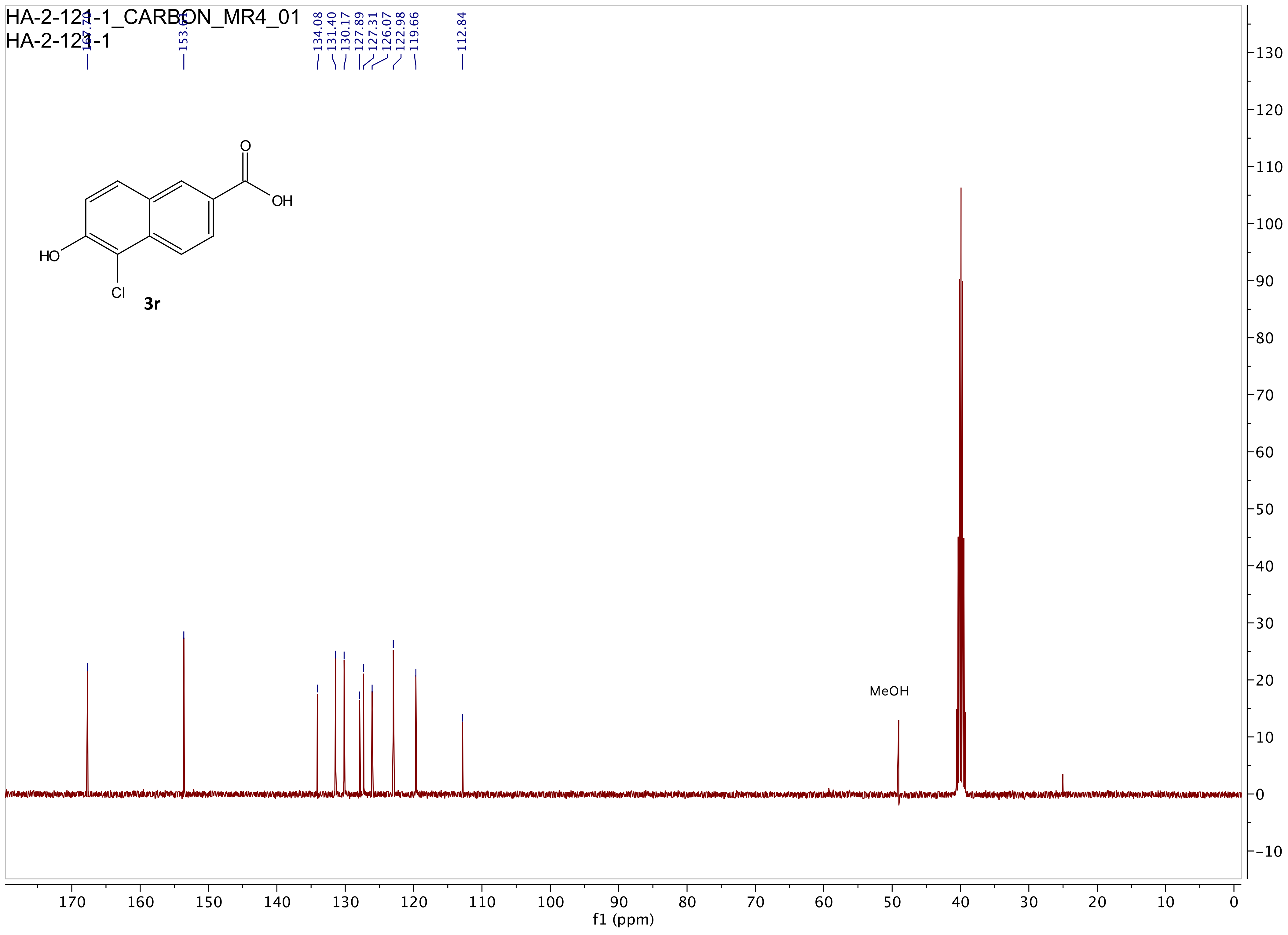
HA-2-121-1_CARBON_MR4_01
HA-2-121-1
167.70
153.61
134.08
131.40
130.17
127.89
127.31
126.07
122.98
119.66
112.84
O
OH
HO
Cl
3r
MeOH
f1 (ppm)
170
160
150
140
130
120
110
100
90
80
70
60
50
40
30
20
10
0
-10

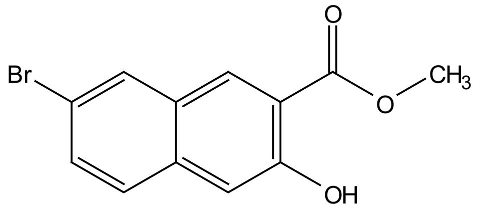
Br
O
O
CH3
OH

4b

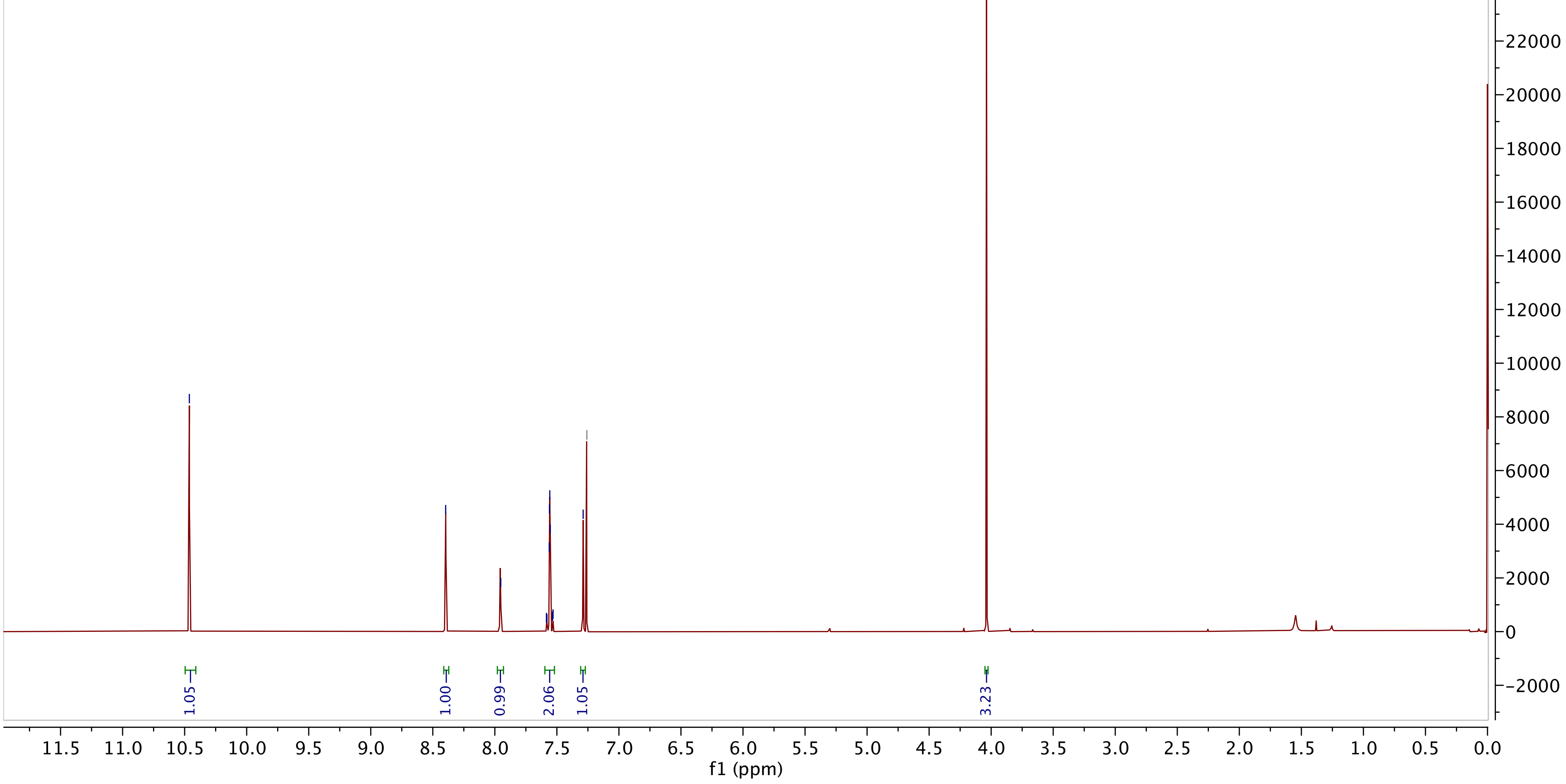
HA-2-79_PROTON_01
HA-2-79
10.46
8.40
7.95
7.58
7.58
7.56
7.56
7.56
7.55
7.53
7.53
7.29
7.26 CDCl3
4.04
1.05
1.00
0.99
2.06
1.05
3.23
11.5
11.0
10.5
10.0
9.5
9.0
8.5
8.0
7.5
7.0
6.5
6.0
5.5
5.0
4.5
4.0
3.5
3.0
2.5
2.0
1.5
1.0
0.5
0.0
f1 (ppm)
34000
32000
30000
28000
26000
24000
22000
20000
18000
16000
14000
12000
10000
8000
6000
4000
2000
0
−2000

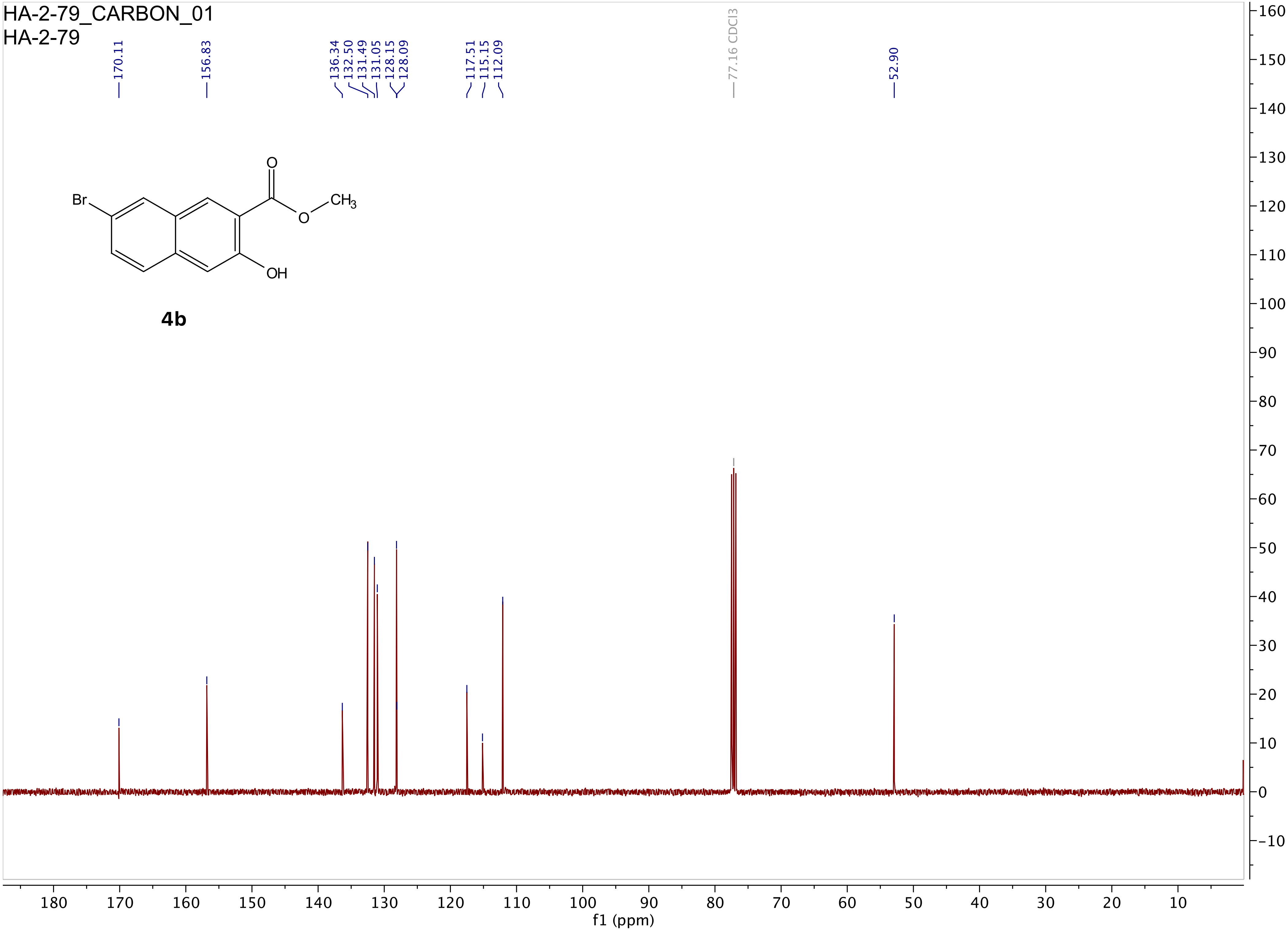

HA-2-79_CARBON_01
HA-2-79
170.11
156.83
136.34
132.50
131.49
131.05
128.15
128.09
117.51
115.15
112.09
77.16 CDCl3
52.90
Br
O
O
CH3
OH
4b
f1 (ppm)

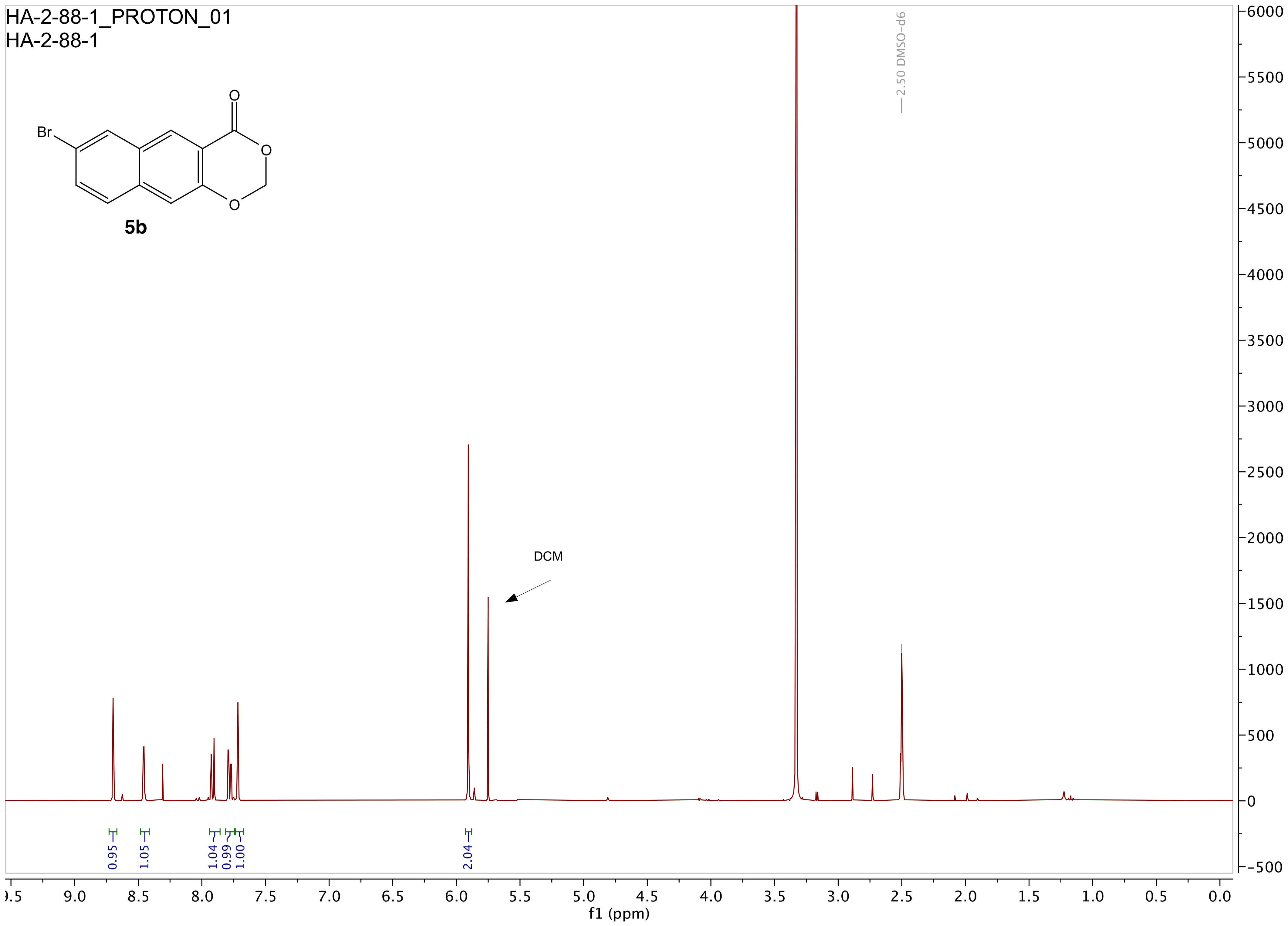

HA-2-88-1_PROTON_01
HA-2-88-1
Br
O
O
O
5b
DCM
2.50 DMSO-d6
0.95
1.05
1.04
0.99
1.00
2.04
f1 (ppm)

HA-2-88-1_CARBON_01
HA-2-88-1

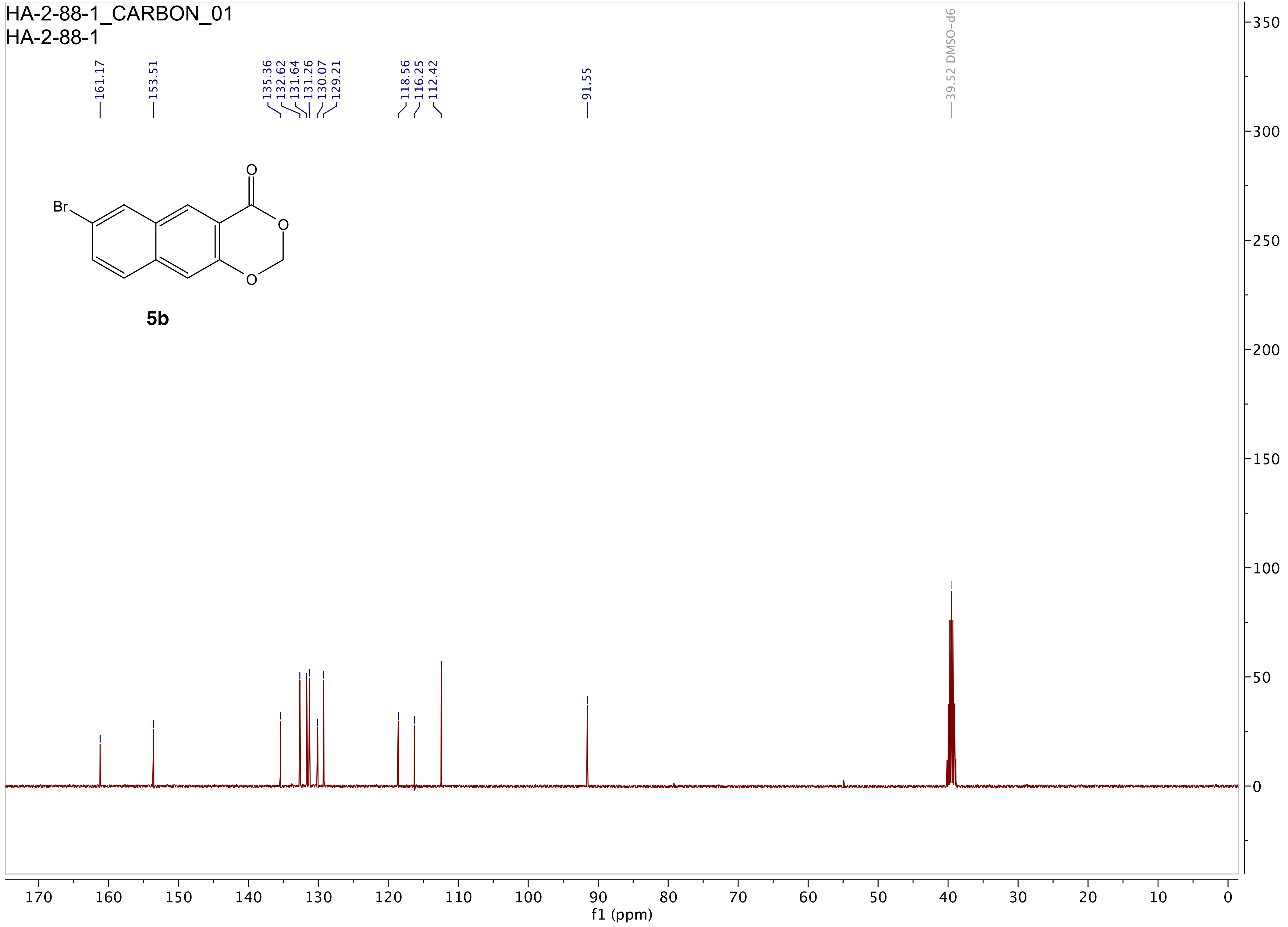

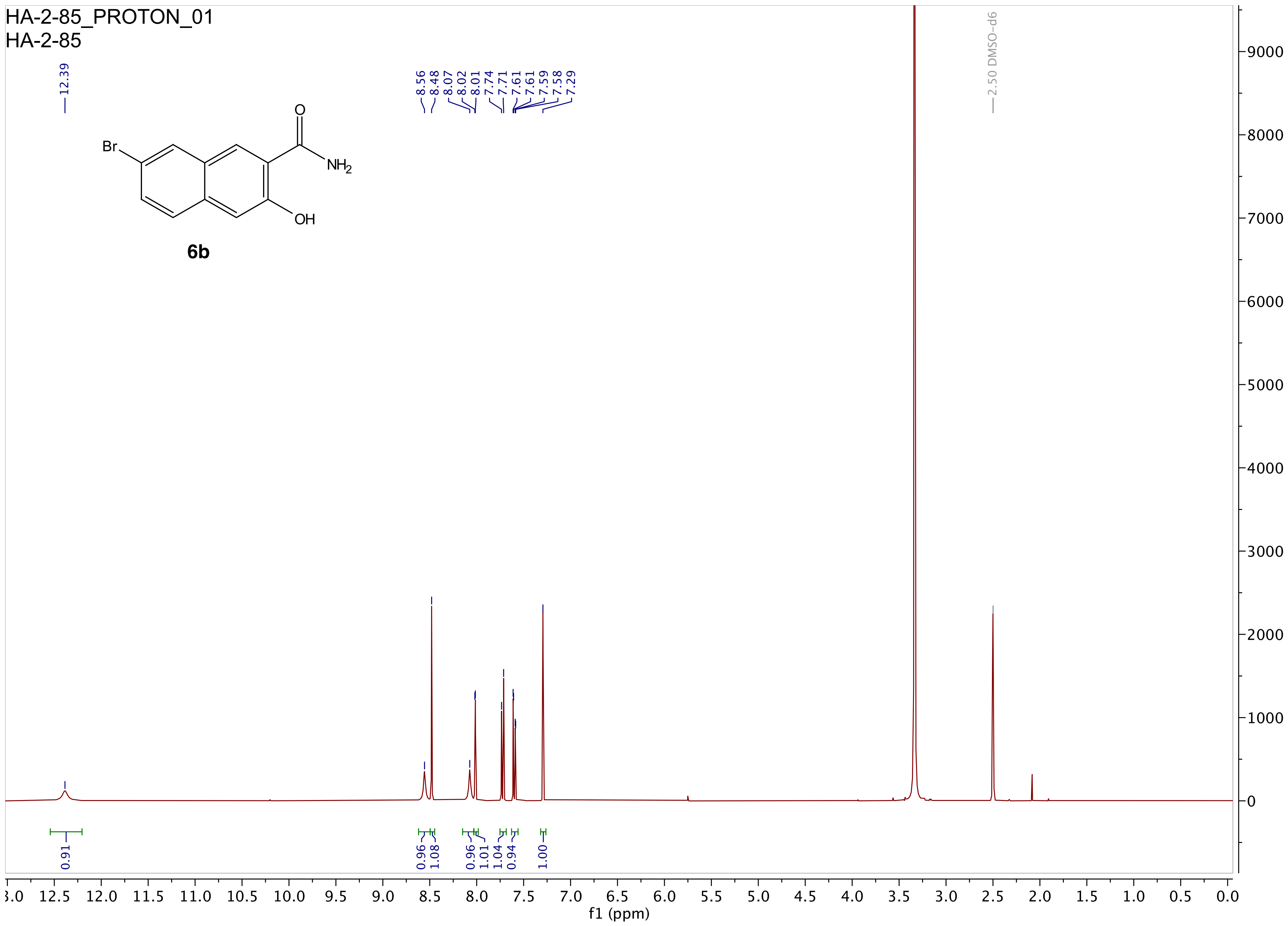
HA-2-85_PROTON_01
HA-2-85
12.39
8.56
8.48
8.07
8.02
8.01
7.74
7.71
7.61
7.61
7.59
7.58
7.29
2.50 DMSO-d6
Br
O
NH2
OH
6b
0.91
0.96
1.08
0.96
1.01
1.04
0.94
1.00
f1 (ppm)

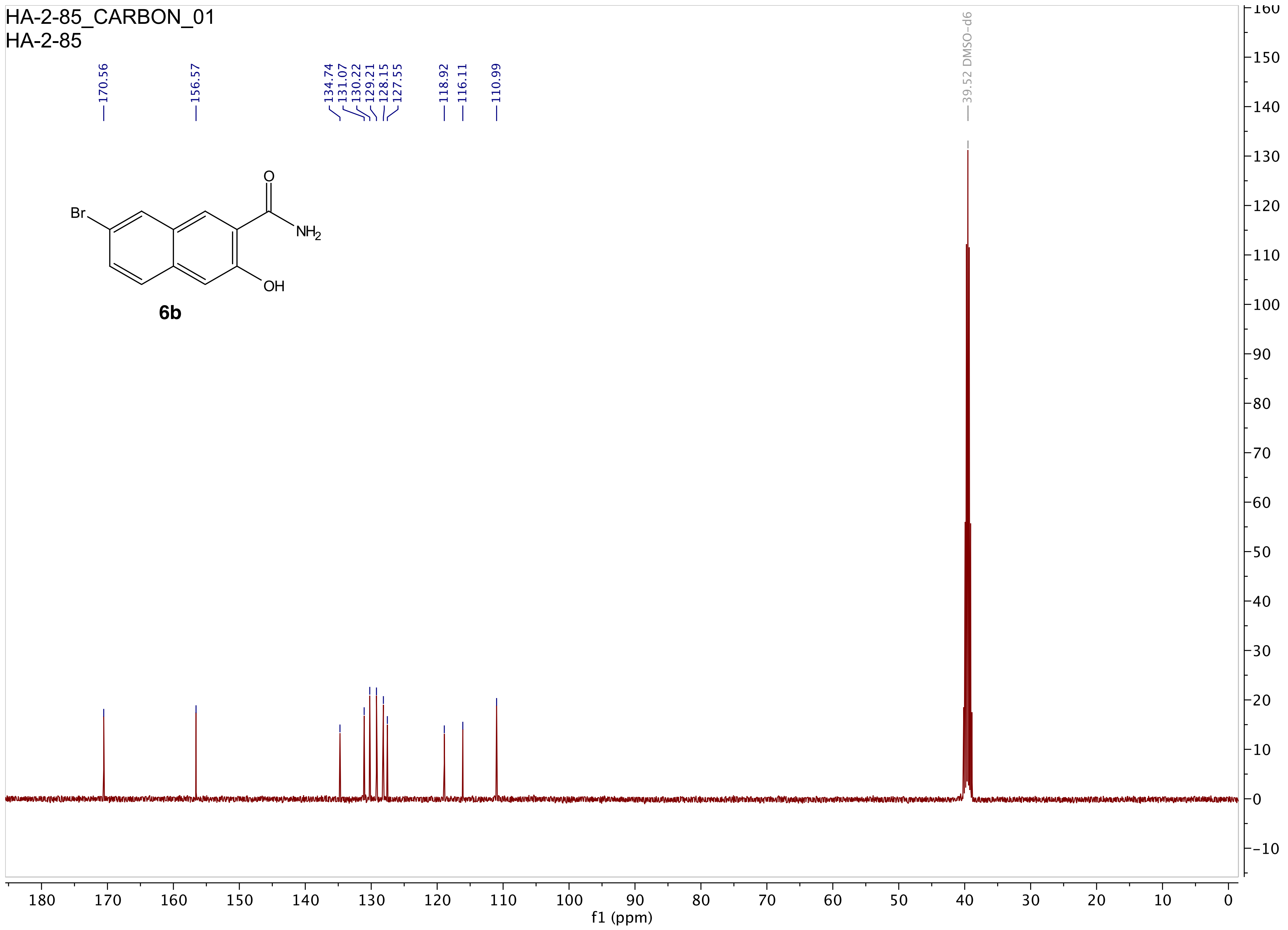

HA-2-85_CARBON_01
HA-2-85
170.56
156.57
134.74
131.07
130.22
129.21
128.15
127.55
118.92
116.11
110.99
39.52 DMSO-d6
Br
O
NH2
OH
6b
f1 (ppm)

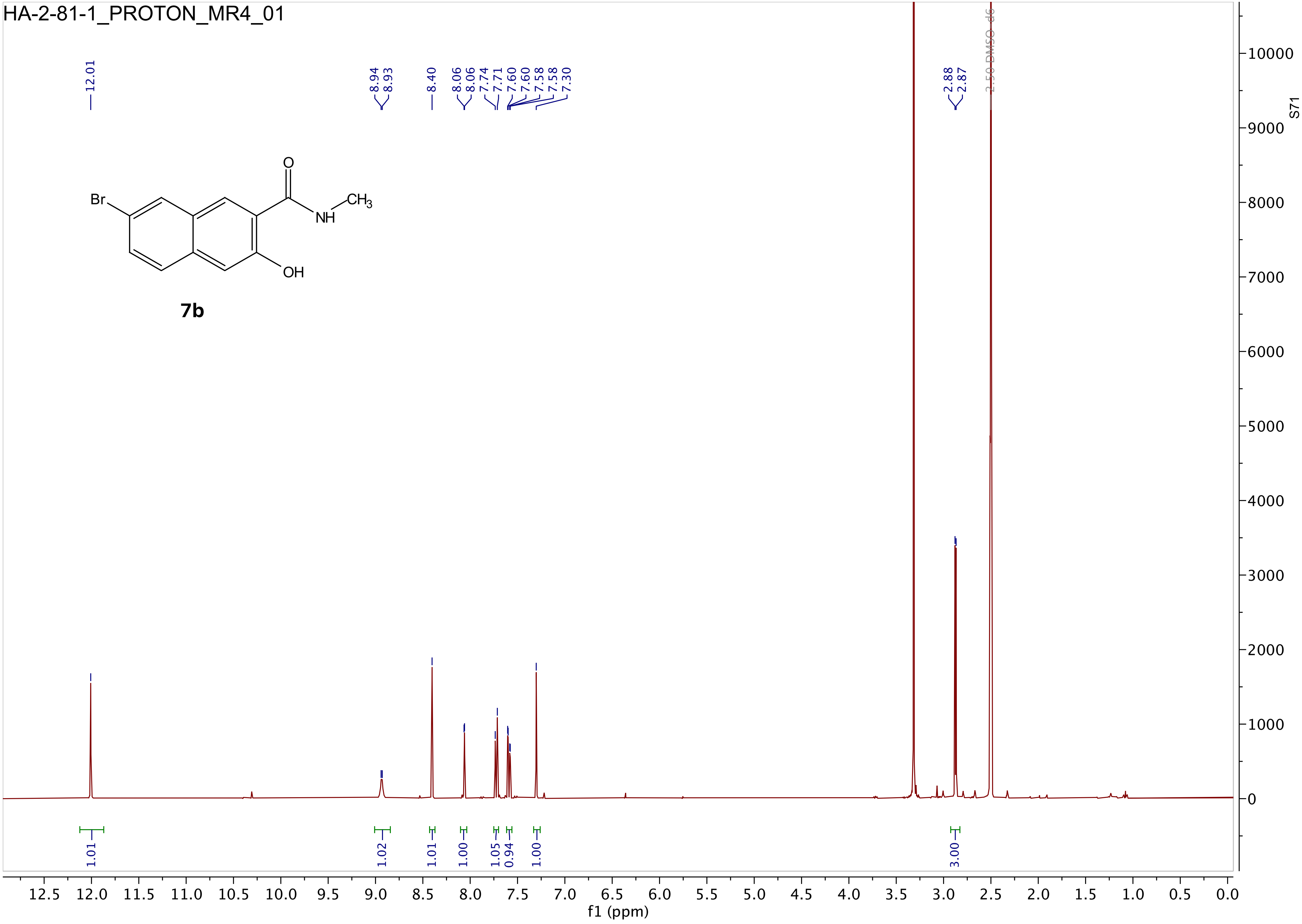
HA-2-81-1_PROTON_MR4_01
12.01
8.94
8.93
8.40
8.06
8.06
7.74
7.71
7.60
7.60
7.58
7.58
7.30
2.88
2.87
2.50 DMSO-d6
Br
O
NH
CH3
OH
7b
1.01
1.02
1.01
1.00
1.05
0.94
1.00
3.00
12.5 12.0 11.5 11.0 10.5 10.0 9.5 9.0 8.5 8.0 7.5 7.0 6.5 6.0 5.5 5.0 4.5 4.0 3.5 3.0 2.5 2.0 1.5 1.0 0.5 0.0
f1 (ppm)
10000 9000 8000 7000 6000 5000 4000 3000 2000 1000 0

HA-2-81-1.2.fid
PROTON
C13CPG_ICON DMSO /opt/topspin3.2/icon-data carlier-4 3

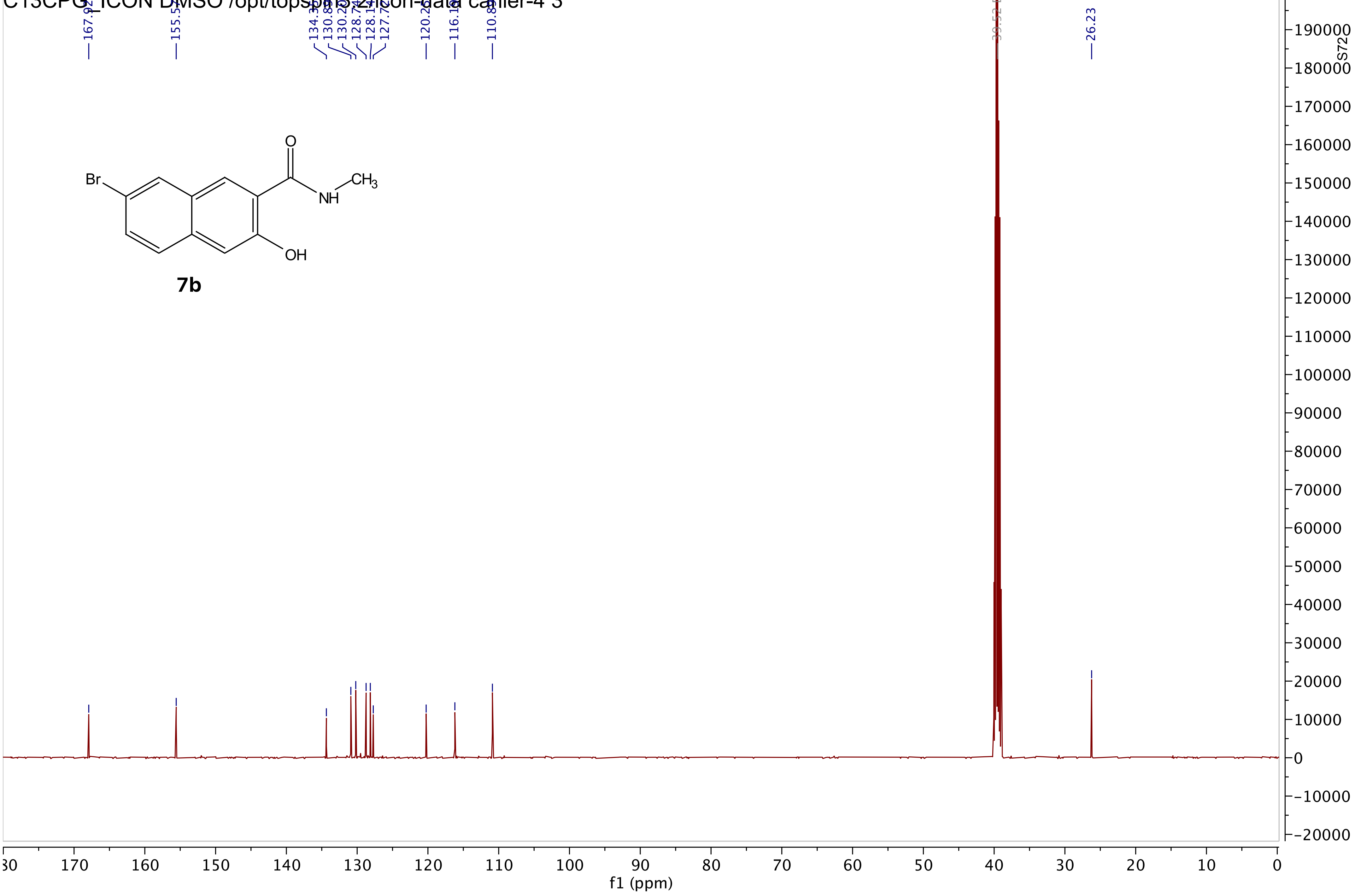

7b

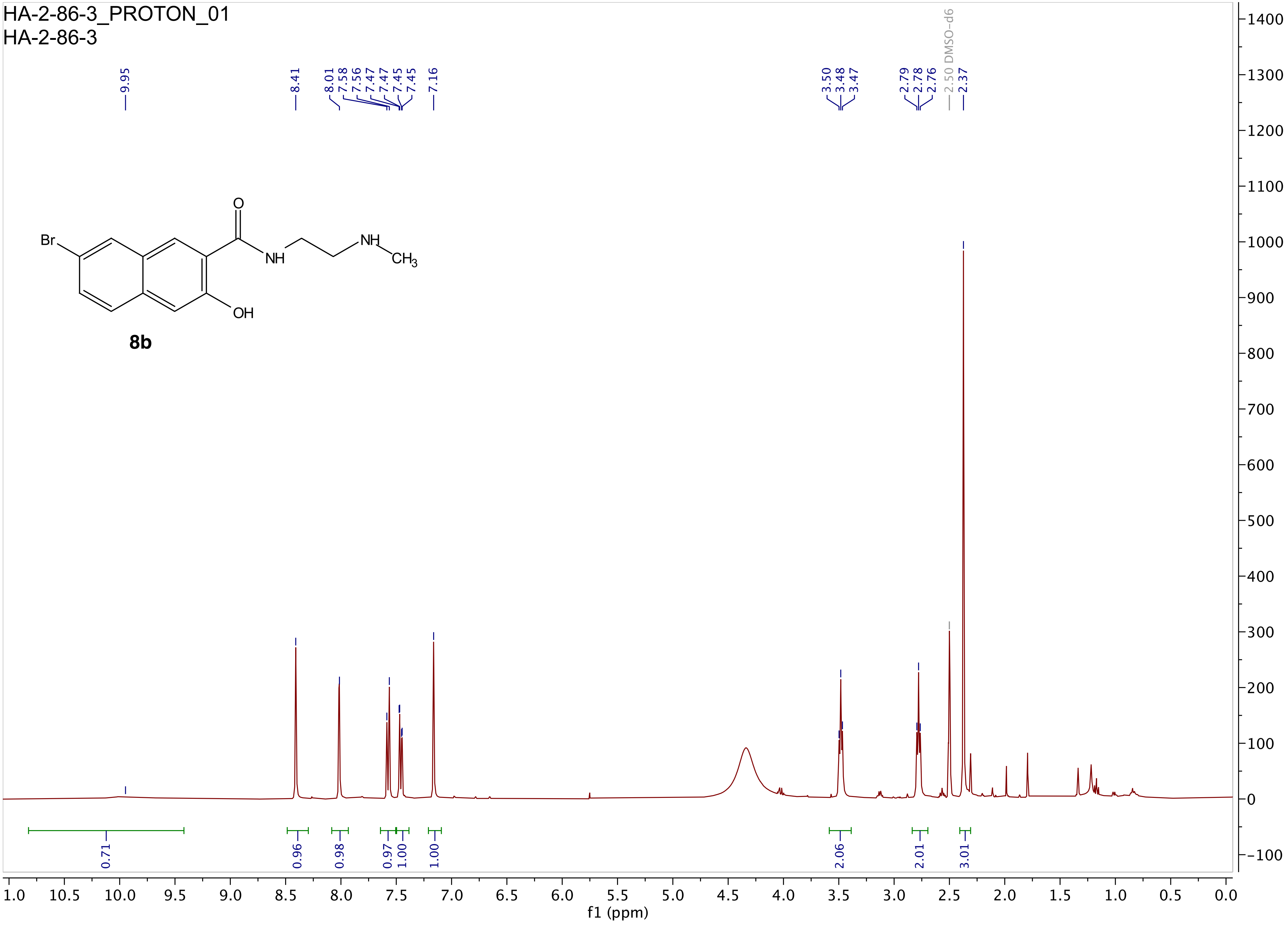
HA-2-86-3_PROTON_01
HA-2-86-3
9.95
8.41
8.01
7.58
7.56
7.47
7.47
7.45
7.45
7.16
3.50
3.48
3.47
2.79
2.78
2.76
2.50 DMSO-d6
2.37
Br
O
NH
NH
CH3
OH
8b
0.71
0.96
0.98
0.97
1.00
1.00
2.06
2.01
3.01
f1 (ppm)

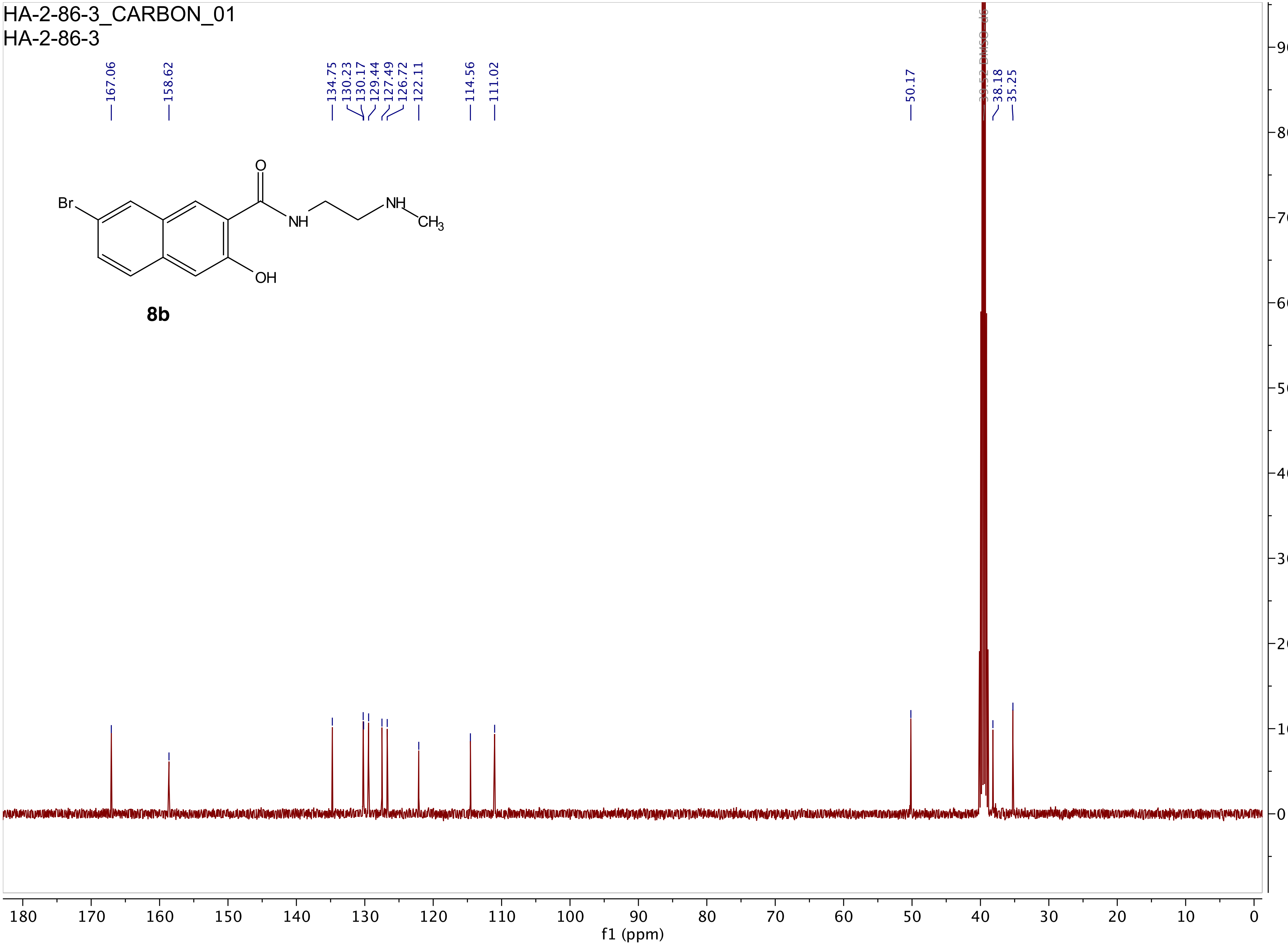

HA-2-86-3_CARBON_01
HA-2-86-3
167.06
158.62
134.75
130.23
130.17
129.44
127.49
126.72
122.11
114.56
111.02
50.17
39.52 DMSO-d6
38.18
35.25
Br
O
NH
NH
CH_3
OH
8b
f1 (ppm)

HA-2-76-3.1.fid
PROTON
PROTONRO_ICON DMSO /opt/topspin3.2/icon-data carlier-4 1

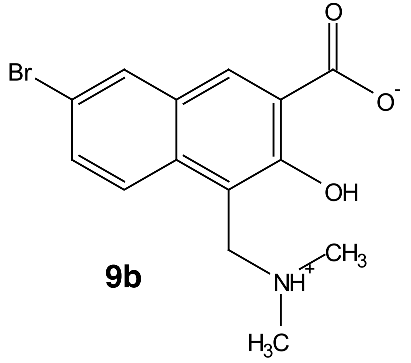

9b

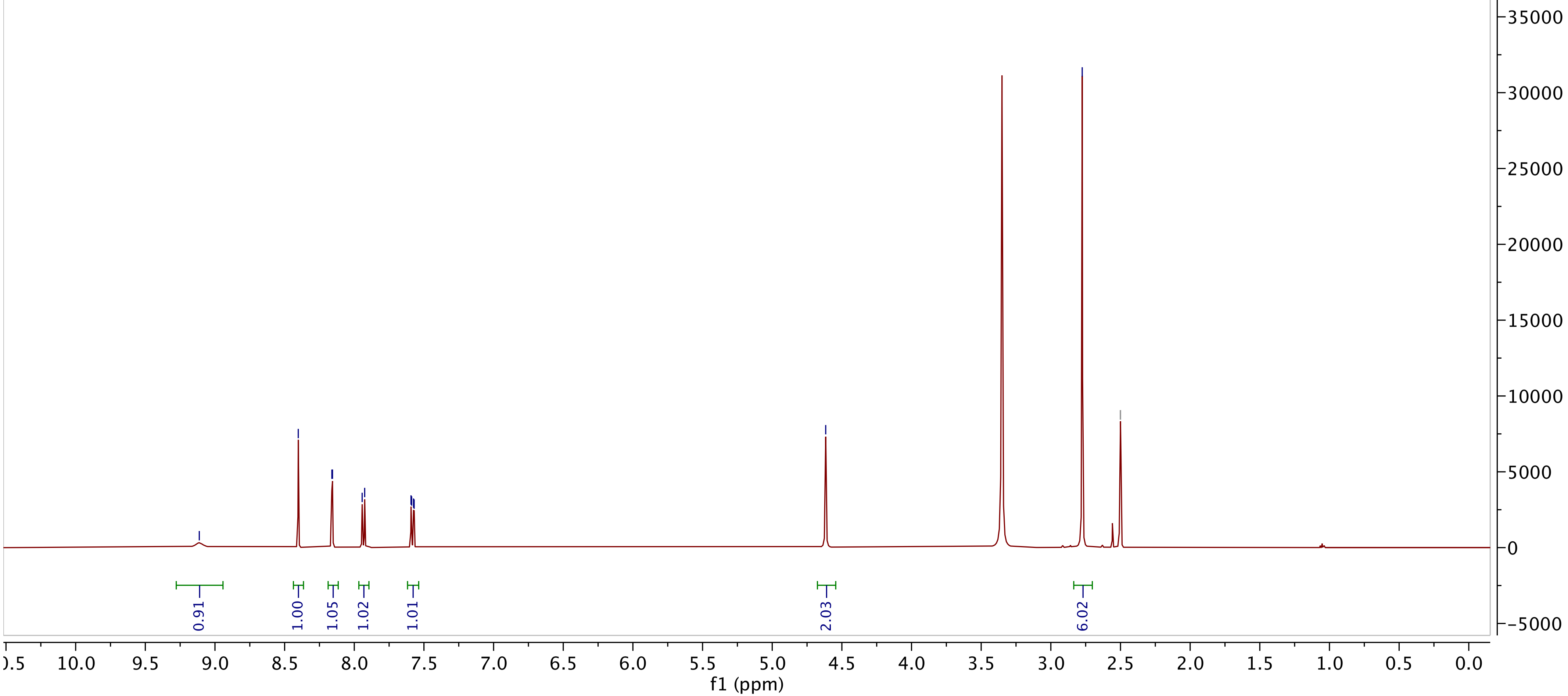

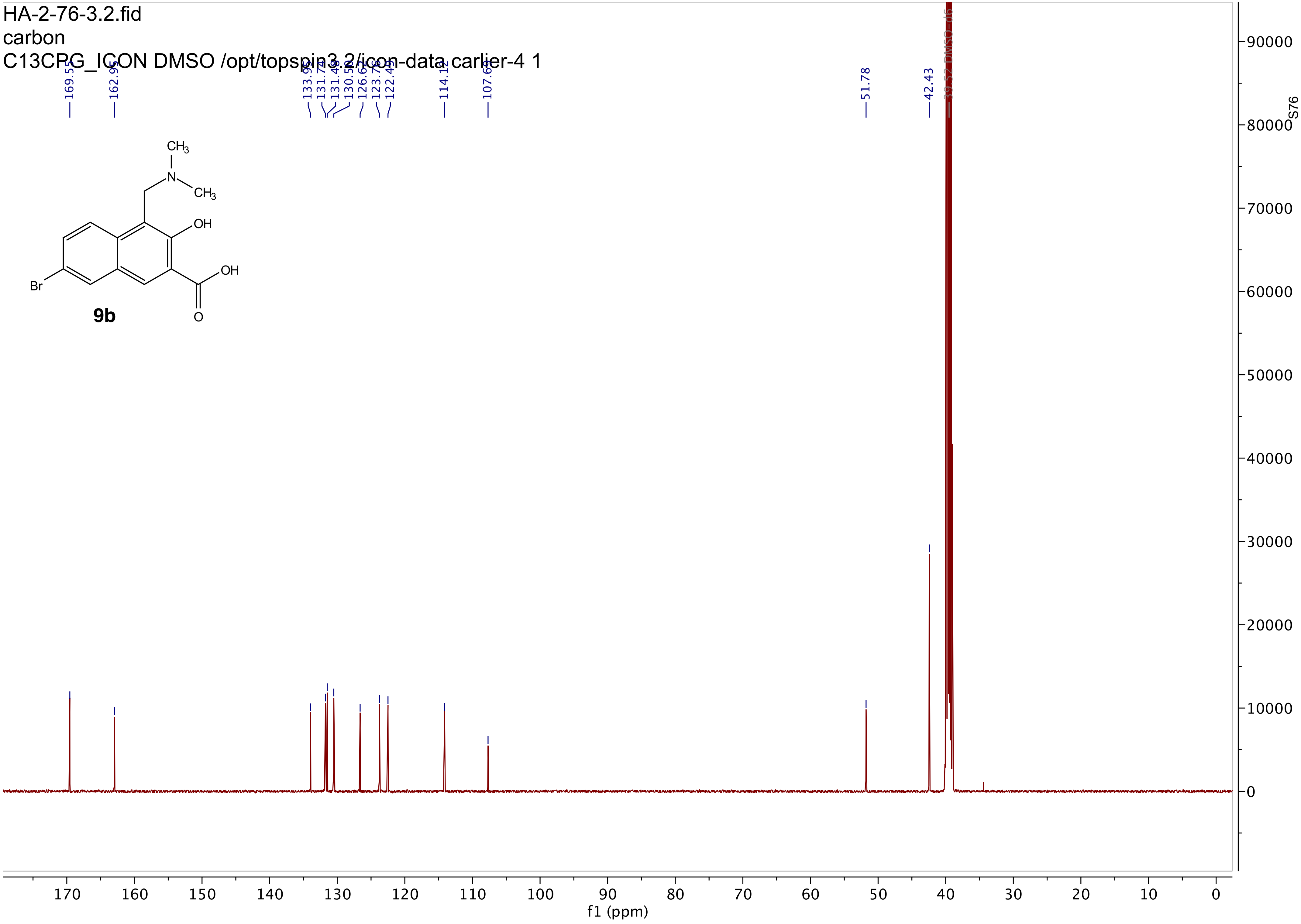

HA-2-76-3.2.fid
carbon
C13CPG_ICON DMSO /opt/topspin3.2/icon-data carlier-4 1
169.55
162.95
133.95
131.74
131.49
130.50
126.63
123.76
122.45
114.12
107.63
51.78
42.43
39.52 DMSO-d6
Br
OH
OH
O
N
CH3
CH3
9b
f1 (ppm)
